# N‐Acenoacenes: Synthesis and Solid‐State Properties

**DOI:** 10.1002/chem.202201916

**Published:** 2022-10-25

**Authors:** Thomas Wiesner, Marcel Pardon, Steffen Maier, Frank Rominger, Jan Freudenberg, Uwe H. F. Bunz

**Affiliations:** ^1^ Organisch-Chemisches Institut Ruprecht-Karls-Universität Heidelberg Im Neuenheimer Feld 270 69120 Heidelberg Germany; ^2^ Centre for Advanced Materials (CAM) Im Neuenheimer Feld 225 69120 Heidelberg Germany

**Keywords:** azaacenes, bisacenes, crystal engineering, semiconductors, solid-state packing

## Abstract

Four N‐acenoacenes were synthesized and analyzed for their optoelectronic properties and solid‐state packings. Two of the regioisomeric acridinoacridines are TIPS‐ethynylated, whereas the other pair are Boc‐ and triflate substituted derivatives. The two TIPS‐ethynyldiazaacenoacenes were processed into organic thin‐film transistors with saturation hole mobilities reaching 2.9×10^−2^ cm^2^(Vs)^−1^.

## Graphical abstract

**Enabling semiconducting** properties in N‐acenoacenes. Reducing the steric demand of N‐acenoacenes tunes the solid‐state packings of N‐acenoacenes in favor of π–π‐stacking, allowing charge transfer through the crystal lattice. The first utilization of N‐acenoacenes in p‐type organic thin‐film transistors is reported.

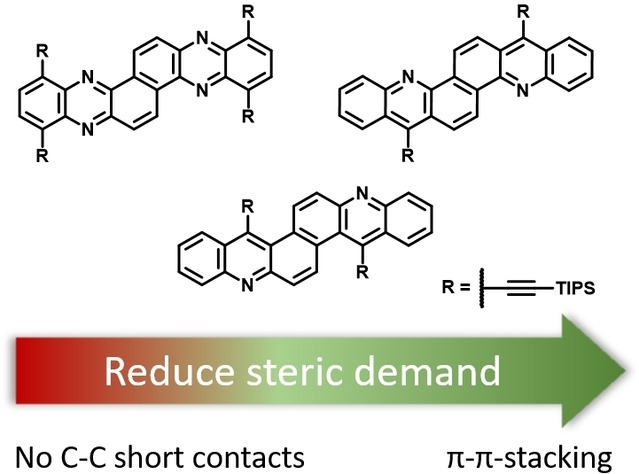

## Introduction

**Figure 1:**
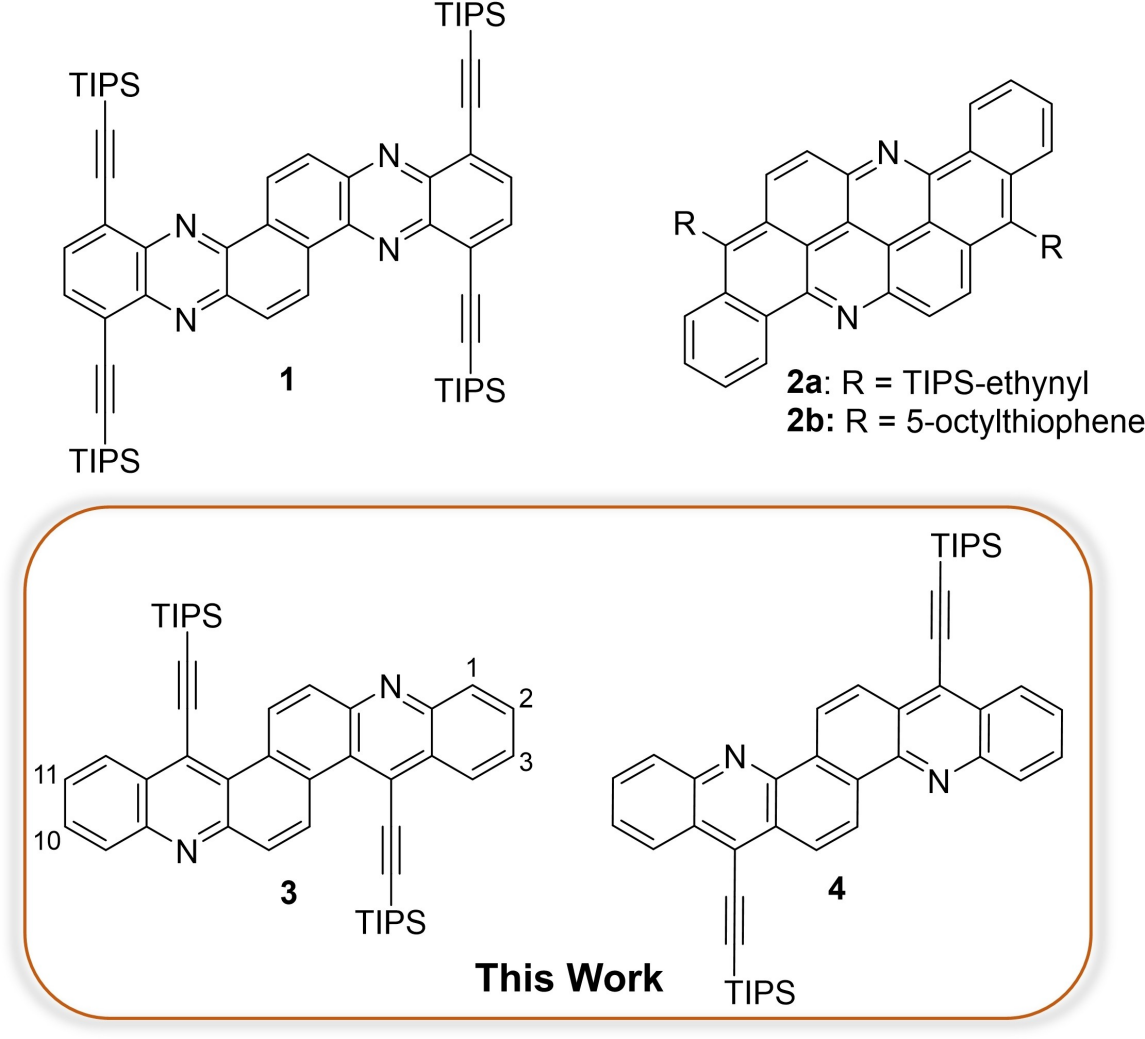
Structures of the bisacenes **3** and **4**, their parent compound **1** ^[7]^ and flavanthrone‐derived bisacenes **2 a**,**b**.^[8]^

The synthesis and application of bisacenes^[1]^ is of interest as they are more stable than their acene congeners^[2]^ and thus promising for organic field‐effect transistors (OFETs).^[3]^ Numerous works on the syntheses of π‐extended acridines^[4]^ and bisacridines^[5]^ with mostly phenyl substituted pyridinic rings have been published. Yet, nitrogen‐doped azabisacenes^[6]^ such as **1** ^[7]^ and **2 a**,**b** ^[8]^ (Figure 1) are less explored. Aside from a single crystal OFET of **2 a**,^[8a]^ N‐acenoacenes also remain underinvestigated in OFET devices.

In this article, we present a synthetic access to TIPS‐ethynylated aza‐acenoacenes. They display reduced steric demand compared to **1** – they are substituted with only two morphology‐controlling TIPS‐ethynyl substituents, similar to their parent azaacenes.^[9]^ X‐ray crystallography unveils packing arrangements of **3** and **4** conducive for applications as OFET materials.

## Results and Discussion

**Scheme 1:**
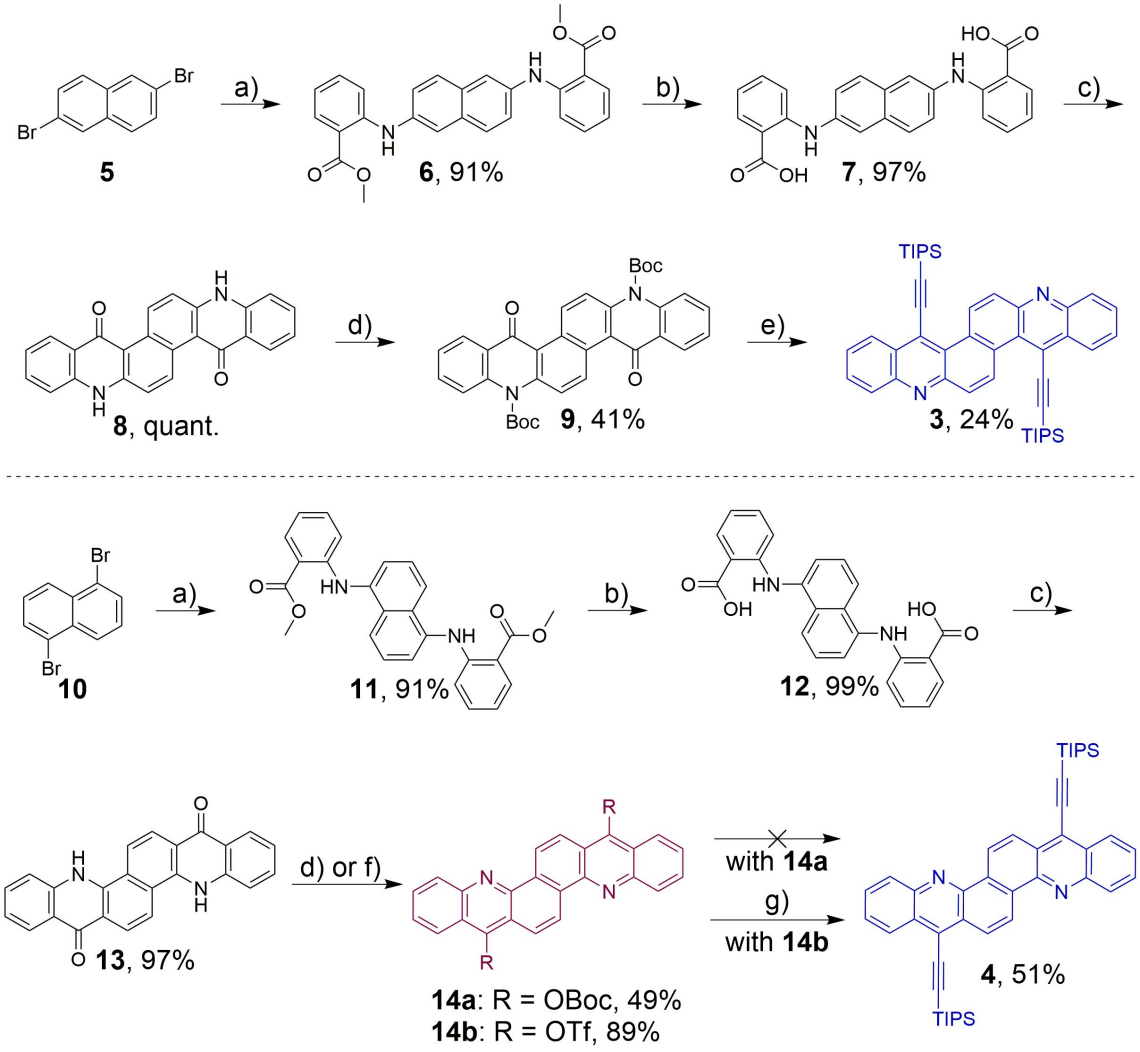
Synthetic routes towards **3** and **4**. a) Methyl anthranilate, Cs_2_CO_3_, RuPhos‐Pd G1, b) KOH, THF/MeOH, c) Eaton's reagent,^[11]^ d) Boc_2_O, DMAP, 2,6‐lutidine, e) 1. *n*‐BuLi, TIPS‐acetylene, 2. MeI, 3. TFA, f) Tf_2_O, DMAP, 2,6‐lutidine, g) TIPS−C≡C‐SnMe_3_, Pd_2_(dba)_3_, *t*‐Bu_3_PHBF_4_.

Pyridinic bisazaacenes **3** and **4** are accessible by i) Buchwald‐Hartwig amination of naphthalene **5** or **10**, ii) saponification of **6** or **11** and subsequent iii) twofold intramolecular Friedel‐Crafts acylation (Scheme 1). Bisacridones **8** and **13** were obtained in high yields (60 %–88 % over 3 steps) starting from dibromonaphthalenes **5** or **10**. We note that intramolecular acylation was only successful starting from a naphthalene core: 1,5‐naphthyridine yielded intractable and insoluble products in the ring closing reaction (see Supporting Information).

From here on, the synthetic access diverged: Although **8** and **13** were subjected to Boc‐protection, only **9** was obtained as the expected N,N‐di‐Boc‐protected bisacridone, similar to our previously synthesized linear acridone systems.^10^ The O−Boc‐substituted bisacene **14 a** was isolated starting from **13** (49 % yield). This is counterintuitive as the valence structure of the respective N,N‐di−Boc‐species suggests increased stability due to an additional Clar sextet. DFT calculations show that **14 a** is more stable by 24.5 kcal mol^−1^ than the N−Boc‐derivative (see Table S3).

**9** was transformed into acridinoacridine **3** in 24 % yield by addition of lithiated TIPS‐acetylene followed by *in situ* methylation and subsequent deprotection with concomitant elimination of methanol (Scheme 1).^[10]^ As **14 a** was unreactive, **14 b** was synthesized from **13** with freshly distilled triflic anhydride, also resulting in aromatization. Stille coupling with triisopropylsilylethynyl stannane gave **4** in 51 % yield. All reported bisacenes are stable in air and can be stored at 8 °C over long periods of time. They are moderately soluble in common solvents (<1 mg mL^−1^). For stability in solution see Figure S5 in the Supporting Information.

**Figure 2:**
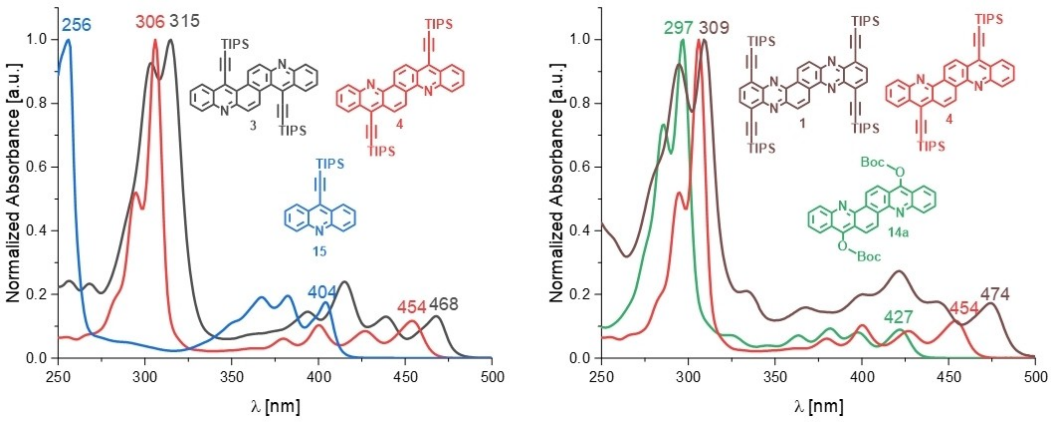
Normalized absorption spectra in *n*‐hexane. Left: **3**, **4** and model compound **15**. Right: **1**, **4** and **14 a**.

The absorption features of the azaacenoacenes are red‐shifted by approximately 50 nm compared to that of TIPS‐ethynylated acridine **15** (Figure 2), a reference compound (see Supporting Information). The absorption maximum of **3** is most red‐shifted (*λ*_max_=468 nm), 14 nm further than that of **4** (*λ*_max_=454 nm). This is backed by quantum‐chemical calculations of differences in HOMO LUMO gaps (15 nm, 0.08 eV, see Table S2, Figure S6). *In silico*, **3** adopts a twisted conformation as a consequence of the two fjord‐type regions and to avoid steric repulsion. This twist is also observed in the crystalline state (see below) – we assume that it is responsible for the decrease in the optical gap, an effect previously described by Gidron et al. for substituted anthracenes.^[13]^ This effect on the gap was reproduced *in silico* for a twisted, unsubstituted and nitrogen‐free derivative of **3** compared to its planar form. Its twist (29.8° end‐to‐end twist^[16]^) decreased the gap by 0.15 eV compared to the same molecule in planar geometry (Table S2, Figure S6). Unsubstituted planar model regioisomers of **3** and **4** do not significantly differ in their frontier molecular orbital (FMO) energy levels (see Supporting Information). Thus, the regioisomerism does not significantly alter the optoelectronic properties. When comparing **3** and **4** to **1**, the difference in substituents/nitrogen content barely influences the strongest absorption maximum (*λ*=309 nm for **1**) – the effects of additional nitrogen atoms in the backbone and additional ethynyl‐substitution nearly cancel each other out. *λ*_max_ (474 nm) of **1** is red shifted by 6 nm compared to that of **3** and 20 nm compared to that of **4**. The normalized absorption of **1** is generally broader and more intense over the whole absorption compared to **3** and **4**.

**Figure 3:**
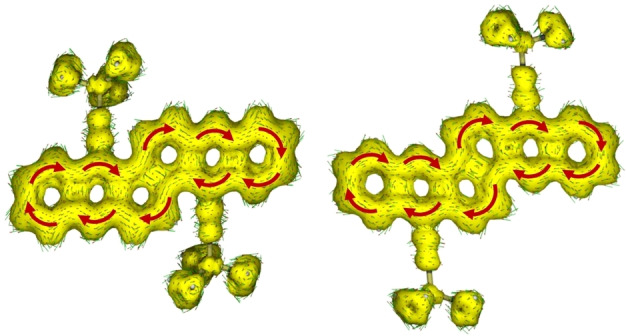
AICD^[14]^ plot of **3** (left) and **4** (right). The ring current at the edge of the formal acridines is interrupted; isovalue: 0.02; optimization limit: 0.02; maximal arrow length: 1; magnetic field vector is oriented out of plane.

Their anisotropy of the induced current density (AICD)^[14]^ plots display global diatropic (aromatic) ring currents – the shared bonds of the fused acridines are not included (Figure 3). As indicated by nucleus‐independent chemical shift (NICS(1)) values^[15]^ (see Figure S7), the central naphthalene unit in **3** and **4** displays decreased chemical shifts compared to the non‐fused outer rings of **15**. Overall, **3** is more aromatic than **4** as NICS(1) values in the latter are decreased by ca. 0.5 ppm per ring throughout the aromatic system. This effect was reproduced *in silico* for unsubstituted derivatives of **3**, attributing this change in aromaticity to the twist alone. This indicates that aromaticity can be tuned by twisting (bis‐)acenes.

The absorption spectra of **14 a** and **14 b** are almost identical (Figure S2). Compared to **4**, their absorption maxima at the longest wavelength (**14 a**: *λ*_max_=421 nm; **14 b**: *λ*_max_=427 nm) are blue‐shifted by 33 and 27 nm, respectively, attributed to the smaller π‐system due to the missing ethynyl substituents. All reported bisacenes show a yellow fluorescence with a small Stokes shift of 6 nm to 9 nm (see Table 1 and Supporting Information Table S1). Although the emission profiles appear similar, **15** exhibits a larger Stokes shift of 27 nm. The low solubility of the bisacenes prevented characterization by cyclic voltammetry.

**Table 1:** Optical, electrochemical and quantum‐chemical data.

Comp	*λ*_max, abs_^[a]^ [nm]	*λ*_onset, abs_^[a]^ [nm]	Opt. gap^[b]^ [eV]	*λ*_em_^[c]^ [nm]	HOMO^[d]^ [eV]	LUMO^[d]^ [eV]
**1** ^7^	474	488	2.54	505^[e]^	−6.01	−3.35
**3**	468	479	2.59	474	−5.67	−2.89
**4**	454	466	2.66	463	−5.67	−2.78
**14 a**	421	433	2.86	430	−5.91	−2.76
**14 b**	427	440	2.82	436	−6.19	−3.09
**15**	404	413	3.00	431	−5.90	−2.60

[a] Absorption measurements were performed in *n*‐hexane. *λ*_max,abs_ denotes the local absorption maximum at the longest wavelength. [b] Calculated from *λ*_onset,abs_. [c] Emission measurements of **3**, **4**, **15** were performed in *n*‐hexane. [d] Calculated from TMS‐ethynylated (methyl‐carbonate for **14 a**) derivatives using Gaussian 16 and B3LYP/def2‐TZVP level of theory.^[12]^ [e] Taken from ref. [5], measured in DCM.

**Figure 4:**
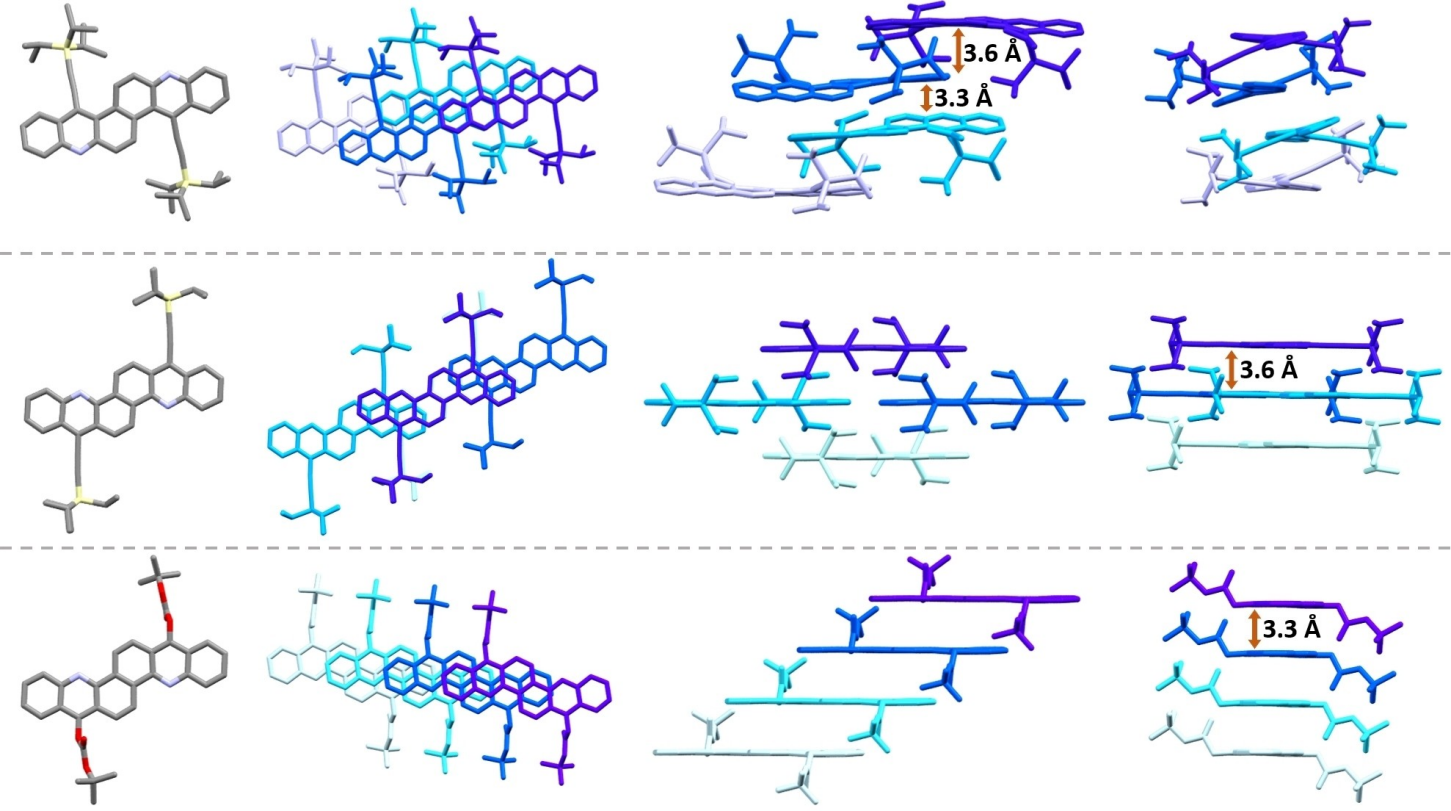
Solid‐state structure and packing of **3** (top), **4** (center) and **14 a** (bottom).

The solid‐state packing of the new bisacenes was determined by X‐ray crystallography (Figure 4, Figures S8–S10). **3** is twisted (25.3° end‐to‐end twist^[16]^). It packs in an A_2_B_2_ brick wall motif; resulting in π–π‐overlap between layers (Figure 4, top) with distances between 3.3–3.6 Å. Planar **4** packs in a brick wall arrangement. π–π‐distances of 3.58 Å indicate interaction of the aromatic backbones. The crystal packing of **4** is temperature‐dependent, two similar polymorphs of **4** exist at room temperature and −78 °C, respectively (Figure S10). According to grazing incidence diffraction, thin‐films of **3** and **4** display the same packing on surfaces used for device fabriacteion (see below) as observed for single crystals and have their molecular axis oriented orthogonally to the surface (Figure S13). The flexible substituents in **14 a** and **14 b** allow for a tighter packing and thus more pronounced π–π interactions. Compound **14 a** exhibits a herring‐bone packing motif (π–π distance: 3.32 Å). **14 b** packs in a 1D slipped stack (π–π distance: 3.34 Å, see Figure S12). **1**, bearing four TIPS‐ethynyl substituents, shows no π–π overlap (CCDC: 1946373),^[7]^ underlining the importance number and placement of the substituents on the morphology – see Supporting Information (Figure S14) for a Hirshfeld analysis^[17]^ comparing short contacts of **1**, **3** and **4**.

**Figure 5:**
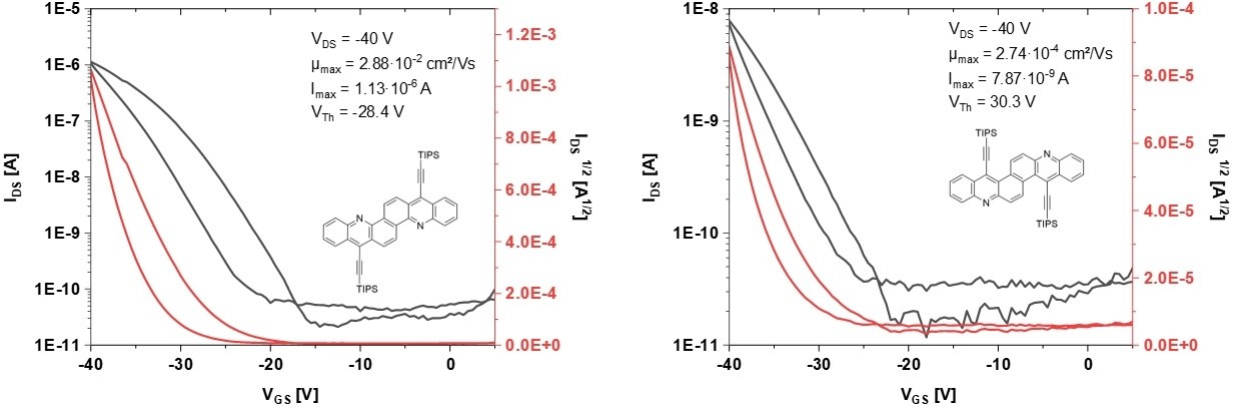
Transfer curves of the best performing OFET devices (**4**, left and **3**, right).

Bottom gate/top contact OFEts (Figure S16) with gold as electrode material and a bilayer dielectric consisting of SiO_2_ and Al_*x*_O_*y*_ coated with an alkyl‐SAM to prevent trap states were fabricated using **3** and **4**.^[18]^ **14 a**,**b** were difficult to process due to their low solubility. The best performing devices resulted from drop‐cast thin‐films of **4** (toluene/acetone 80 : 20, *c*=0.25 mg/mL). Values extracted from transfer measurements of 55 channels measured over 6 different wafers gave average hole mobilities of 5.3×10^−3^ cm^2^(Vs) ^−1^ and maximum saturation hole mobilities of up to 2.9×10^−2^ cm^2^(Vs)^−1^ and an on/off‐ratio of 10^4^ (Figure 5, left).

With SiO_2_ as dielectric, devices using **3** with maximum saturation hole mobilities of 2.7×10^−4^ cm^2^(Vs)^−1^ and an on/off‐ratio of 10^2^ could be fabricated (Figure 5, right). Pictures of the resulting thin‐films are provided in the Supporting Information. Measurements of TIPS‐pentacene^19^ as reference were carried out under the same conditions. For SAM‐coated substrates, peak mobilities were 0.58 cm^2^(Vs)^−1^ and for neat SiO_2_ substrates 2.8×10^−2^ cm^2^ (Vs)^−1^. **3** and **4** are reasonable p‐channel transport materials. Particularly **4** reaches a tenth of the mobility of the reference material TIPS‐pentacene extracted in our experiments. Calculated mobilities (see Section 9 of the Supporting Information) suggest that higher mobilities could be achieved with better film morphology.

## Conclusion

In conclusion, four novel aza‐acenoacenes **3**, **4**, and **14 a**,**b** were prepared. Their optoelectronic properties do not vastly differ when compared to **1**, although solid state packing is dramatically influenced by number and position of the TIPS‐ethynyl substituents. As a consequence, favorable π–π interactions result for the newly synthesized azabisacenes. OTFT devices of **4** performed with peak transfer mobilities of 2.9×10^−2^ cm^2^ (Vs)^−1^. This is one tenth the mobilities reached for single crystal devices of **2 a** and of the reference material TIPS‐pentacene that was fabricated under similar conditions. Using **3**, mobilities up to 2.7×10^−4^ cm^2^ (Vs) ^−1^ were obtained. This opens up the field of azaacenoacenes as promising semiconductor materials.

## Experimental Section

Deposition Number(s) 2155728 (**3**), 2155729 (**4** at rt), 2155730 (**4** at 200 K), 2155731 (**9**), 2155732 (**14 a**), 2155733 (**14 b**), 2155734 (**S1**), 2155735 (**S2**) contain(s) the supplementary crystallographic data for this paper. These data are provided free of charge by the joint Cambridge Crystallographic Data Centre and Fachinformationszentrum Karlsruhe Access Structures service.

The Supporting Information comprise details concerning synthesis, calculations and characterisation. Raw data related to synthesis and characterization of the reported compounds as well as transistor data are available through heiDATA, the institutional research data repository of Heidelberg University, under [https://doi.org/10.11588/data/645DDB].

**Procedure towards 5,13‐bis((triisopropylsilyl)ethynyl)acridino[2,1‐a]acridine 3**: In an oven‐dried Schlenk tube under argon atmosphere, 2.5 M *n*‐BuLi in *n*‐hexane (327 mg, 2.05 mL, 5.12 mmol, 8.00 equiv.) was slowly added to a solution of TIPS‐acetylene (1.05 g, 1.29 mL, 5.76 mmol, 9.00 equiv.) in dry THF at −78 °C. After stirring for 1 h, **9** (360 mg, 640 μmol, 1.00 equiv.) was added to the solution and the reaction was allowed to stir overnight while thawing. The reaction was subsequently quenched with methyl iodide (1.82 g, 796 μL, 12.8 mmol, 20.0 equiv.) and stirred for 8 h. 2 mL of saturated aqueous NH_4_Cl solution was added and the layers were separated. After concentrating in vacuo, the crude product was redissolved in DCM and washed with water, dried over Na_2_SO_4_ and evaporated under reduced pressure. The solid was then dissolved in little DCM and TFA (2.48 g, 1.67 mL, 25.6 mmol, 40.0 equiv.) was added to the stirring mixture at 0 °C. After stirring for 30 min an excess of saturated aqueous NaHCO_3_ solution was added and stirred for 5 min. The layers were separated and the organic phase was dried over Na_2_SO_4_ and evaporated under reduced pressure. The crude product was purified using silica gel column chromatography. The column was flushed with petrol ether before increasing the polarity to PE:EE 95 : 5. The product is obtained as a yellow solid (108 mg, 156 μmol, 24 %). For use in OFET, the solid was recrystallized three times by layering HPLC‐grade methanol, ethyl acetate and *n‐*hexane, respectively, over a solution of **3** in HPLC‐grade DCM. ^1^H NMR (600 MHz, CDCl_3_) *δ* 10.73 (d, *J*=9.5 Hz, 2H), 8.84–8.81 (m, 2H), 8.35–8.24 (m, 4H), 7.90–7.86 (m, 2H), 7.74–7.70 (m, 2H), 1.43–1.35 (m, 6H), 1.30 (d, *J*=7.2 Hz, 36H) ppm. ^13^C NMR (151 MHz, CDCl_3_) *δ* 148.81, 147.79, 130.78, 130.15, 130.09, 129.60, 128.62, 128.23, 127.39, 127.20, 126.63, 123.76, 113.09, 104.26, 19.01, 11.65 ppm. IR (neat) *ṽ*=2934, 2861, 2132, 1525, 1457, 1008, 879, 757, 668, 505 cm^−1^. HR‐MS(MALDI+): *m*/*z* calcd. for C_46_H_54_N_2_Si_2_: 690.3826; found 691.3897 [M+H]^+^. M.p.: 270–280 °C (dec.). Elemental analysis [%]=calcd. for C_46_H_54_N_2_Si_2_: C 79.94, H 7.88, N 4.05, Si 8.13; found: C 79.98, H 7.73, N 3.75.

## Conflict of interest

The authors declare no conflict of interest.

1

## Supporting information

As a service to our authors and readers, this journal provides supporting information supplied by the authors. Such materials are peer reviewed and may be re‐organized for online delivery, but are not copy‐edited or typeset. Technical support issues arising from supporting information (other than missing files) should be addressed to the authors.

Supporting Information

## Data Availability

Data are published in “HeiData”, the data repository from Heidelberg University ‐ the DOI is given in the manuscript.
