# Supplementary material for: N‐Acenoacenes: Synthesis and Solid‐State Properties
Source: Chemistry. 2022 Oct 25;28(69):e202201916. doi: 10.1002/chem.202201916 (PMC10091707; doi:10.1002/chem.202201916)
Supplement: Supplementary file 1 — Supporting Information [file CHEM-28-0-s001.pdf]

Chemistry–A European Journal

Supporting Information

# N-Acenoacenes: Synthesis and Solid-State Properties

Thomas Wiesner, Marcel Pardon, Steffen Maier, Frank Rominger, Jan Freudenberg,* and Uwe H. F. Bunz*

# Table of Contents


1. Optoelectronic properties ..... 2
   - i. Overview ..... 2
   - ii. Absorption and emission spectra ..... 2
   - iii. Extinction coefficients ..... 3
   - iv. Stability study ..... 4
2. *In-silico* study of constitutional effects ..... 5
3. NICS(1) calculations ..... 6
4. AICD Plots ..... 7
5. Crystal structures ..... 8
   - i. Polymorph-comparison for 4 ..... 8
   - ii. Crystal structure of 14a ..... 8
   - iii. Crystal structure of 14b ..... 9
6. Grazing incidence diffractometry ..... 9
7. Hirshfeld analysis ..... 10
8. Devices ..... 11
9. Transfer integrals ..... 13
10. Synthesis ..... 15
11. NMR spectra ..... 22
12. Crystallographic data ..... 36

# 1. Optoelectronic properties

## i. Overview

Table S1. Optical, electrochemical and quantum-chemical data of compounds **1**-**3**.

| # | Comp. | $\lambda_{max}$ [nm][a] | $\lambda_{onset}$ [nm][a] | Opt. gap [eV][b] | $\lambda_{Em}$ [nm][c] | HOMO$_{DFT}$[d] [eV] | LUMO$_{DFT}$[d] [eV] | $\Delta E_{DFT}$ [eV] | $\Delta E_{DFT}$ [nm] |
|---|---|---|---|---|---|---|---|---|---|
| 1 | **1**[1] | 474 | 488 | 2.54 | 505[e] | -6.01 | -3.35 | 2.66 | 466 |
| 2 | **3** | 468 | 479 | 2.59 | 474 | -5.67 | -2.89 | 2.78 | 445 |
| 3 | **4** | 454 | 466 | 2.66 | 463 | -5.67 | -2.78 | 2.89 | 430 |
| 4 | **14a** | 421 | 433 | 2.86 | 430 | -5.91 | -2.76 | 3.15 | 393 |
| 5 | **14b** | 427 | 440 | 2.82 | 436 | -6.19 | -3.09 | 3.11 | 399 |
| 6 | **15** | 404 | 413 | 3.00 | 431 | -5.90 | -2.60 | 3.31 | 375 |

[a] Absorption measurements were performed in *n*-hexane. $\lambda_{max}$ denotes the local absorption maximum at the longest wavelength. [b] Calculated from $\lambda_{onset}$ [c] Emission of **3**, **4**, **15**, were measured n *n*-Hexane. [d] Calc. for TMS-ethynylated derivatives on the B3LYP/def2-TZVP-level. [e] Taken from ref. 1.

## ii. Absorption and emission spectra

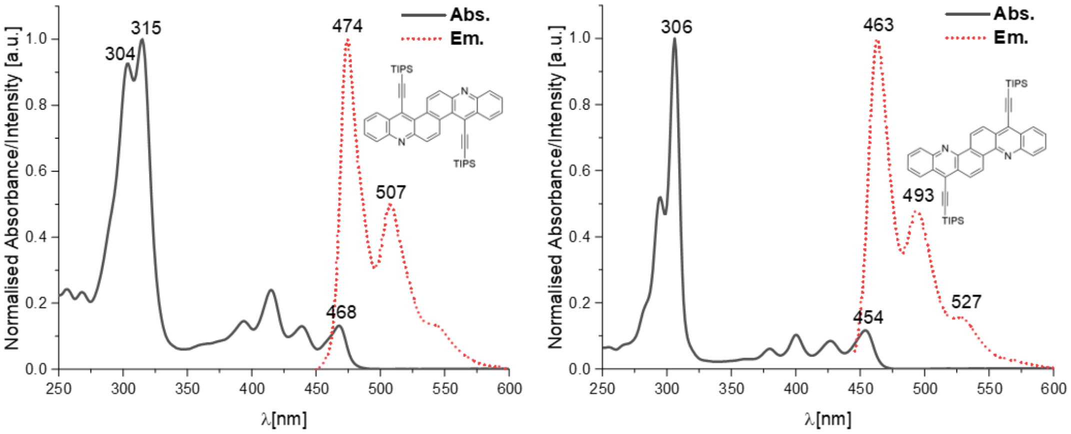


Figure S1. Absorption and emission spectra for **3** (left) and **4** (right) in *n*-hexane

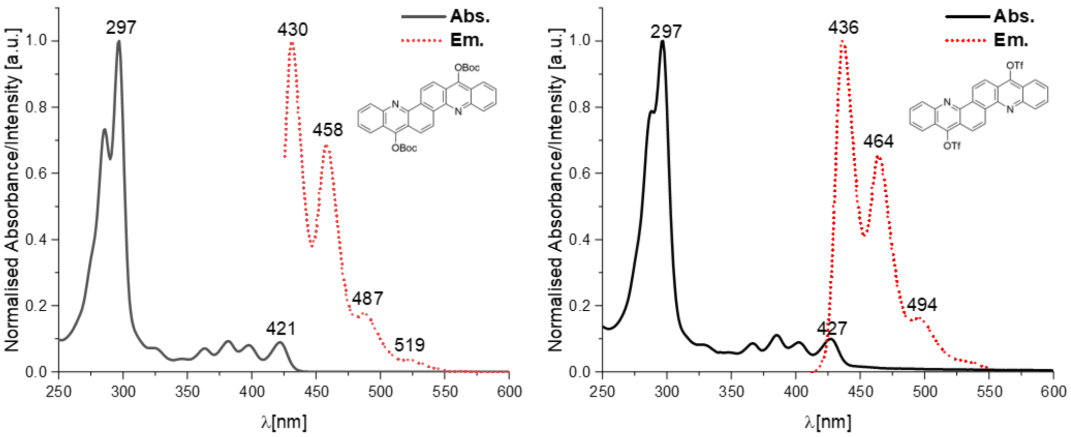


Figure S2. Absorption and emission spectra for **14a** (left) and **14b** (right) in *n*-hexane.

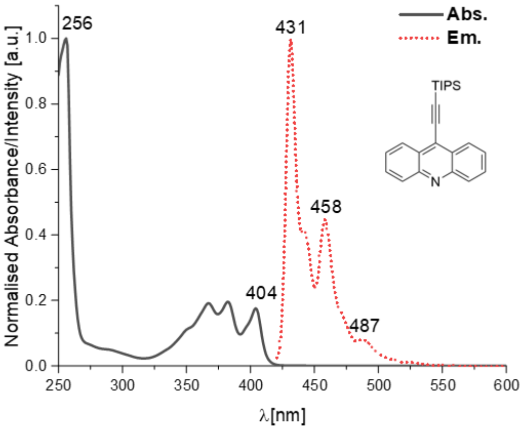


Figure S3. Absorption and emission spectrum for **2** in *n*-hexane.

## iii. Extinction coefficients

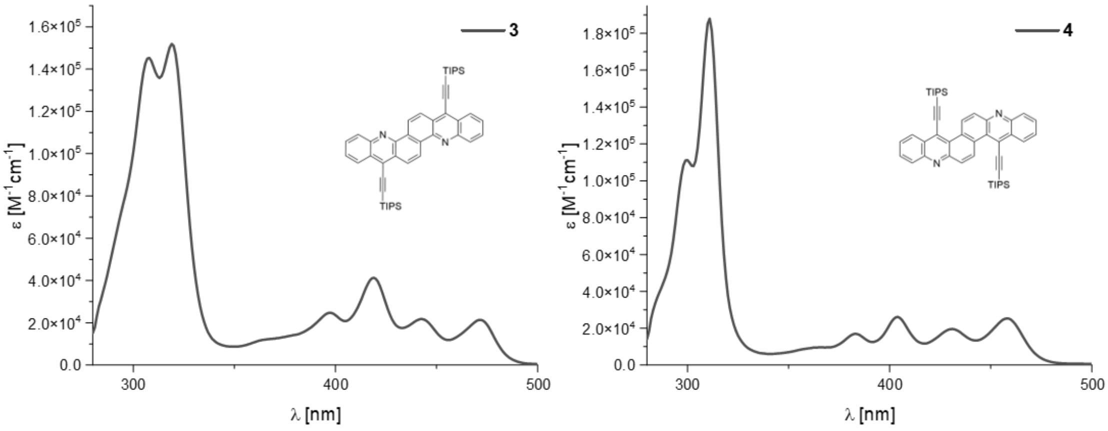


Figure S4. Molar extinction coefficients for compounds **3** and **4** in toluene.

## iv. Stability study

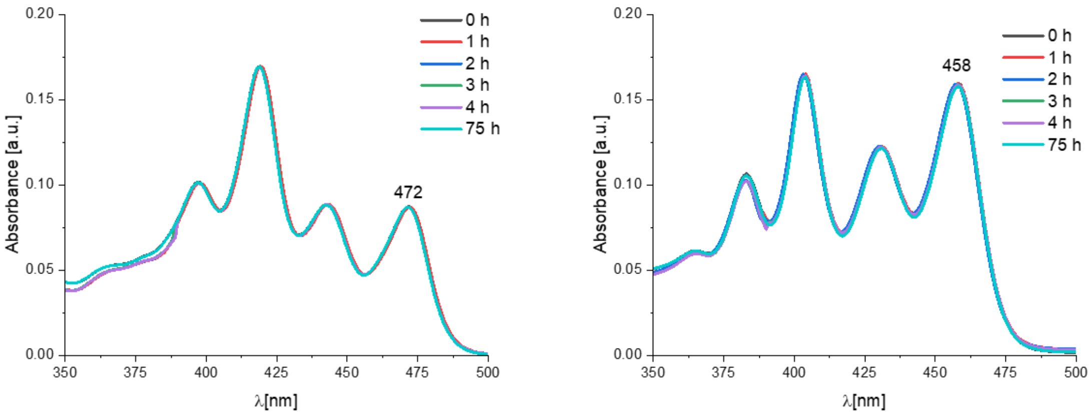


Figure S5. Stability of **3** (left) and **4** (right) in toluene under ambient conditions.

## 2. *In-silico* study of constitutional effects

Table S2. Calculated HOMO and LUMO energies for different derivatives of **3** und **4**.

| Compound[a] | | $E_{LUMO, DFT}$ [eV][b] | $E_{HOMO, DFT}$ [eV][b] | $\Delta E_{DFT}$ | |
|---|---|---|---|---|---|
| | | | | [eV] | [nm] |
| **3*** | 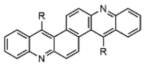 | -2.89 | -5.67 | 2.78 | 446 |
| **4** | 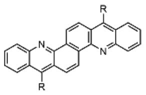 | -2.78 | -5.67 | 2.89 | 429 |
| **3-unsub** | 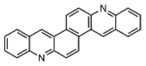 | -2.62 | -5.85 | 3.23 | 384 |
| **4-unsub** | 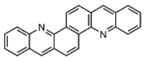 | -2.56 | -5.74 | 3.18 | 390 |
| **3-nonaza*** | 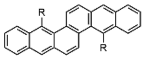 | -2.53 | -5.25 | 2.72 | 456 |
| **4-nonaza** | 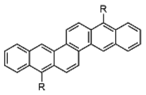 | -2.47 | -5.35 | 2.87 | 432 |
| **Unsub-nonaza** | 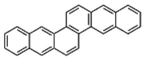 | -2.96 | -5.94 | 3.13 | 396 |

[a] R = TMS-Ethynyl. [b] Obtained from quantum-chemical DFT calculations (B3LYP/def2-TZVP). * Exhibited a twisted molecular structure in their optimized geometry.

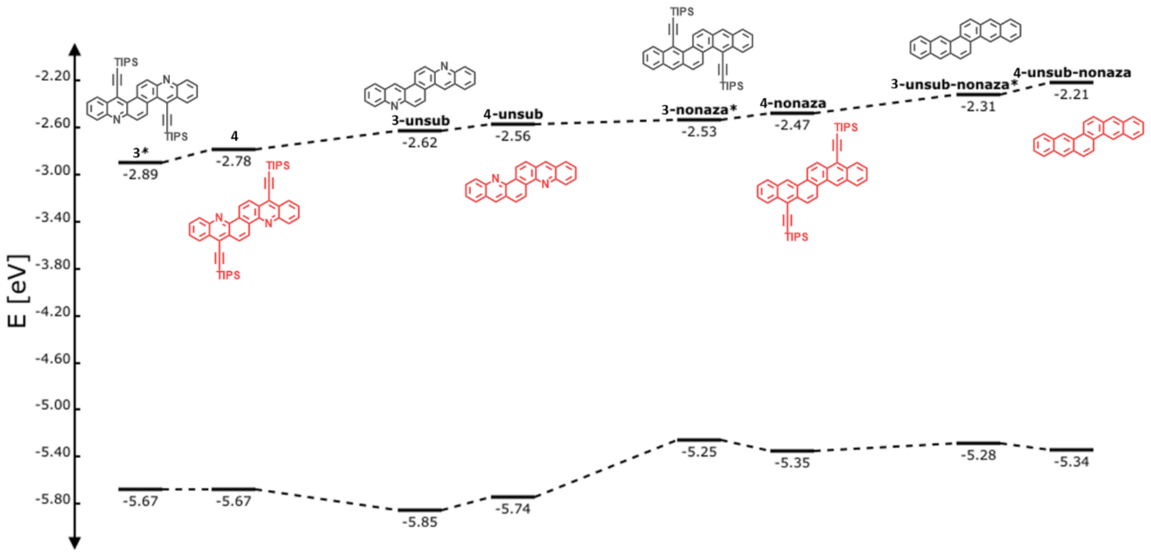


Figure S6. Visualization of constitutional effects using values from Table S1. Compounds with an asterisk exhibit a twist in their optimized geometry.

The visualization in Figure S6 shows that the twist leads to a narrowing of the HOMO-LUMO-Gap (compare 3-unsub-nonaza* and 4-unsub-nonaza). The introduction of TIPS-Ethynyl-groups leads to a lowering of the HOMO-Energy-level while not significantly altering that of the LUMO (3,4-nonaza vs. 3,4-unsub-nonaza). Planar Acenoacenes containing nitrogen are predicted to have lower HOMO- and LUMO energies compared to their nitrogen-free derivatives (4-unsub vs. 4-unsub-nonaza). A nitrogen-

distribution as in **3** leads to a lower HOMO-energy than that of **4** (3-unsub vs. 4-unsub). The effects add up to identical HOMO-energies and lower LUMO-energies when comparing **3*** and **4**.

Table S3. Frequency analysis of methyloxycarbonylated derivatives of **14a** and **14b** (B3LYP/def2TZVP).

| | 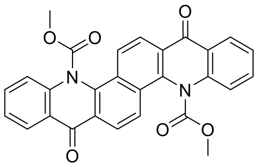 | 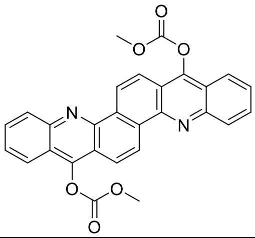 |
|---|---|---|
| HOMO | -6.10 | -2.63 |
| LUMO | -5.91 | -2.75 |
| Electronic energy + Thermal Free Enthalpy correction [kcal/mol] | -1028462.437 | -1028486.900 |

## 3. **NICS(1) calculations**

NICS(1)-values were calculated using the GIAO-NMR-method at the B3LYP/def2TZVP level of theory.

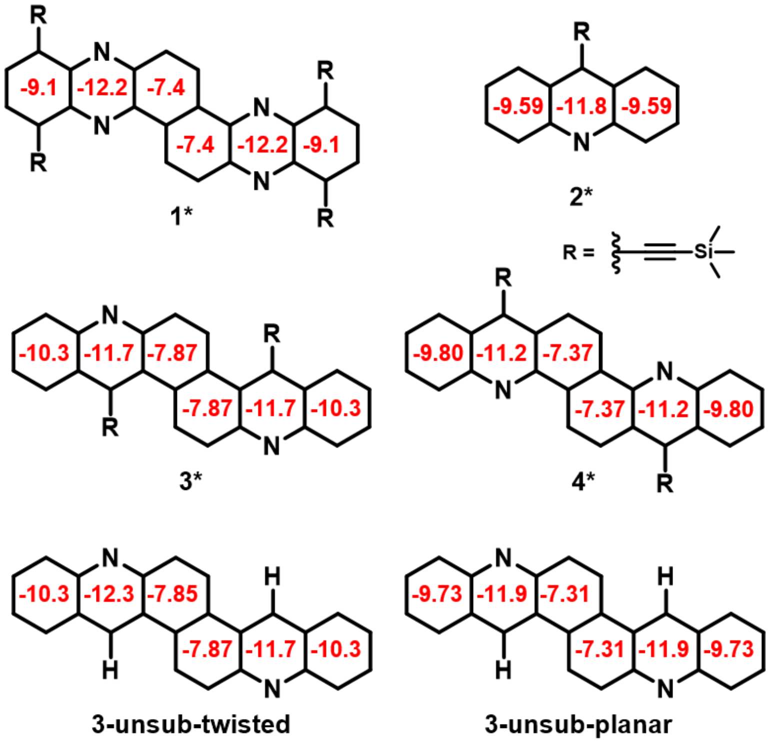


Figure S7. NICS(1) values for the reported compounds. Values for **1*** were taken from ref. 1. The asterisk indicates the replacement of TIPS by TMS to reduce recources needed for the calculations.

Figure S7 shows increased aromaticity when comparing **3** to **4**. Control experiments with unsubstituted planar or twisted derivatives of **3** reproduce this, indicating that this change aromaticity is not attributable to the regioisomerism, and that (global) aromaticity can be tuned by controlling the twist of acenes.

## 4. **AICD Plots**

AICD-plots were calculated using AICD-3.0.3 using the CSGT-method at the B3LYP/def2-TZVP IOP(10/93=1) level of theory; isovalue: 0.02; optimization limit: 0.02; maximal arrow length: 1; magnetic field vector is oriented out of plane.[2]

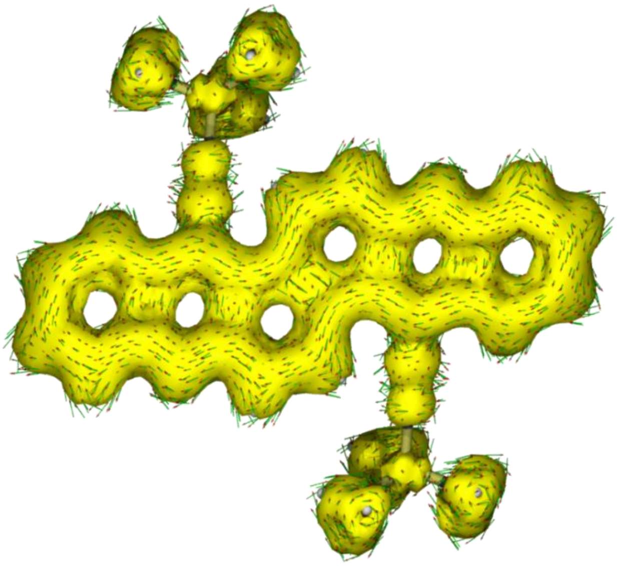

Figure S8. AICD-Plot of **3**.

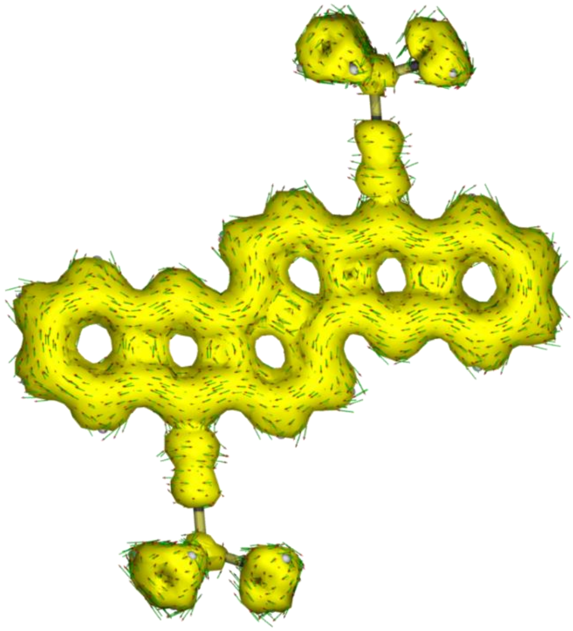

Figure S9. AICD-Plot of **4**.

## 5. **Crystal structures**

### i. Polymorph-comparison for 4

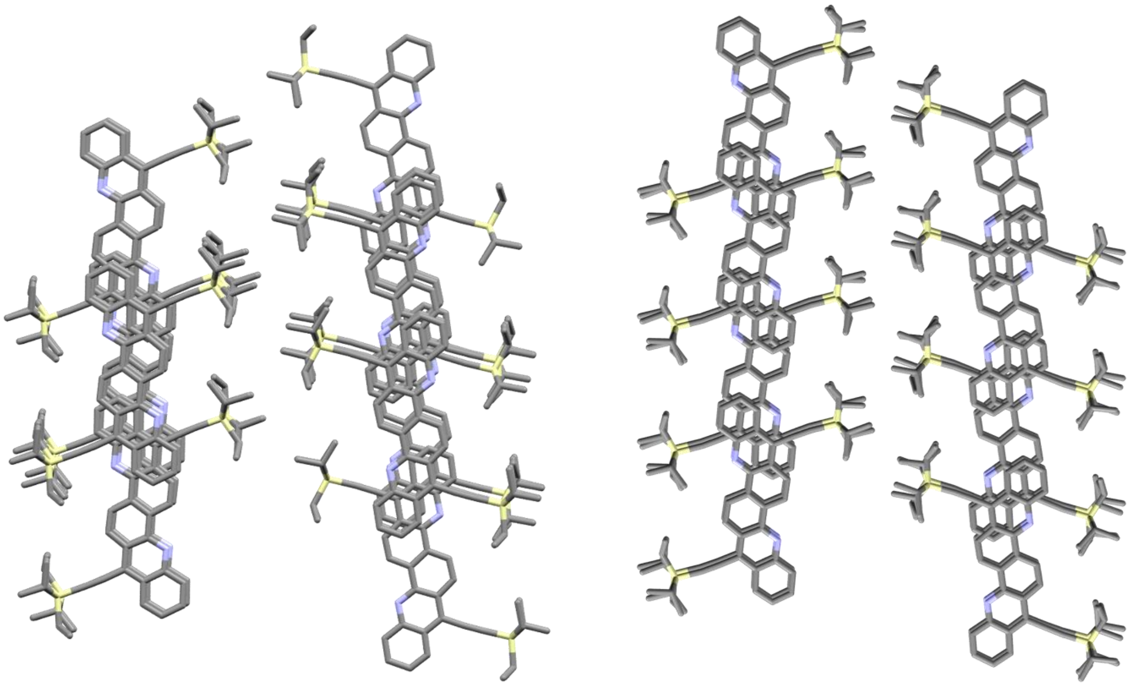

Figure S10. Polymorphs of **4** obtained for measurements at different temperatures, comparison of 2x2x2 unit cells. Left: Polymorph A (measured at rt); Right: Polymorph B (measured at -78 °C).

Polymorph B exhibits a slightly decreased π-π distance of 3.5 Å (Polymorph A: 3.58 Å).

### ii. Crystal structure of 14a

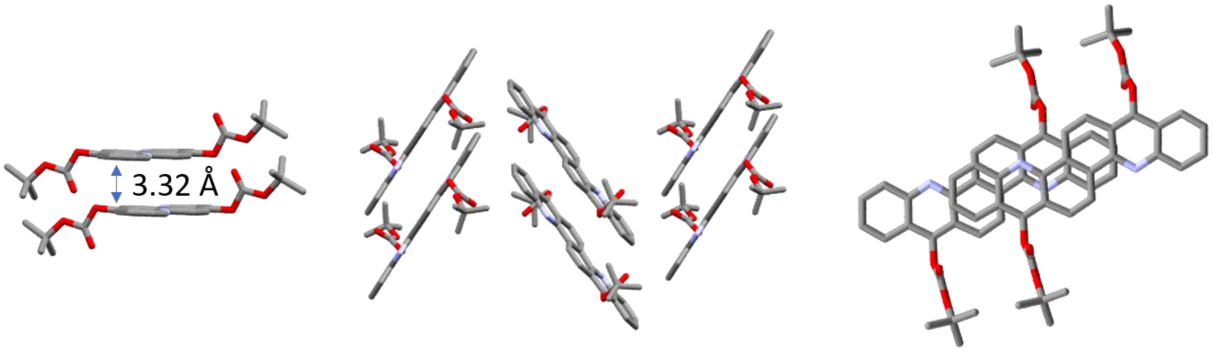


Figure S11. Solid-state packing obtained for **14a**. a) Front view, b) side view (herring-bone), c) top view.

### iii. Crystal structure of 14b

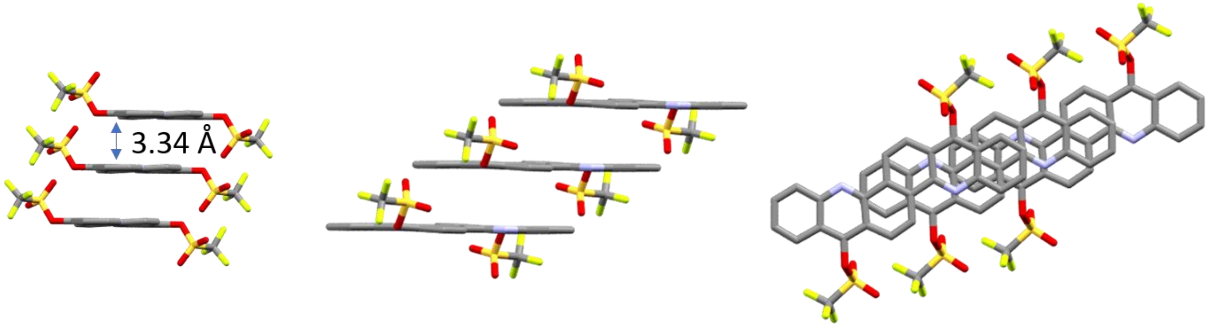


Figure S12. Solid-state packing obtained for **14b**. a) Front view, b) side view (1D-slipped stack), c) top view.

**14b** shows significantly higher overlap of π-planes. The π-π-distance is very similar (3.32 Å for **14a**; 3.34 Å for **14b**).

## 6. Grazing incidence diffractometry

Thin-films of **3** and **4** were investigated using X-ray diffraction to gain verify the packing mode on the surface and the orientation of the crystal planes with respect to the surface. The crystal plane that reflects the XRD is parallel to the surface. The comparison of the measurements with the corresponding simulated powder pattern are shown in Figure S13. Averaging the two angles given for **3** gives an angle of the effective molecular plane and the crystal lattice plane (100) of 88°.

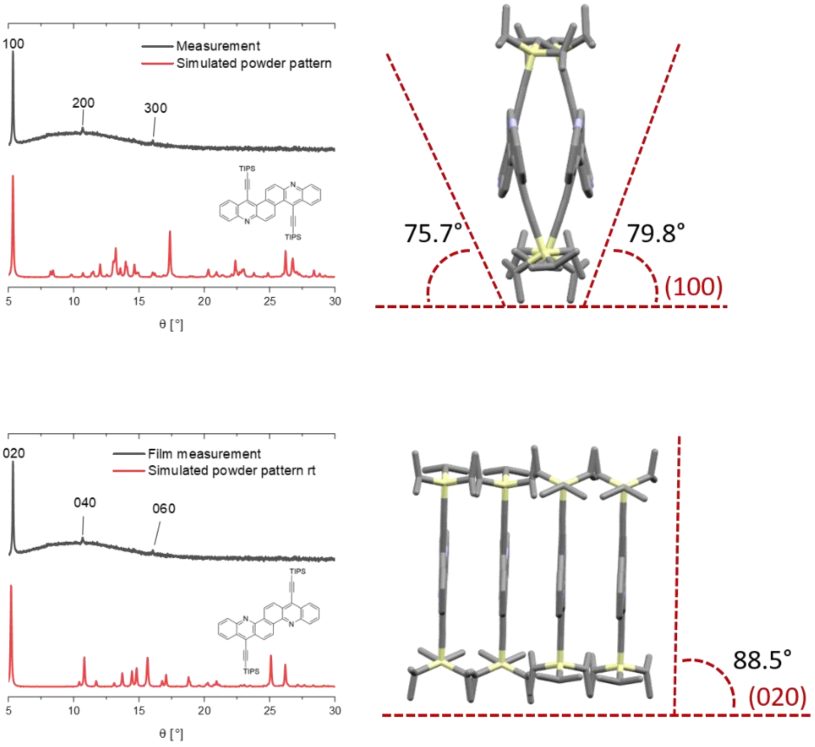


Figure S13. GID-Plots and Front-Views of solid-state packings of compound **3** (top) and **4** (bottom) viewed along the reflecting crystal lattice plane (001) and (020), respectively.

## 7. **Hirshfeld analysis**

Hirshfeld analysis was carried out using crystal explorer[3].

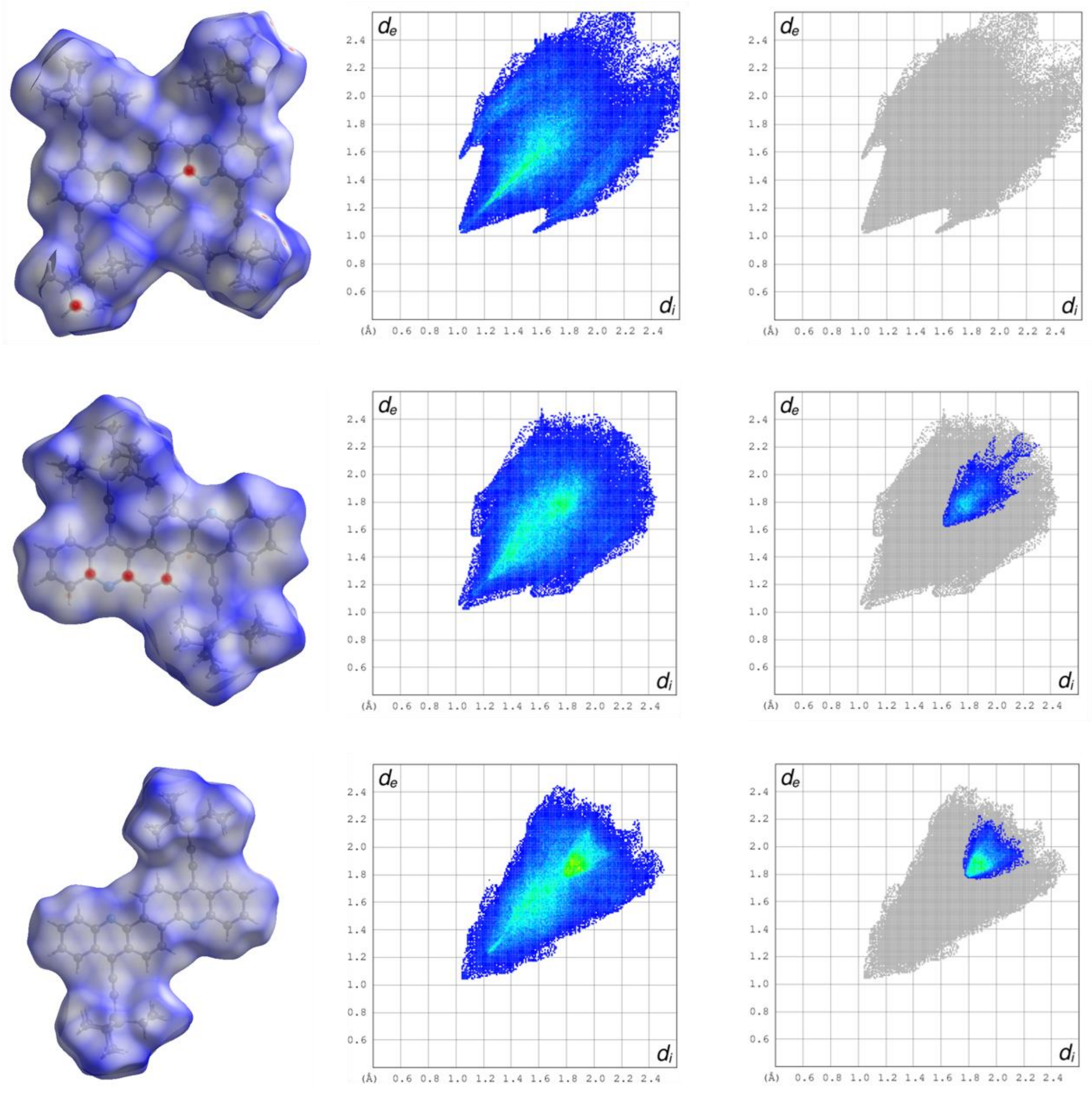


Figure S14. Hirshfeld-analysis for **1** (top), **3** (middle) and **4** (bottom). The left column shows a graphical depiction of the Hirshfeld surface. The middle column shows the fingerprint plot. The right column shows the fingerprint-plot filtered for C-C-distances.

The Hirshfeld plots show how the reduction of steric demand in **3** and **4** leads to a close packing. Twisted **3** shows a larger scattering in C-C distances than planar **4**.

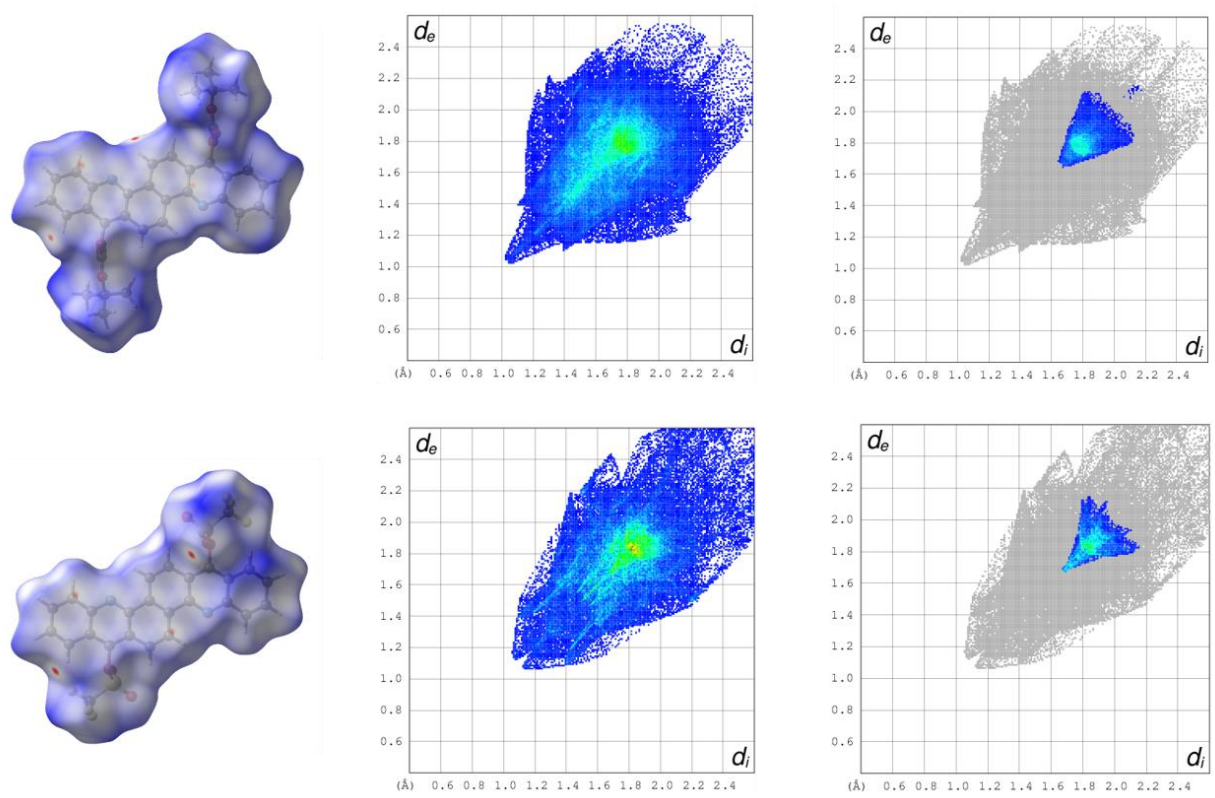


Figure S15. Hirshfeld-analysis for **14a** (top) and 1**4b** (bottom). The left column shows a graphical depiction of the Hirshfeld surface. The middle column shows the fingerprint plot. The right column shows the fingerprint-plot filtered for C-C-distances.

The reduced packing distance of **14a** and **14b** is reflected in the Hirshfeld plots filtered for C-C-distances (Figure S15).

# 8. Devices

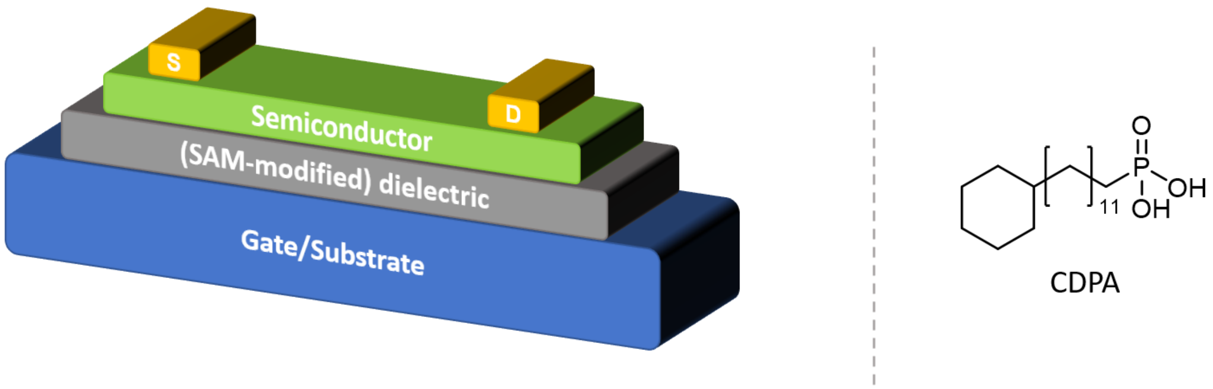


Figure S16. Device architecture used in the experiment (left); Structure of cyclohexyldodecylphosphonic acid (CDPA, right)

The surface was prepared as described in detail in ref. 4. Gold electrodes with a thickness of 30-40 nm were evaporated at a rate of 0.04 Å $s^{-1}$ at pressures lower than $10^{-6}$ bar through patterned shadow masks.

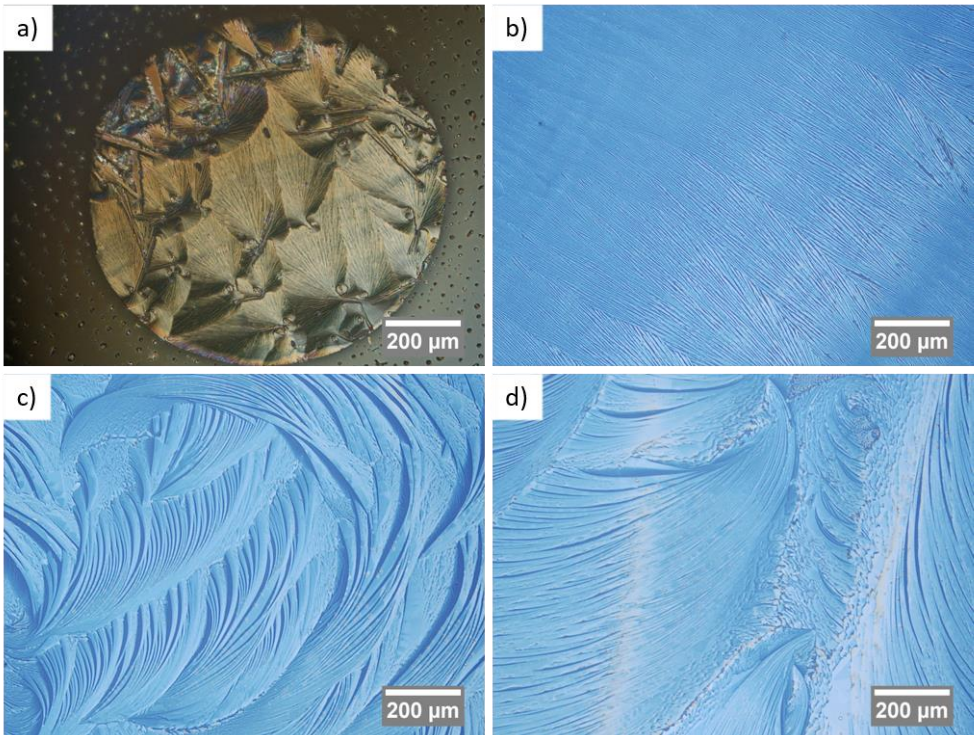


Figure S 17. Polarized light microscopy images of drop-cast films of **3** (10x magnification). a) **3** on a defect in a CDPA-coated surface. b) **3** on $SiO_2$ from DCM (0.25 mg $mL^{-1}$) at r.t. b) on $SiO_2$ from DCM (0.25 mg $mL^{-1}$) at r.t. c) **3** on $SiO_2$ from Toluene (0.25 mg $mL^{-1}$) at 50 °C. d) **3** on SiO2 from Toluene:Acetone 9:1 (0.5 mg $mL^{-1}$) at 50 °C.

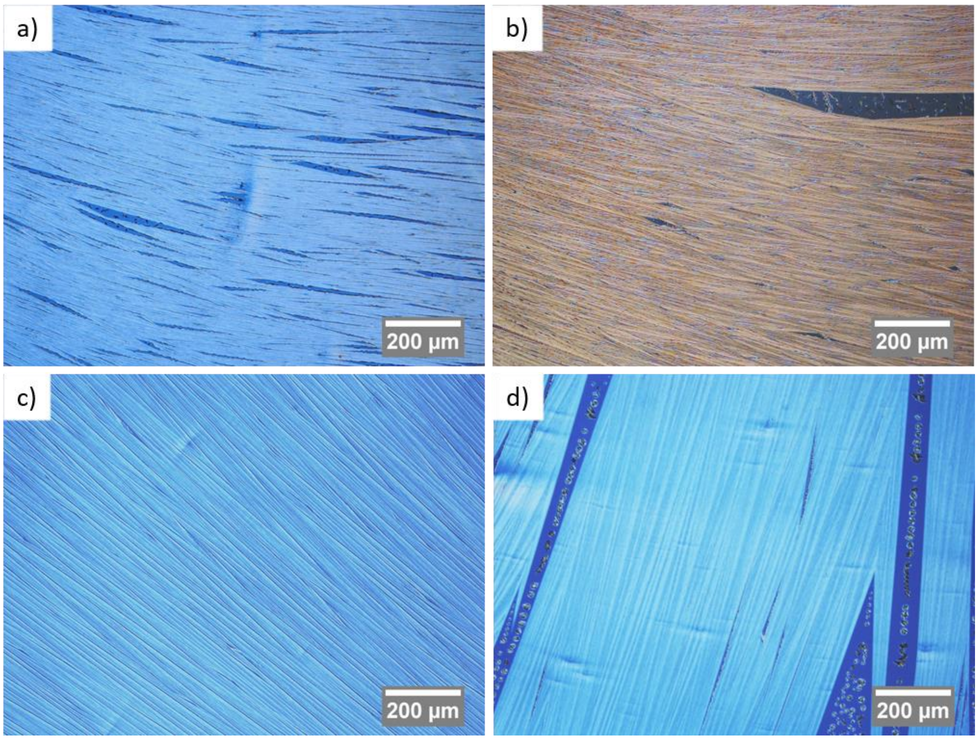


Figure S 18. Polarized light microscopy images of drop-cast films of **4** (10x magnification; 50 °C substrate temperature). a) **4** from Toluene (0.5 mg $mL^{-1}$) on CDPA. b) **4** from Toluene:Acetone 8:2 (0.25 mg $mL^{-1}$) on CDPA. c) **4** from Toluene (0.5 mg $mL^{-1}$) on oxygen-plasma activated $SiO_2$. d) **4** from Toluene:Acetone 9:1 (0.5 mg $mL^{-1}$) on oxygen-plasma activated $SiO_2$.

## 9. **Transfer integrals**

Transfer integrals were calculated using the ADF program. DZ Basis set and GGA PW91 as functional were used. The calculation was performed for each dimer pair of a crystal structure. The used dimers and the corresponding transfer integrals are shown below. Reorganization energies were calculated using the four-point method. Therefore, a geometry optimization of the isolated monomer in the gas phase was performed for the neutral ($E_{(M)}$) and the anionic species ($E^{-}_{(M-)}$) was performed. Afterwards, single point energy calculations were perfomed starting from the coordinates of the neutral specie and charge the molecule negative ($E_{(M-)}$) and starting from the coordinates of the anionic specie where the charge was set neutral ($E^{-}_{(M)}$).[5] A first geometry optimization was perfomed using Guassian 16 and the B3LYP/def2svp level of theory. A second Geometry optimization as well as single point calculations were performed using Gaussian 16 and the B3LYP/def2tzvp level of theory.

$$\lambda = \lambda_1 + \lambda_2$$

$$\lambda_1 = E_{(M-)} - E_{(M)}$$

$$\lambda_2 = E^{-}_{(M)} - E^{-}_{(M-)}$$

The reorganization energies and the transfer integrals were used to calculate the electron transfer rate using the Marcus theory.[6] The results for all possible transfer paths are summarized in the figures below.

$$k_{ET} = \frac{4\pi}{h}\frac{V^2}{\sqrt{4\pi k_b T}} e^{-\frac{\lambda}{4k_b T}}$$

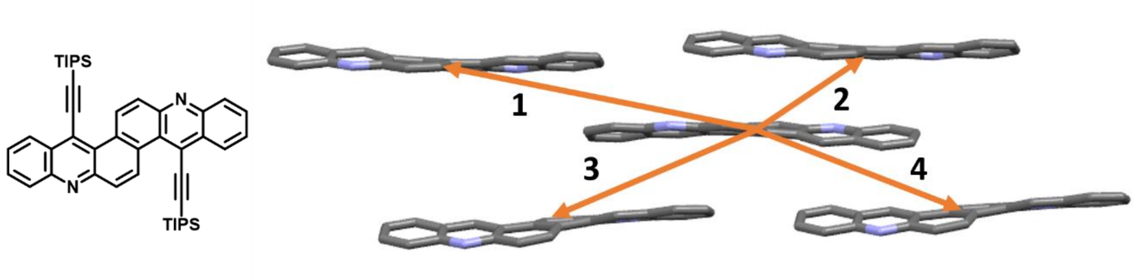


| Dimer Pair | Transfer Integral V (for e$^-$) [meV] | Reorganisation energy λ [meV] | Electron transfer rate $K_{ET}$ [1/s] |
|---|---|---|---|
| 1 | 2.90 | 142 | 1.19*10$^{11}$ |
| 2 | 2.69 | | 1.03*10$^{11}$ |
| 3 | 52.4 | | 3.90*10$^{13}$ |
| 4 | 2.69 | | 1.03*10$^{11}$ |

**Figure S1.** Top: Dimer Pairs of **3** used for the calculation of transfer integrals; Bottom: Calculated transfer integrals, reorganization energy and electron transfer rate.

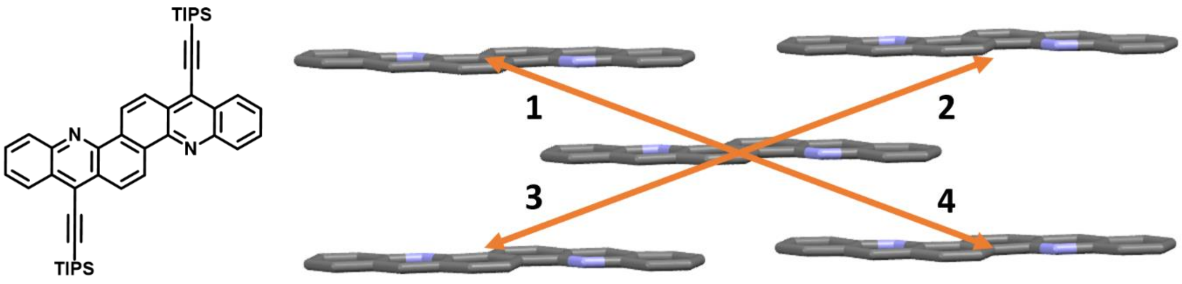


| Dimer Pair | Transfer Integral V (for e⁻) [meV] | Reorganisation energy λ [meV] | Electron transfer rate $K_{ET}$ [1/s] |
|---|---|---|---|
| 1 | 37.4 | 170 | $1.81*10^{13}$ |
| 2 | 34.2 | | $1.52*10^{13}$ |
| 3 | 35.3 | | $1.61*10^{13}$ |
| 4 | 34.2 | | $1.52*10^{13}$ |

**Figure S2.** Top: Dimer Pairs of **4** used for the calculation of transfer integrals; Bottom: Calculated transfer integrals, reorganization energy and electron transfer rate.

Afterwards the theoretical electron mobility was calculated assuming a charge carrier diffusion in the crystal. The diffusion coefficient D was calculated as follows:[7]

$$D \approx \frac{1}{2n}\sum_i r_i^2 k_{ETi} P_i$$

$$P_i = \frac{k_{ETi}}{\sum_i k_{ETi}}$$

n: Dimension; $r_i$: intermolecular distance between two molecules; $P_i$: Hopping probability for path i.

The theoretical electron mobility $\mu_{DFT}$ was calculated using the following formula[8]:

$$\mu_{DFT} = \frac{e}{k_b T} D$$

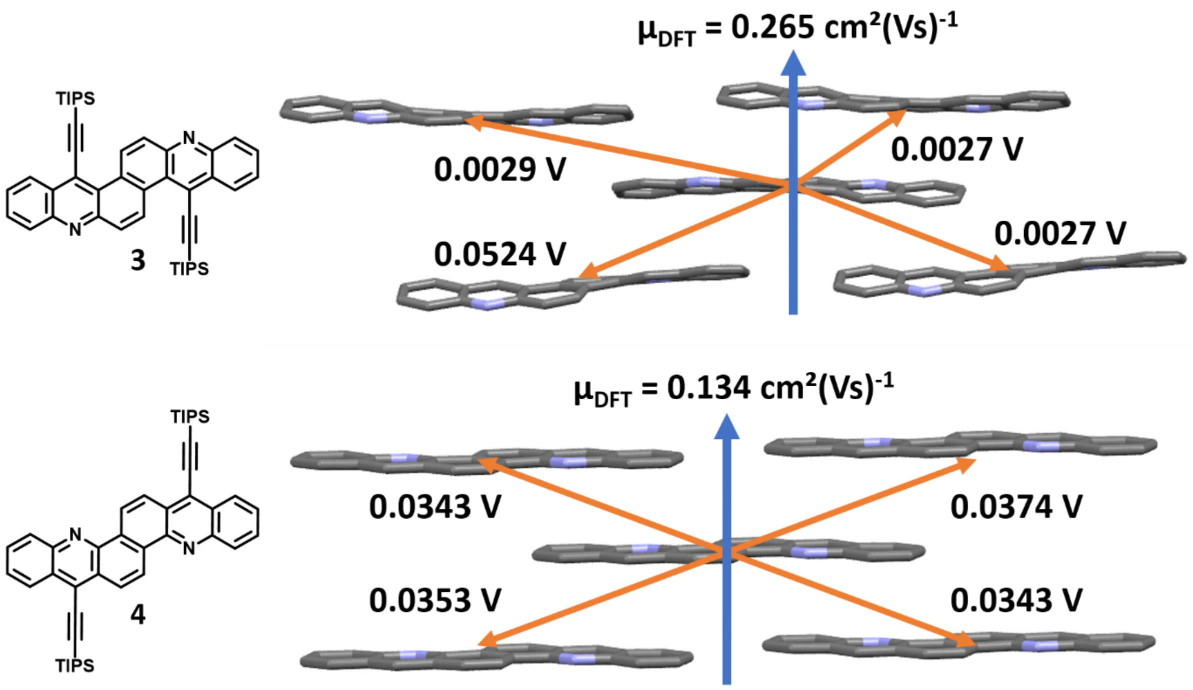


Figure S19. Calculated transfer integrals and mobilities for **3** and **4**.

The calculated transfer integrals and mobilities suggest that higher mobilities could be achieved i.e. in single crystal devices. The higher mobility of **3** can be attributed to the smaller distances between the crystal planes and the lower reorganization energy.

# 10.**Synthesis**

**General Remarks**

All commercially available chemicals and solvents were used without further purification. Silica gel (particle size: 0.032−0.062 mm) was obtained from Macherey, Nagel & Co. Absolute solvents were dried with a solvent purification system (MB SPS-800). Durapore® 0.1 µm PVDF membrane filters were purchased from MERK (Darmstadt, Germany). Eaton's reagent was prepared by dissolving $P_2O_5$ in methanesulfonic acid overnight (ca. 7% *w/w*). It was stored for months in a Schlenk tube under argon with a teflon stopcock without significant loss of reactivity.[9] NMR-Spectra were recorded using Bruker Avance spectrometers using the given frequency. Chemical shifts (δ) are given in parts per million (ppm) relative to respective solvent residue signals. IR spectra were recorded on a Jasco FT/IR-4100 spectrometer using neat solid samples. Absorption spectra were recorded using a Jasco UV-VIS V-670 spectrophotometer. Emission spectra were recorded using a Absorption spectra were recorded on a Jasco UV-VIS V-660 or V-670 Spectrophotometer. Fluorescence spectra were recorded on a Jasco FP-6500 Spectrofluorometer.

Computational studies were carried out using DFT calculations on Gaussian 16. Geometry optimizations were performed using the B3LYP functional and def-2-TZVP basis set. First, the gas-phase ground-state equilibrium geometry of the molecules was optimized at the B3LYP/def2-SVP level of theory. Afterwards the received geometries were refined using the B3LYP/def2-TZVP level of theory. FMO calculations were performed starting from the optimized geometries on the b3lyp/def2tzvp Level of theory.

**GP1** Buchwald Hartwig Amination

In a heat-gun-dried Schlenk tube under argon atmosphere, the bromide (1.00 eq.), the amine (2.00-4.00 eq.) and cesium carbonate (3.00-4.00 eq.) were added to dry toluene and the suspension was degassed for 30 min under a stream of argon. Then, a suitable palladium catalyst (4-5 mol%) was added and the reaction mixture was stirred at elevated temperatures. After workup, the product was either precipitated by evaporating DCM from a mixture of DCM and methanol or purified *via* silica-gel column chromatography.

**GP2** Saponification

The ester starting material (1.00 eq.) was dissolved in THF (hot THF if needed) and KOH (30.0 eq.) dissolved in a small amount of methanol was added to the reaction mixture. After stirring at room temperature overnight, the reaction mixture was concentrated under reduced pressure to roughly 1/5 of the initial volume and then diluted with deionized water. The product was precipitated from the resulting solution using 6 M HCl and filtered off using a PVDF membrane filter. The cake was washed with water, ethanol and DCM and dried in an oven at 80 °C. The product was used without further purification.

**GP3** Ring closure

In a heat-gun-dried Schlenk tube under argon atmosphere the starting material was dissolved in Eaton's reagent (2 mL reagent per 100 mg starting material). The resulting solution was stirred at 65 - 90 °C overnight before it was slowly pipetted into vigorously stirred deionized water. The suspension's pH was adjusted to 8 - 10 using KOH and $NaHCO_3$ and the product was filtered off using a PVDF membrane filter. The filter cake was washed thoroughly with water, methanol and DCM before it was dried in an oven at 80 °C. The product was used without further purification.

**GP4** Boc-protection

In a heat-gun-dried Schlenk tube under argon atmosphere the powdered acridone starting material (1.00 eq.), $Boc_2O$ (10.0 eq.), DMAP (1.00 eq.) and 2,6-Lutidine (5.00 eq.) were suspended in dry DCM or dry THF. The reaction mixture was stirred at rt - 50 °C overnight before it was diluted with methanol. The reaction solvent was removed from the mixture under reduced pressure and the suspension was filtered. The cake was washed with methanol, dissolved in DCM diluted with methanol precipitated by removing the DCM under reduced pressure. After filtration and washing with methanol the crude product was filtered over a silica pad and eluted with DCM if still impure.

**GP5** Addition of alkylsilylacetylene and deprotection of Boc-groups

In an oven-dried Schlenk tube under argon atmosphere, 2.5 M *n*-BuLi in *n*-hexane (4.00 eq.) was slowly added to a solution of the employed silylylated acetylene (5.00 eq.) in dry THF at -78 °C. After stirring for 1 h, the boc-protected quinacridone (1.00 eq.) was added to the solution and the reaction was allowed to stir overnight while thawing. The reaction was subsequently quenched with methyl iodide (10.0 eq.) and stirred for 8 h. A small volume of saturated aqueous $NH_4Cl$ solution was added and the layers were separated. After concentrating in vacuo, the crude product was redissolved in DCM and washed with water, dried over $Na_2SO_4$ and evaporated under reduced pressure. The solid was then dissolved in little DCM and TFA (40.0 eq.) was added to the stirring mixture at 0 °C. After stirring for 10 - 30 min an excess of saturated aqueous $NaHCO_3$ solution was added and stirred until the bright green solution turned dark. The layers were separated and the organic phase was dried over $Na_2SO_4$ and evaporated under reduced pressure. The crude product was purified using chromatography on

silica. The column was flushed with petrol ether before increasing the polarity to the desired eluent to remove unreacted silyl-acetylene.

## Dimethyl 2,2'-(naphthalene-2,6-diylbis(azanediyl))dibenzoate **6**

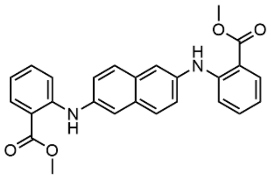

Compound **6** was synthesized according to **GP1** from 2,6-dibromonaphthalene **5** (1.00 g, 3.50 mmol, 1.00 eq.), methylanthranilate (1.32 g, 1.30 mL, 8.74 mmol, 2.50 eq.), cesium carbonate (3.99 g, 12.24 mmol, 3.50 eq.) and RuPhos-Pd G1 (142 mg, 175 µmol, 5 mol%). The reaction mixture was stirred at 120 °C overnight and was directly poured on a silica gel column packed with toluene and eluted with toluene. The pure product was obtained as a beige solid (1.35 g, 3.17 mmol, 91%).

$^{1}$H NMR (400 MHz, $CDCl_3$) *δ* 9.62 (s, 2H), 8.01 – 7.08 (m, 2H), 7.73 – 7.70 (m, 2H), 7.64 – 7.63 (m, 2H), 7.39 – 7.35 (m, 2H), 7.35 – 7.32 (m, 4H), 6.79 – 6.74 (m, 2H), 3.93 (s, 6H) ppm. $^{13}$C NMR (101 MHz, $CDCl_3$) *δ* 169.11, 148.16, 137.53, 134.30, 131.82, 131.45, 128.39, 123.91, 118.60, 117.39, 114.34, 112.21, 51.94 ppm. IR (neat) $\tilde{\nu}$ = 3319, 1680, 1581, 1494, 1442, 1314, 1246, 1082, 879, 749 cm$^{-1}$. HR-MS(ESI+): *m/z* calcd. for $C_{26}H_{22}N_2O_4$: 426.1580; found 426.1651 $[M+H]^+$. M.p.: 163 - 168°C.

## 2,2'-(naphthalene-2,6-diylbis(azanediyl))dibenzoic acid **7**

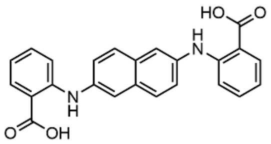

Compound **7** was synthesized according to **GP2** using **6** (1.07 g, 2.51 mmol, 1.00 eq.) and KOH (4.22 g, 75.3 mmol, 30.0 eq.). The product was obtained as an off white solid (970 mg, 2.44 mmol, 97%).

$^{1}$H NMR (400 MHz, MeOD/10% [$D_2O$ 30% *w/w* NaOD]) *δ* 7.97 – 7.94 (m, 2H), 7.63 – 7.59 (m, 2H), 7.54 – 7.52 (m, 2H), 7.35 – 7.32 (m, 2H), 7.30 – 7.26 (m, 2H), 7.25 – 7.20 (m, 2H), 6.75 – 6.71 (m, 2H) ppm. $^{13}$C NMR (101 MHz, MeOD/10% [$D_2O$ 30% *w/w* NaOD]) *δ* 167.14, 137.71, 130.39, 123.66, 122.63, 122.59, 119.24, 114.16, 113.53, 108.91, 106.33, 105.68 ppm. IR (neat) $\tilde{\nu}$ = 3324, 1654, 1577, 1496, 1448, 1294, 1163, 881, 748, 652 cm$^{-1}$. HR-MS(ESI-): *m/z* calcd. for $C_{24}H_{18}N_2O_4$: 398.1267; found 397.1189 $[M-H]^-$. M.p.: 280 - 300 °C (dec.).

## 8,16-dihydroacridino[2,1-a]acridine-5,13-dione **8**

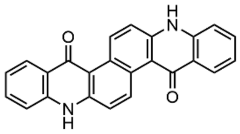

Compound **8** was synthesized according to **GP3** using **7** (200 mg, 502 µmol, 1.00 eq.) and 4 mL Eaton's reagent. The product was obtained as a dark solid (180 mg, 501 µmol, 100%).

$^{1}$H NMR (301 MHz, $D_2SO_4$) *δ* 10.40 (d, *J* = 9.9 Hz, 2H), 8.60 (d, *J* = 8.7 Hz, 2H), 8.38 (d, *J* = 9.9 Hz, 2H), 8.34 – 8.25 (m, 2H), 8.19 (d, *J* = 8.7 Hz, 2H), 8.05 – 7.93 (m, 2H) ppm. $^{13}$C NMR (101 MHz, $D_2SO_4$) *δ* 169.06, 141.55, 138.72, 138.15, 137.39, 128.21, 121.60, 120.31, 119.05, 115.94, 110.63 ppm. IR (neat) $\tilde{\nu}$ = 3269, 3102, 2981, 1613, 1533, 1476, 1163, 1046, 745, 527 cm$^{-1}$. HR-MS(EI+): *m/z* calcd. for $C_{24}H_{14}N_2O_2$: 362.1055; found 362.1021 $[M]^+$. M.p.: >300°C.

## Di-tert-butyl 5,13-dioxo-5,13-dihydroacridino[2,1-a]acridine-8,16-dicarboxylate **9**

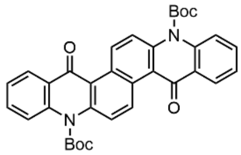


Compound **9** was synthesized according to **GP4** using **8** (250 mg, 690 µmol, 1.00 eq.), $Boc_2O$ (1.50 g, 6.90 mmol, 10.0 eq.), DMAP (84.3 mg, 690 µmol, 1.00 eq.) and 2,6-Lutidine (368 mg, 400 µL, 3.45 µmol, 5.00 eq.) and DCM as the solvent. The product was precipitated two times from methanol to obtain a yellow solid (160 mg, 284 µmol, 41%).

$^{1}$H NMR (600 MHz, $CDCl_3$) *δ* 10.56 (d, *J* = 9.5 Hz, 2H), 8.57 – 8.51 (m, 2H), 7.80 (d, *J* = 9.6 Hz, 2H), 7.73 – 7.65 (m, 4H), 7.46 – 7.40 (m, 2H), 1.74 (s, 18H) ppm. $^{13}$C NMR (151 MHz, $CDCl_3$) *δ* 180.28, 151.83, 139.69, 137.71, 133.28, 133.18, 128.41, 127.48, 125.04, 123.78, 119.87, 116.57, 115.40, 87.21, 27.84 ppm. IR (neat) $\tilde{\nu}$ = 2986, 1753, 1626, 1598, 1517, 1466, 1244, 1146, 1117, 744 $cm^{-1}$. HR-MS(ESI+): *m/z* calcd. for $C_{34}H_{30}N_2O_6$: 562.2104; found 563.2189 $[M+H]^+$. M.p.: 230 - 240 °C (dec.).

## 5,13-bis((triisopropylsilyl)ethynyl)acridino[2,1-a]acridine **3**

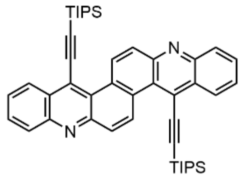


In an oven-dried Schlenk tube under argon atmosphere, 2.5 M *n*-BuLi in *n*-hexane (327 mg, 2.05 mL, 5.12 mmol, 8.00 eq.) was slowly added to a solution of TIPS-acetylene (1.05 g, 1.29 mL, 5.76 mmol, 9.00 eq.) in dry THF at -78 °C. After stirring for 1 h, **9** (360 mg, 640 µmol, 1.00 eq.) was added to the solution and the reaction was allowed to stir overnight while thawing. The reaction was subsequently quenched with methyl iodide (1.82 g, 796 µl, 12.8 mmol, 20.0 eq.) and stirred for 8 h. 2 mL of saturated aqueous $NH_4Cl$ solution was added and the layers were separated. After concentrating in vacuo, the crude product was redissolved in DCM and washed with water, dried over $Na_2SO_4$ and evaporated under reduced pressure. The solid was then dissolved in little DCM and TFA (2.48 g, 1.67 mL, 25.6 mmol, 40.0 eq.) was added to the stirring mixture at 0 °C. After stirring for 30 min an excess of saturated aqueous $NaHCO_3$ solution was added and stirred for 5 min. The layers were separated and the organic phase was dried over $Na_2SO_4$ and evaporated under reduced pressure. The crude product was purified using silica gel column chromatography. The column was flushed with petrol ether before increasing the polarity to PE:EE 95:5. The product is obtained as a yellow solid (108 mg, 156 µmol, 24%). For use in OFET, the solid was recrystallized three times by layering HPLC-grade methanol, ethyl acetate and *n*-hexane over a solution of **3** in HPLC-grade DCM.

$^{1}$H NMR (600 MHz, $CDCl_3$) *δ* 10.73 (d, *J* = 9.5 Hz, 2H), 8.84 – 8.81 (m, 2H), 8.35 – 8.24 (m, 4H), 7.90 – 7.86 (m, 2H), 7.74 – 7.70 (m, 2H), 1.43 – 1.35 (m, 6H), 1.30 (d, *J* = 7.2 Hz, 36H) ppm. $^{13}$C NMR (151 MHz, $CDCl_3$) *δ* 148.81, 147.79, 130.78, 130.15, 130.09, 129.60, 128.62, 128.23, 127.39, 127.20, 126.63, 123.76, 113.09, 104.26, 19.01, 11.65 ppm. IR (neat) $\tilde{\nu}$ = 2934, 2861, 2132, 1525, 1457, 1008, 879, 757, 668, 505 $cm^{-1}$. HR-MS(MALDI+): *m/z* calcd. for $C_{46}H_{54}N_2Si_2$: 690.3826; found 691.3897 $[M+H]^+$. M.p.: 270 - 280 °C (dec.). Elemental analysis [%] = calcd. for $C_{46}H_{54}N_2Si_2$: C 79.94, H 7.88, N 4.05, Si 8.13; found: C 79.98, H 7.73, N 3.75.

## Dimethyl 2,2'-(naphthalene-1,5-diylbis(azanediyl))dibenzoate **11**

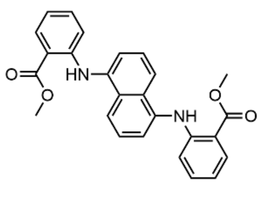


Compound **11** was synthesized according to **GP1** from 1,5-dibromonaphthalene **10** (1.25 g, 4.37 mmol, 1.00 eq.), methyl-anthranilate (1.98 g, 1.94 mL, 13.1 mmol, 3.00 eq.), cesium carbonate (5.70 g, 17.48 mmol, 4.00 eq.) and RuPhos-Pd G1 (143 mg, 175 µmol, 4 mol%). The reaction mixture was stirred at 120 °C overnight, washed with water, extracted with DCM, dried and evaporated under reduced pressure. The crude product was then precipitated twice from methanol by dissolving it in DCM, diluting with methanol and removing the DCM under reduced pressure to furnish the product as yellow crystals (1.69 g, 3.96 mmol, 91%).

$^{1}$H NMR (400 MHz, $CDCl_3$) *δ* 9.83 (s, 2H), 8.05 – 8.00 (m, 2H), 7.97 (d, *J* = 8.4 Hz, 2H), 7.55 (d, *J* = 7.3 Hz, 2H), 7.50 – 7.43 (m, 2H), 7.30 – 7.23 (m, 2H), 6.99 – 6.95 (m, 2H), 6.77 – 6.70 (m, 2H), 3.97 (s, 6H) ppm. $^{13}$C NMR (101 MHz, $CDCl_3$) *δ* 169.42, 149.54, 137.30, 134.38, 131.67, 131.34, 126.24, 121.44, 119.95, 117.00, 114.51, 111.73, 51.99 ppm. IR (neat) $\tilde{\nu}$ = 2954, 1677, 1578, 1521, 1255, 1226, 1089, 736, 515 $cm^{-1}$. HR-MS(ESI+): *m/z* calcd. for $C_{26}H_{22}N_2O_4$: 426.1580; found 449.1472 $[M+Na]^+$. M.p.: 235 - 240°C.

## 2,2'-(naphthalene-1,5-diylbis(azanediyl))dibenzoic acid **12**

Compound **12** was synthesized according to **GP2** using **11** (1.50 g, 3.52 mmol, 1.00 eq.) and KOH (5.92 g, 105 mmol, 30.0 eq.). The product was obtained as a beige solid (1.38 g, 3.52 mmol, 99%).

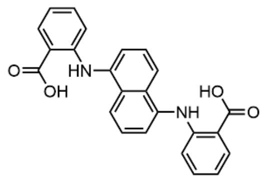


$^1$H NMR (600 MHz, DMSO-*d6*) $\delta$ 13.23 (s, 2H), 10.13 (s, 2H), 7.98 – 7.95 (m, 2H), 7.84 – 7.81 (m, 2H), 7.61 – 7.54 (m, 4H), 7.37 – 7.32 (m, 2H), 6.98 – 6.95 (m, 2H), 6.80 – 6.77 (m, 2H) ppm. $^{13}$C NMR (151 MHz, DMSO) $\delta$ 170.43, 148.37, 136.91, 134.28, 131.83, 130.04, 126.52, 119.98, 118.36, 117.29, 113.97, 112.38 ppm. IR (neat) $\tilde{\nu}$ = 3307, 2735, 2638, 1658, 1577, 1415, 1247, 1157, 737, 517 cm$^{-1}$. HR-MS(ESI-): *m/z* calcd. for $C_{24}H_{18}N_2O_4$: 398.1267; found 397.1196 [M-H]$^-$. M.p.: >300 °C.

## 5,13-dihydroacridino[4,3-c]acridine-8,16-dione **13**

Compound **13** was synthesized according to **GP3** using **12** (1.00 g, 2.51 mmol, 1.00 eq.) and 20 mL Eaton's reagent. The product was obtained as a brown solid (880 mg, 2.43 mmol, 97%).

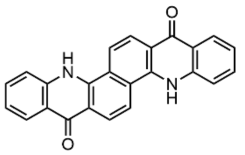


$^1$H NMR (400 MHz, $D_2SO_4$) $\delta$ 9.08 (d, *J* = 9.5 Hz, 2H), 8.99 (d, *J* = 9.4 Hz, 2H), 8.70 – 8.66 (m, 2H), 8.47 (d, *J* = 8.8 Hz, 2H), 8.41 – 8.34 (m, 2H), 8.05 – 7.99 (m, 2H) ppm. $^{13}$C NMR (101 MHz, $D_2SO_4$) $\delta$ 168.27, 140.64, 139.12, 138.13, 128.67, 125.75, 123.08, 122.79, 119.65, 119.08, 115.87, 114.82 ppm. IR (neat) $\tilde{\nu}$ = 3277, 3190, 3068, 3007, 1574, 1531, 1430, 1185, 745, 487 cm$^{-1}$. HR-MS(APCI): *m/z* calcd. for $C_{24}H_{14}N_2O_2$: 362.1055; found 36.1123 [M+H]$^+$. M.p.: >300°C.

## Acridino[4,3-c]acridine-8,16-diyl di-tert-butyl bis(carbonate) **14a**

Compound **14a** was synthesized according to **GP4** using powdered **13** (500 mg, 1.38 mmol, 1.00 eq.), $Boc_2O$ (3.01 g, 13.8 mmol, 10.0 eq.), DMAP (33.7 mg, 275 µmol, 0.20 eq.) and 2,6-Lutidine (591 mg, 639 µL, 5.52 mmol, 4.00 eq.) and DCM as the solvent. The product was precipitated two times from methanol and filtered over a silica pad (DCM) to obtain a yellow solid (380 mg, 675 µmol, 49%).

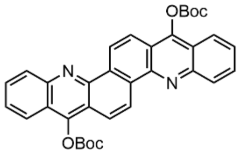


$^1$H NMR (700 MHz, $CDCl_3$) $\delta$ 9.66 (d, *J* = 9.2 Hz, 2H), 8.47 – 8.43 (m, 2H), 8.29 (d, *J* = 9.1 Hz, 2H), 8.20 – 8.17 (m, 2H), 7.90 – 7.86 (m, 2H), 7.71 – 7.66 (m, 2H), 1.64 (s, 18H) ppm. $^{13}$C NMR (176 MHz, $CDCl_3$) $\delta$ 151.19, 150.96, 149.34, 147.85, 132.09, 130.42, 130.37, 127.05, 124.21, 121.60, 120.47, 120.25, 119.28, 85.29, 27.80 ppm. IR (neat) $\tilde{\nu}$ = 3067, 2967, 1759, 1599, 1369, 1243, 1139, 763, 719, 656 cm$^{-1}$. HR-MS(MALDI+): *m/z* calcd. for $C_{34}H_{30}N_2O_6$: 562.2104; found 585.1997 [M+Na]$^+$. M.p.: > 300 °C.

## Acridino[4,3-c]acridine-8,16-diyl bis(trifluoromethanesulfonate) **14b**

In a heat-gun dried Schlenk tube under argon with fine glass shards, powdered compound **13** (200 mg, 551 µmol, 1.00 eq.) was suspended in 30 mL dry DCM. The suspension was cooled down to 0 °C and DMAP (27.0 mg, 221 µmol, 0.40 eq.), 2,6-lutidine (1.18 g, 1.28 mL, 11.0 mmol, 20.0 eq.) and freshly distilled (over $P_2O_5$) $Tf_2O$ (1.56 g, 926 µL, 5.52 mmol, 10.0 eq.) were added. The reaction mixture was stirred for 2 h at 0 °C before letting it thaw to room temperature and stirring overnight. The resulting yellow suspension was pipetted onto a Buchner filter paying attention not to take up glass shards. The filter cake was washed with cold DCM and methanol to afford the pure product as a bright yellow solid (310 mg, 495 µmol, 89%).

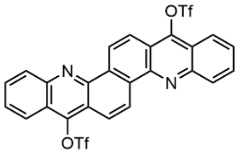


$^1$H NMR (600 MHz, $CDCl_3$) 9.81 – 9.76 (m, 2H), 8.57 – 8.52 (m, 2H), 8.52 – 8.47 (m, 2H), 8.39 – 8.35 (m, 2H), 8.01 – 7.95 (m, 2H), 7.86 – 7.80 (m, 2H) ppm. Due to low solubility, no $^{13}$C NMR could be recorded. $^{19}$F {$^1$H} NMR (283 MHz, $CDCl_3$) $\delta$ -72.10 ppm. IR (neat) $\tilde{\nu}$ = 1625, 1409, 1212, 1130, 1004, 896, 757, 721, 639, 500 cm$^{-1}$. HR-MS(MALDI+): *m/z* calcd. for $C_{26}H_{12}F_6N_2O_6S_2$: 626.0041; found 627.0110 [M+H]$^+$. M.p.: 200 - 210 °C (dec.).

## 8,16-bis((triisopropylsilyl)ethynyl)acridino[4,3-c]acridine **4**

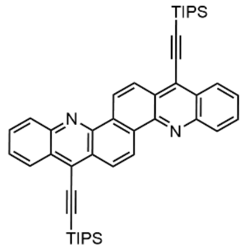


In a heat-gun dried Schlenk tube under argon with fine glass shards compound **14b** (150 mg, 239 mmol, 1.00 eq.) was suspended in 20 mL dry, degassed THF. Then freshly prepared TIPS-ethynyltrimethylstannane (1.65 g, 4.79 mmol, 20.0 eq.), $Pd_2(dba)_3$ (21.9 mg, 23.9 µmol 10 mol%) and tri-*tert*-butylphosphinetetrafluoroborate (13.8 mg, 47.8 µmol, 20 mol%) were added. The reaction mixture was stirred at 80 °C for 7 d. The resulting suspension was filtered and eluted with DCM to separate from the glass shards. The filtrate was evaporated, adsorbed on celite and purified using silica gel column chromatography. The column was flushed with petrol ether before increasing the polarity to PE:DCM 9:1. The product is obtained as a bright yellow solid (87.0 mg, 123 µmol, 51%). For use in OFET, the solid was recrystallized three times by layering HPLC-grade methanol, ethyl acetate and hexane over a solution of 4 in HPLC-grade DCM.

$^1$H NMR (400 MHz, $CDCl_3$) *δ* 9.66 (d, *J* = 9.2 Hz, 2H), 8.73 (d, *J* = 9.2 Hz, 2H), 8.58 – 8.53 (m, 2H), 8.44 – 8.39 (m, 2H), 7.89 – 7.82 (m, 2H), 7.70 – 7.64 (m, 2H) 1.42 – 1.35 (m, 6H), 1.33 (d, *J* = 5.2 Hz, 36H). $^{13}$C NMR (101 MHz, $CDCl_3$) *δ* 147.85, 146.36, 132.27, 130.63, 130.08, 127.53, 127.26, 127.18, 127.03, 126.61, 125.40, 124.51, 108.65, 101.35, 19.04, 11.64 ppm. IR (neat) $\tilde{\nu}$ = 2938, 2864, 1581, 1477, 1258, 1013, 930, 880, 750, 676 cm$^{-1}$. HR-MS(MALDI+): *m/z* calcd. for $C_{46}H_{54}N_2Si_2$: 690.3826; found 691.3891 [M+H]$^+$. M.p.: >300 °C. Elemental analysis [%] = calcd. for $C_{46}H_{54}N_2Si_2$: C 79.94, H 7.88, N 4.05, Si 8.13; found: C 80.13, H 8.06, N 3.74.

## Dimethyl 2,2'-((1,5-naphthyridine-4,8-diyl)bis(azanediyl))dibenzoate **S1**

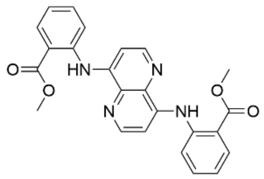


Compound **S1** was synthesized according to **GP1** from 1,5-dibromonaphthyridine[10] (120 mg, 416 µmol, 1.00 eq.), commercially available methylanthranilate (189 mg, 185 µL, 1.25 mmol, 3.00 eq.), cesium carbonate (543 mg, 1.67 mmol, 4.00 eq.), RuPhos-Pd G1 (34.0 mg, 41.6 µmol, 10 mol%), $Pd_2(dba)_3$ (6.30 mg, 41.7 µmol, 10 mol%) and XPhos (19.9 mg, 41.7 µmol, 10 mol%). The reaction mixture was stirred at 120 °C for 7 d, washed with water, extracted with DCM, dried and evaporated under reduced pressure. The crude product was purified using silica gel column chromatography with DCM as the eluent. The product is obtained as a light yellow crystalline solid (37 mg, 86.3 µmol, 21%).
$^1$H NMR (400 MHz, $CDCl_3$) δ 11.22 (s, 2H), 8.65 (d, *J* = 5.3 Hz, 2H), 8.10 (m, 2H), 7.83 (m, 2H), 7.56 (m, 2H), 7.50 (d, *J* = 5.3 Hz, 2H), 7.17 – 6.98 (m, 2H), 3.97 (s, 6H). $^{13}$C NMR (101 MHz, $CDCl_3$) δ 167.87, 149.05, 145.83, 142.77, 136.05, 133.83, 132.21, 121.64, 119.05, 118.32, 104.01, 52.48. IR (neat) $\tilde{\nu}$ = 3278, 2949, 1694, 1535, 1255, 1082, 832, 756, 730, 627 cm$^{-1}$. HR-MS(ESI+): *m/z* calcd for $C_{24}H_{20}N_4O_4$: 428.1485; found 429.1557 (M+H$^+$). M.p.: 245 - 250 °C.

## 2,2'-((1,5-naphthyridine-4,8-diyl)bis(azanediyl))dibenzoic acid **17**

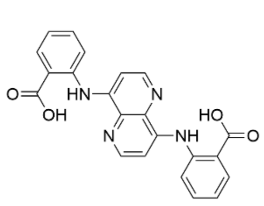


Compound **X** was synthesized according to **GP2** using **X** (320 mg, 746 µmol, 1.00 eq.) and KOH (1.26 g, 22.4 mmol, 30.0 eq.). The product was obtained as a yellow solid (287mg, 716 µmol, 96%).
$^1$H NMR (301 MHz, DMSO) δ 13.38 (s, 2H), 11.21 (s, 2H), 8.59 (d, *J* = 5.2 Hz, 2H), 8.04 (m, 2H), 7.95 – 7.75 (m, 2H), 7.65 (m, 2H), 7.58 (d, *J* = 5.3 Hz, 2H), 7.20 – 7.09 (m, 2H). $^{13}$C NMR (151 MHz, DMSO) δ 168.72, 149.00, 144.95, 141.77, 135.21, 133.74, 131.96, 121.65, 118.90, 118.74, 104.01. IR (neat) $\tilde{\nu}$ = 3496, 3279, 1699, 1577, 1535, 1452, 1267, 1242, 1216, 744 cm$^{-1}$. HR-MS(ESI-): *m/z* calcd for $C_{22}H_{16}N_4O_4$: 400.1172; found 399.1100 (M+H$^+$). M.p.: >300 °C.

9-((triisopropylsilyl)ethynyl)acridine **2**

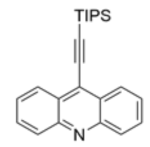


Compound **X** was synthesized from *n*-Boc-Acridone In an oven-dried Schlenk tube under argon atmosphere, 2.5 M *n*-BuLi in *n*-hexane (173 mg, 1.08 mL, 2.71 mmol, 4.00 eq.) was slowly added to a solution of TIPS-acetylene (617 mg, 759 µL, 3.39 mmol, 5.00 eq.) in dry THF at -78 °C. After stirring for 1 h, *N*-Boc-acridone (200 mg, 677 µmol, 1.00 eq.) was added to the solution and the reaction was allowed to stir overnight while thawing. The reaction was subsequently quenched with methyl iodide (961 mg, 417 µl, 6.77 mmol, 10.0 eq.) and stirred for 8 h. A small volume of saturated aqueous ammonium chloride solution was added and the layers were separated. After concentrating in vacuo, the crude product was redissolved in DCM and washed with water, dried over sodium sulfate and evaporated under reduced pressure. The solid was then dissolved in little DCM and TFA (2.63 g, 1.76 mL, 27.09 mmol, 40.0 eq.) was added to the stirring mixture at 0 °C. After stirring for 30 min an excess of saturated aqueous $NaHCO_3$ solution was added and stirred for 5 min. The layers were separated and the organic phase was dried over $Na_2SO_4$ and evaporated under reduced pressure. The crude product was purified using silica gel column chromatography. The column is flushed with petrol ether before increasing the polarity to pure DCM. The product is obtained as a reddish yellow oil that solidified under high vacuum overnight (140 mg, 395 µmol, 58%).

$^1$H NMR (700 MHz, Chloroform-*d*) δ 8.52 (m, 2H), 8.25 (m, 2H), 7.80 (m, 2H), 7.63 (m, 2H), 1.36 – 1.29 (m, 3H), 1.27 (d, *J* = 7.0 Hz, 18H). $^{13}$C NMR (176 MHz, $CDCl_3$) δ 148.59, 130.50, 129.98, 128.15, 127.08, 126.85, 126.81, 109.12, 100.99, 18.97, 11.53. IR (neat) $\tilde{\nu}$ = 2941, 2866, 1555, 1463, 1408, 1060, 879, 861, 755, 653 cm$^{-1}$. HR-MS(MALDI+): *m/z* calcd for $C_{24}H_{29}NSi$: 359.2069; found 360.2150 (M+H$^+$). M.p.: 71 – 74 °C.

## 11. **NMR spectra**

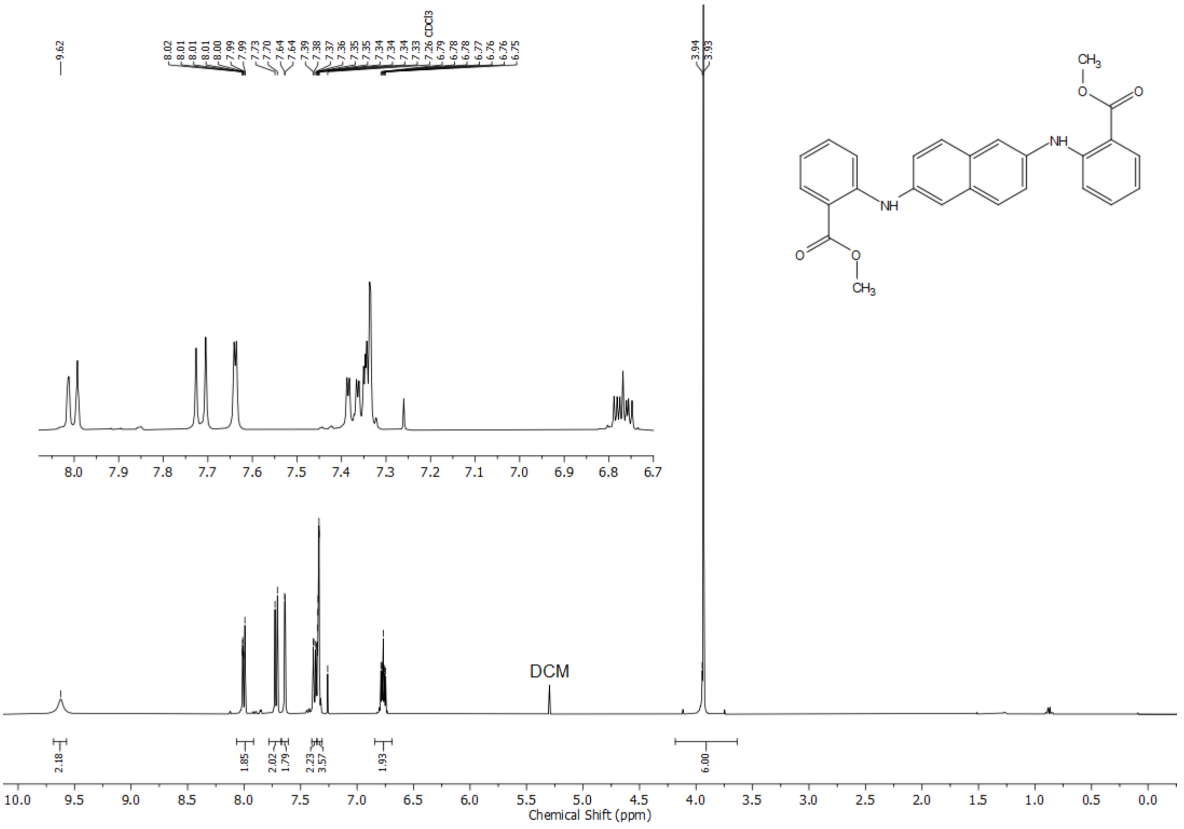


Figure S20. $^{1}$H NMR spectrum (400 MHz, $CDCl_3$) of **6**.

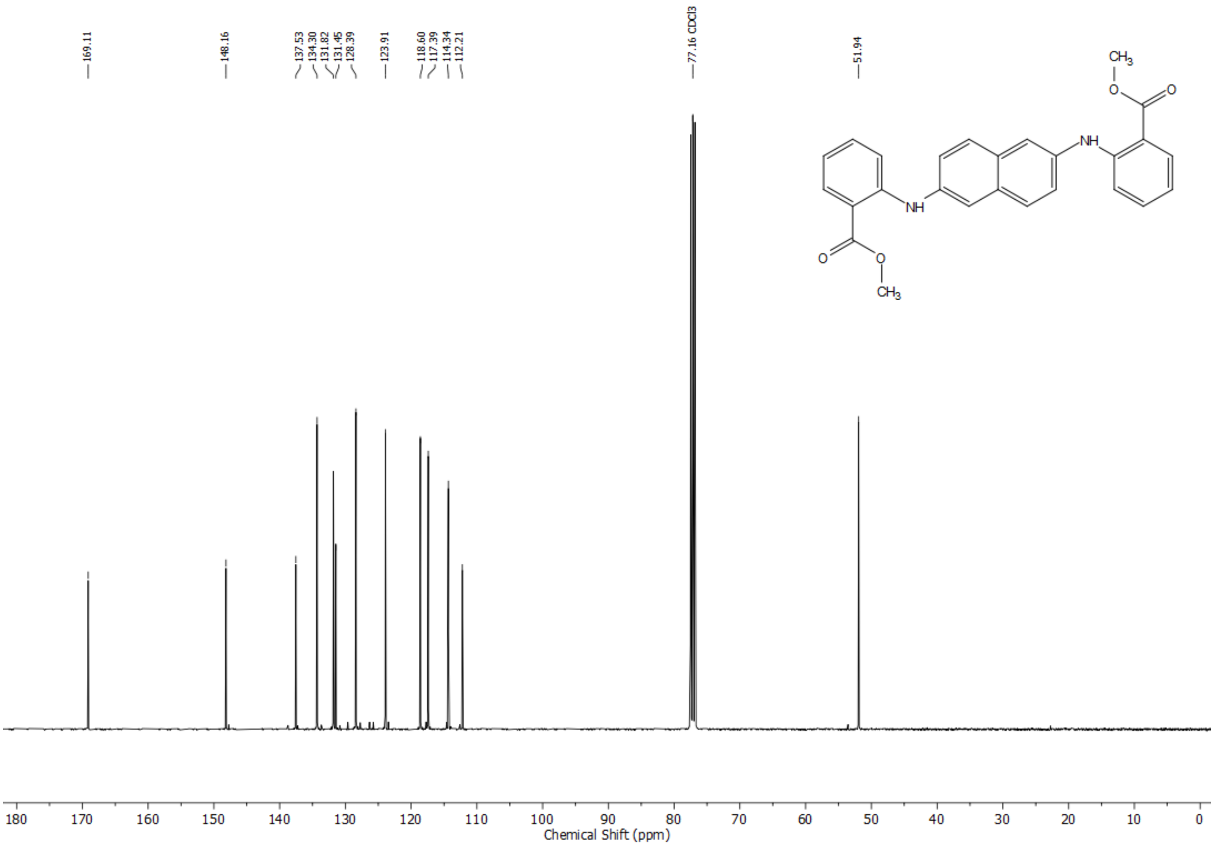


Figure S21. $^{13}$C NMR spectrum (101 MHz, $CDCl_3$) of **6**.

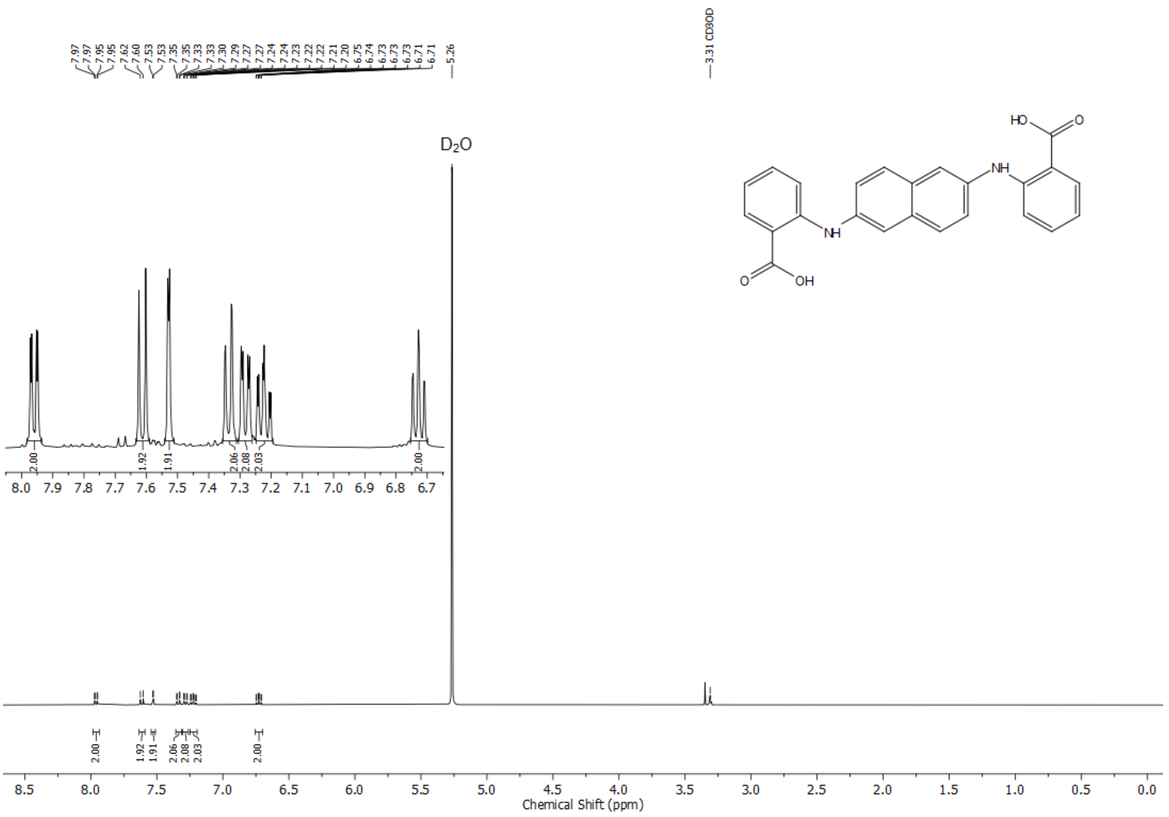

Figure S22. $^{1}H$ NMR spectrum (400 MHz MeOD/10% [$D_2O$ 30% w/w NaOD]) of **7**.

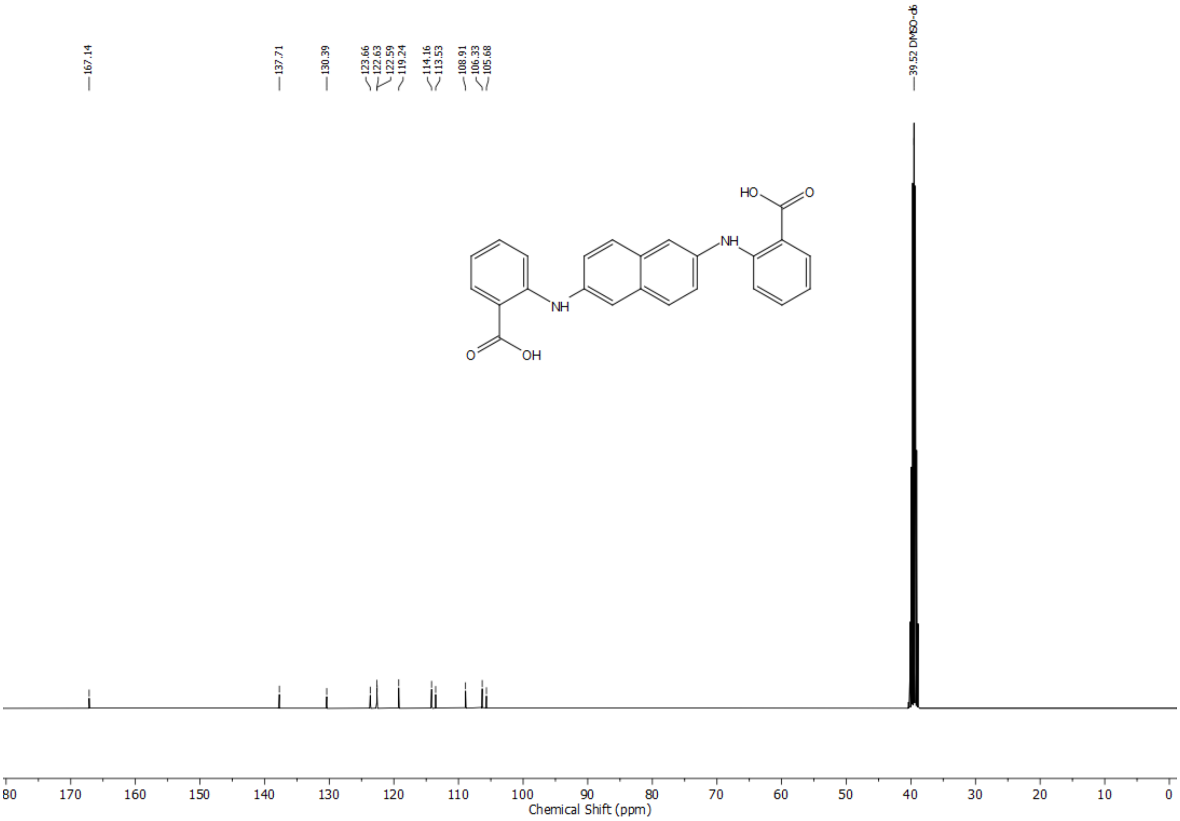

Figure S23. $^{13}C\{^{1}H\}$ NMR spectrum (101 MHz, MeOD/10% [$D_2O$ 30% w/w NaOD]) of **7**.

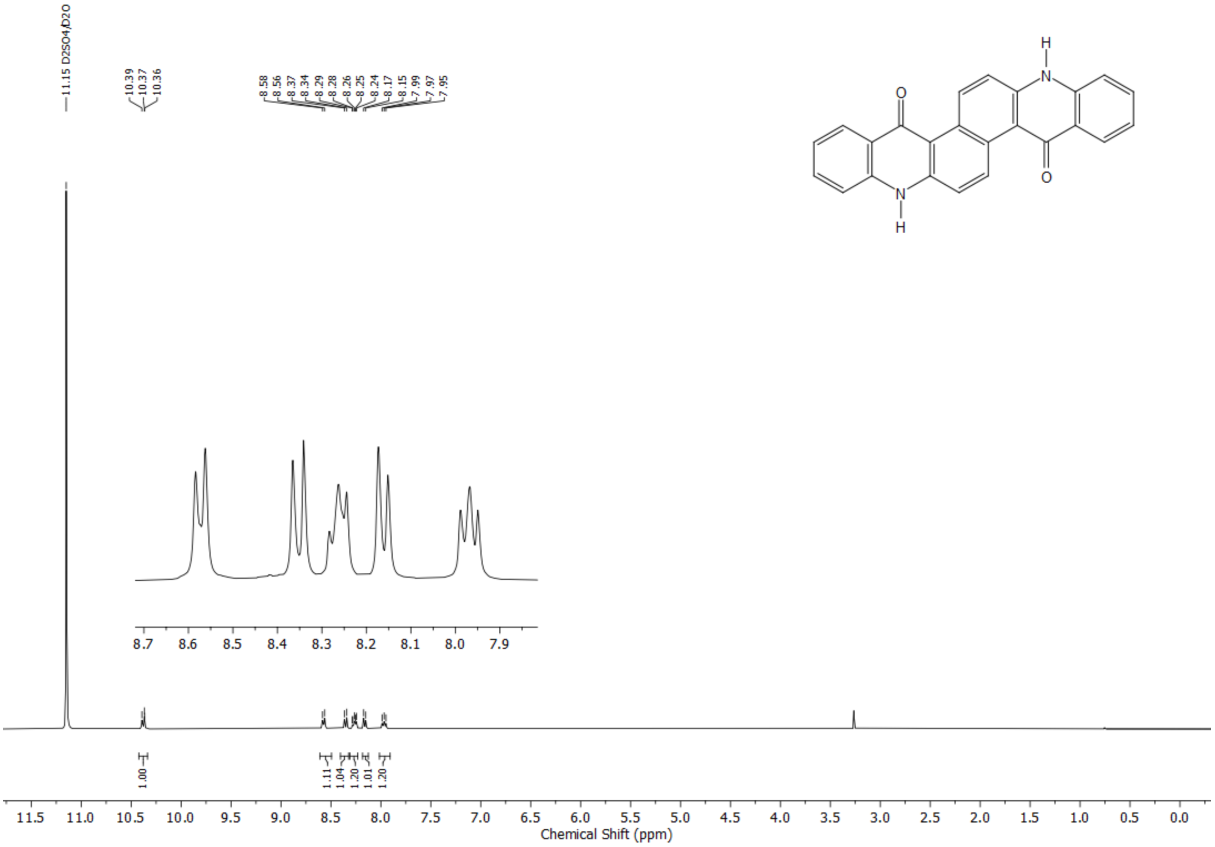


Figure S24. $^{1}$H NMR spectrum (400 MHz, 95% $D_2SO_4$ / 5% $D_2O$) of **8**. One proton is masked by the solvent residual signal.

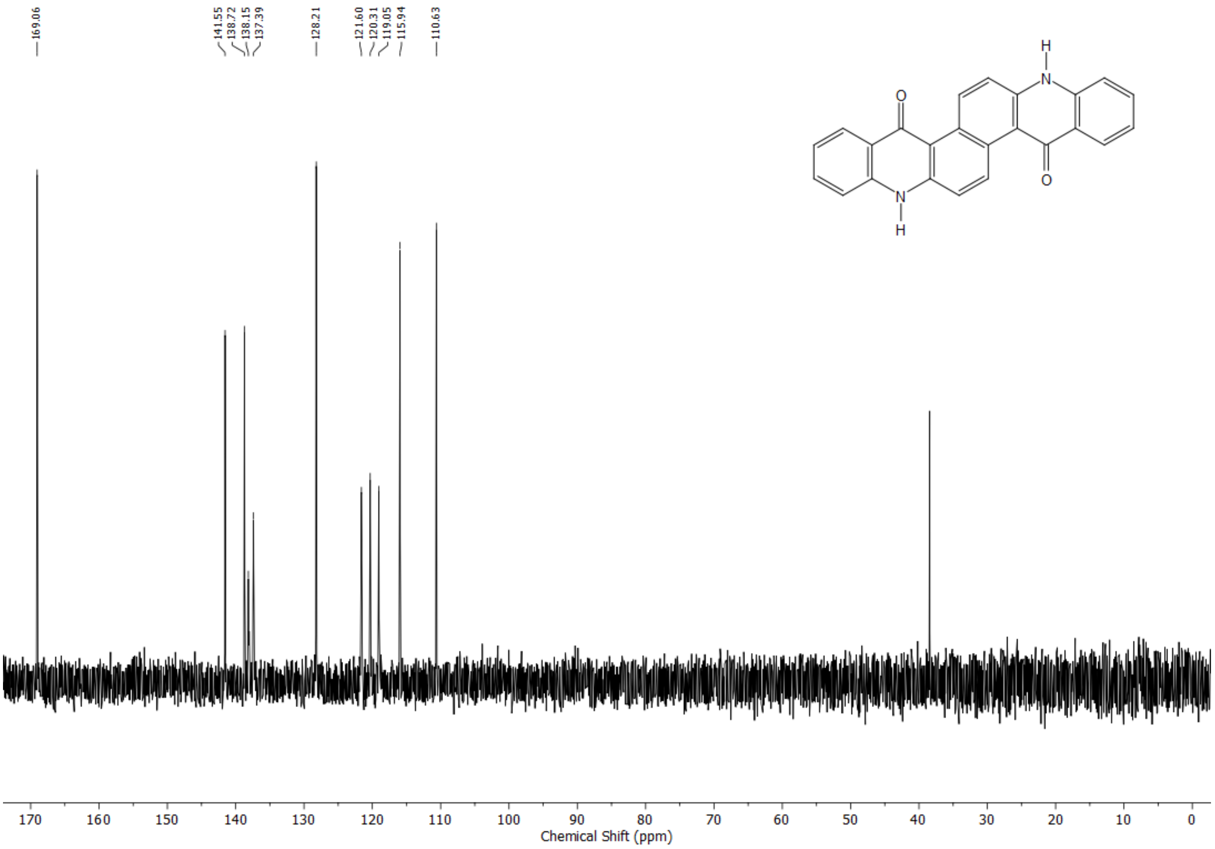


Figure S25. $^{13}C\{^1H\}$ NMR spectrum (101 MHz 95% $D_2SO_4$ / 5% $D_2O$) of **8**.

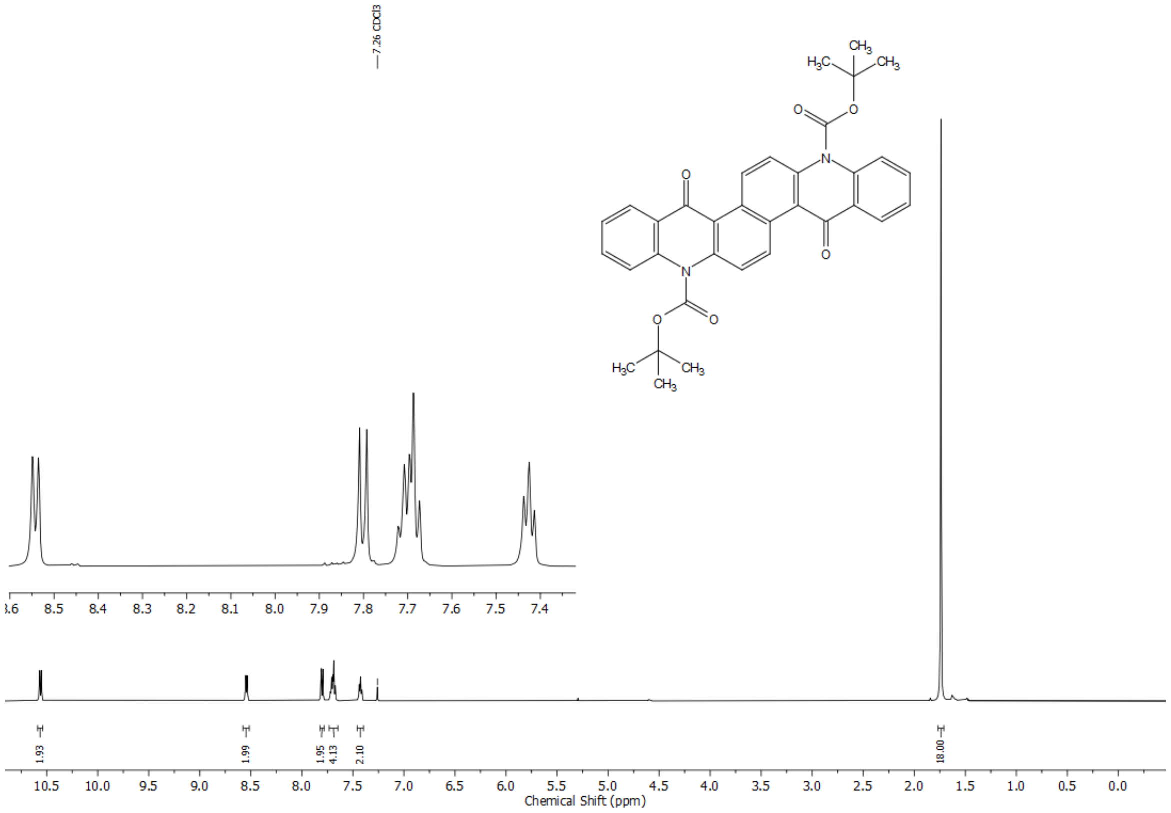


Figure S26. $^{1}H$ NMR spectrum (600 MHz, $D_2SO_4$) of **9**.

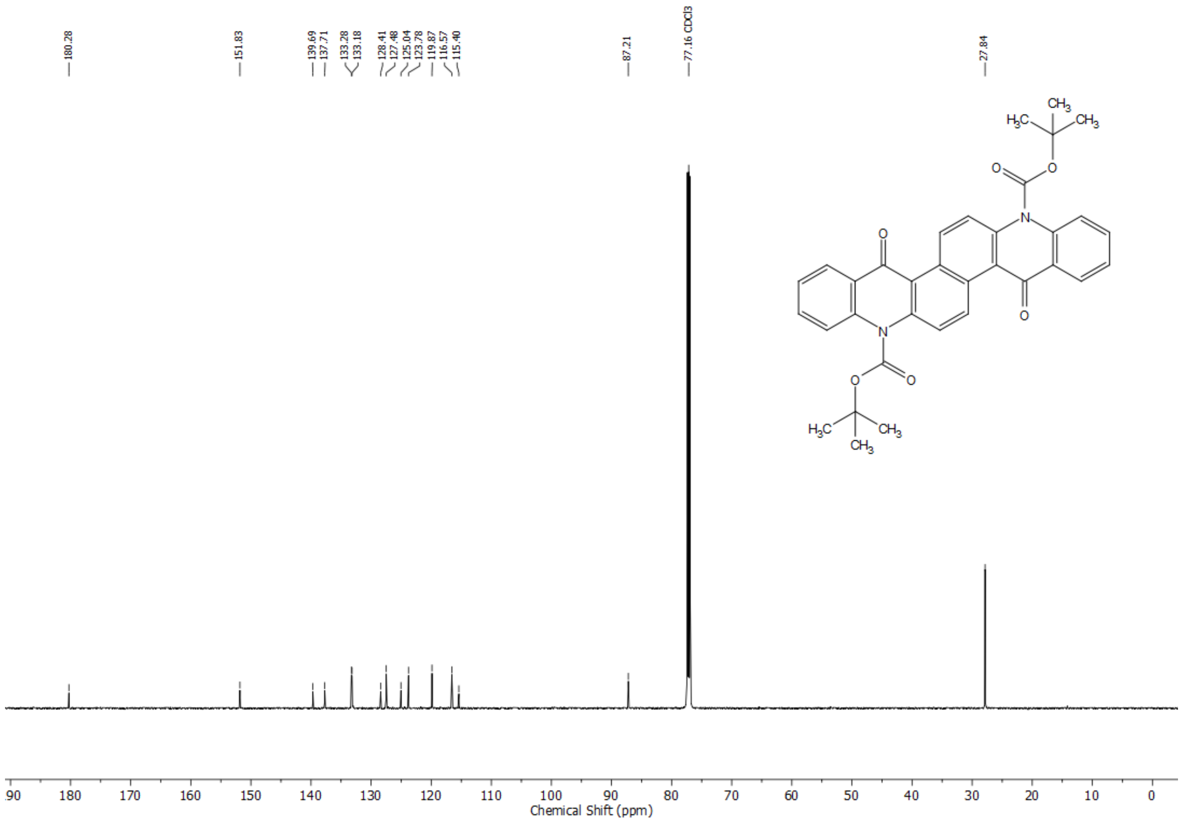


Figure S27. $^{13}C\{^{1}H\}$ NMR spectrum (151 MHz, $D_2SO_4$) of **9**.

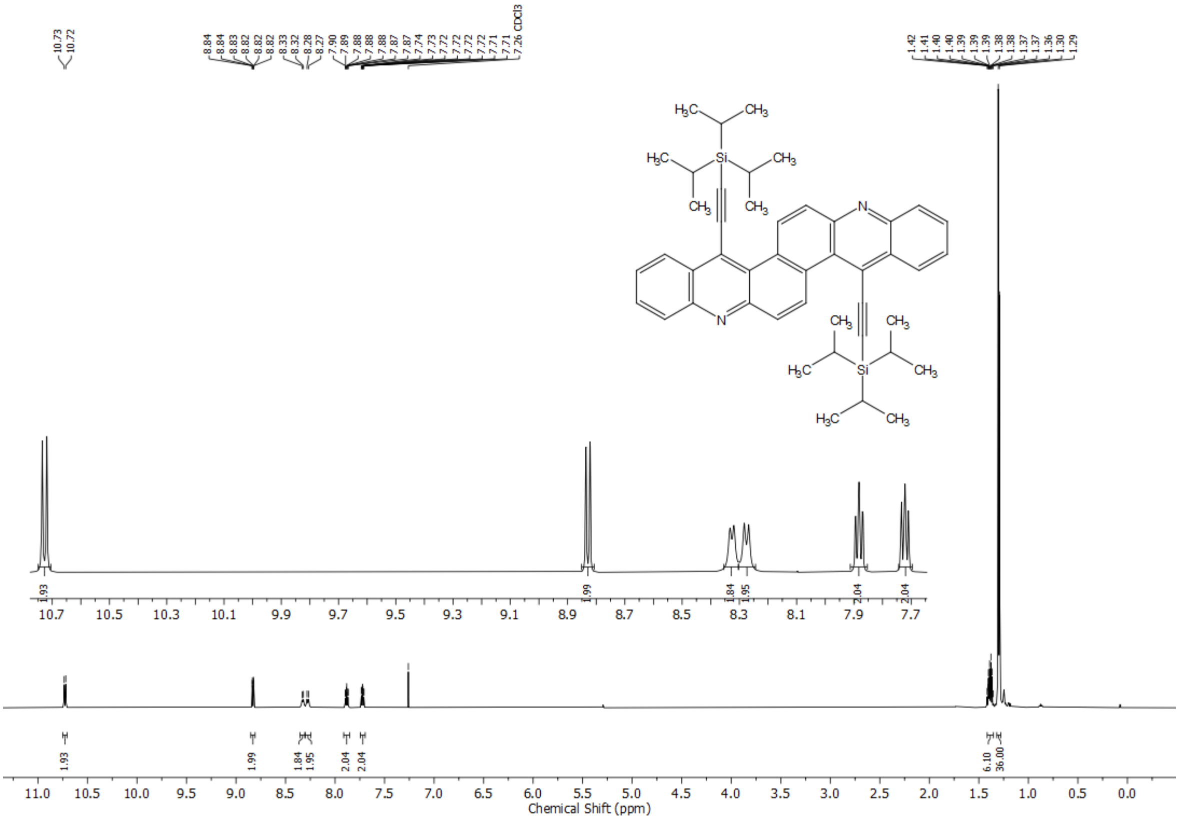

Figure S28. $^{1}H$ NMR spectrum (600 MHz, $CDCl_3$) of **3**.

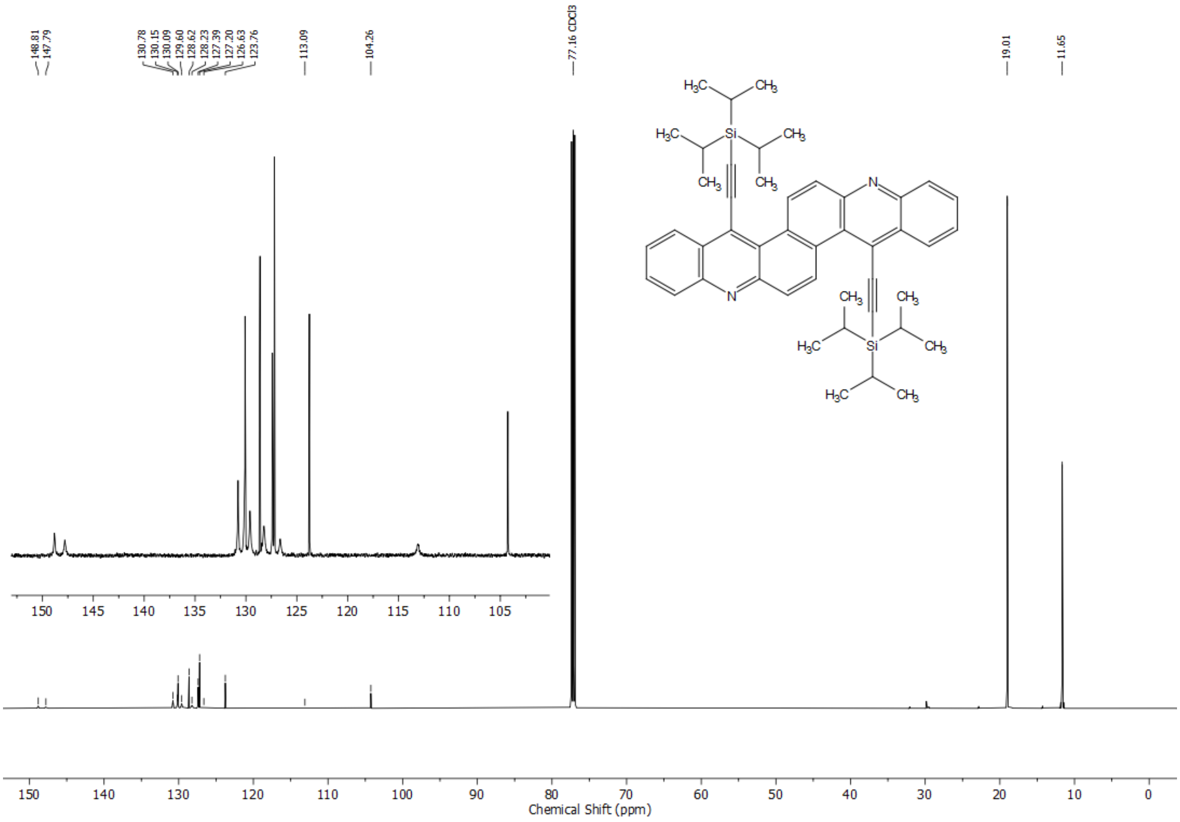

Figure S29. $^{13}C\{^{1}H\}$ NMR spectrum (151 MHz, $CDCl_3$) of **3**.

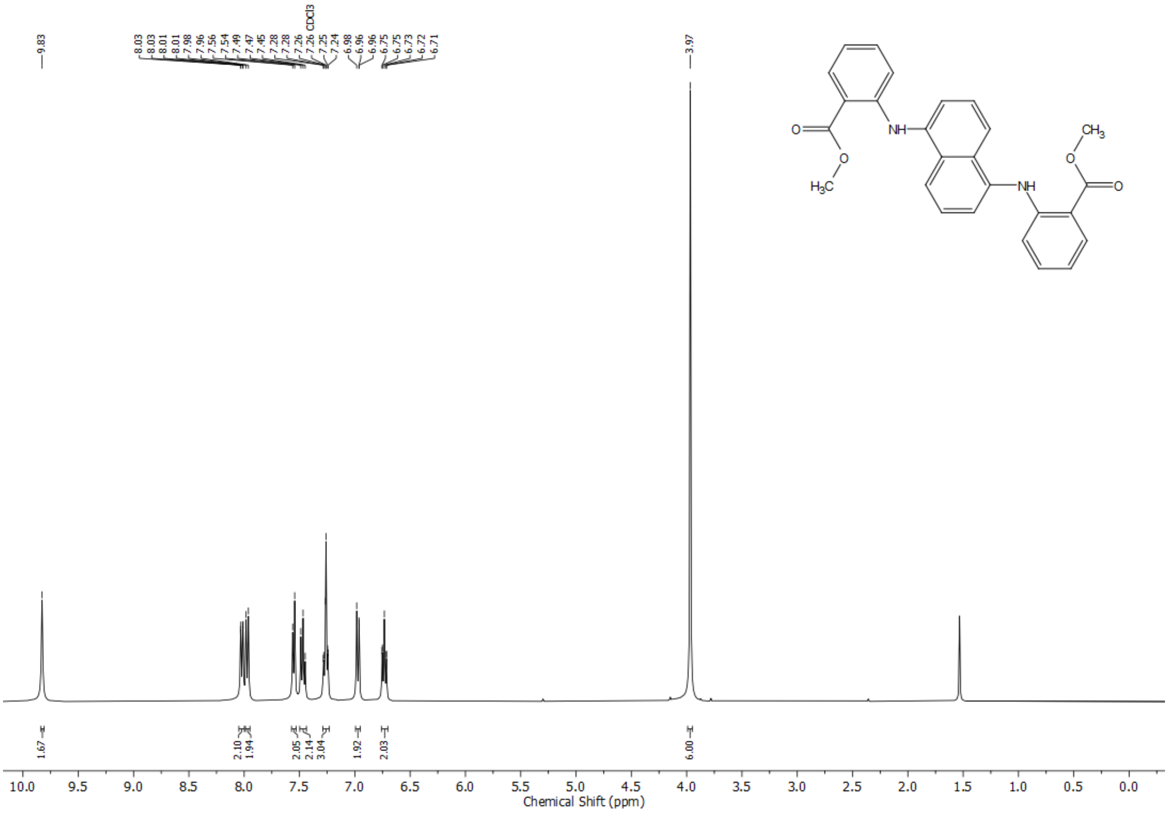

Figure S30. $^{1}H$ NMR spectrum (400 MHz, $CDCl_3$) of **11**.

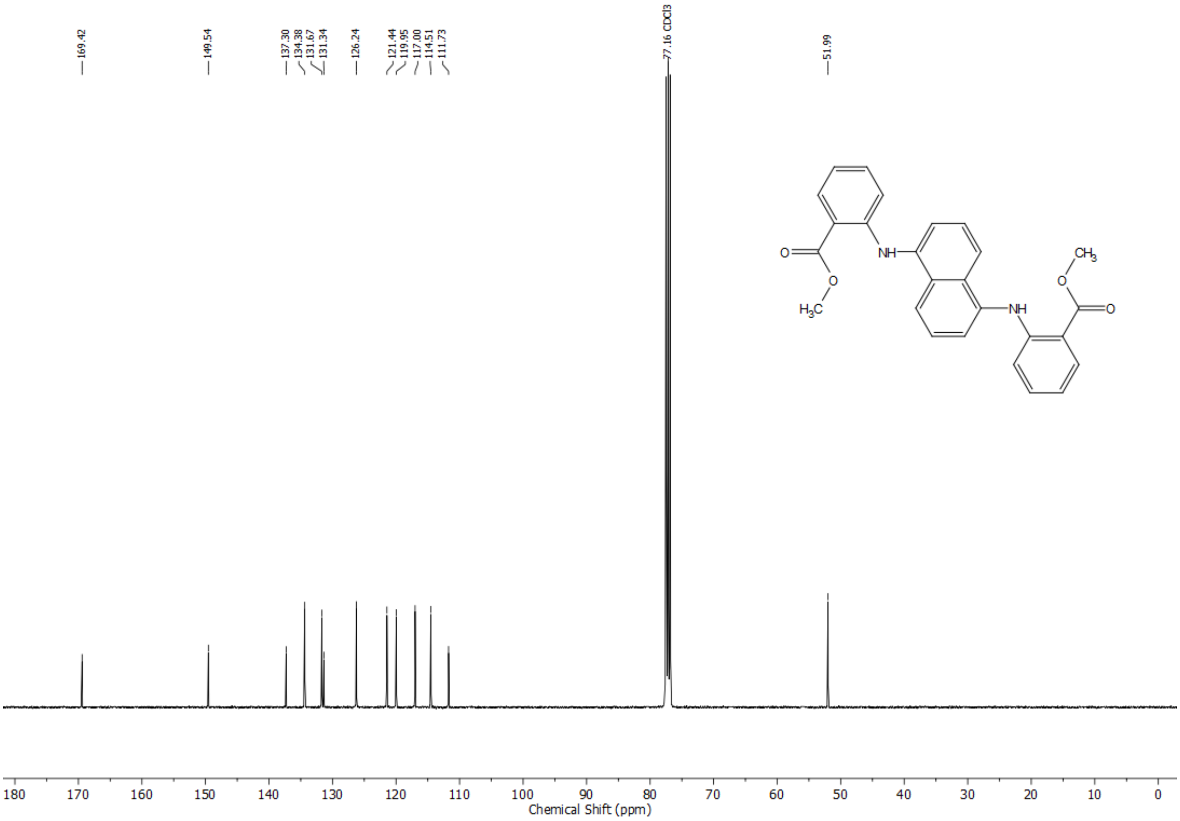

Figure S31. $^{13}C\{^{1}H\}$ NMR spectrum (101 MHz, $CDCl_3$) of **11**.

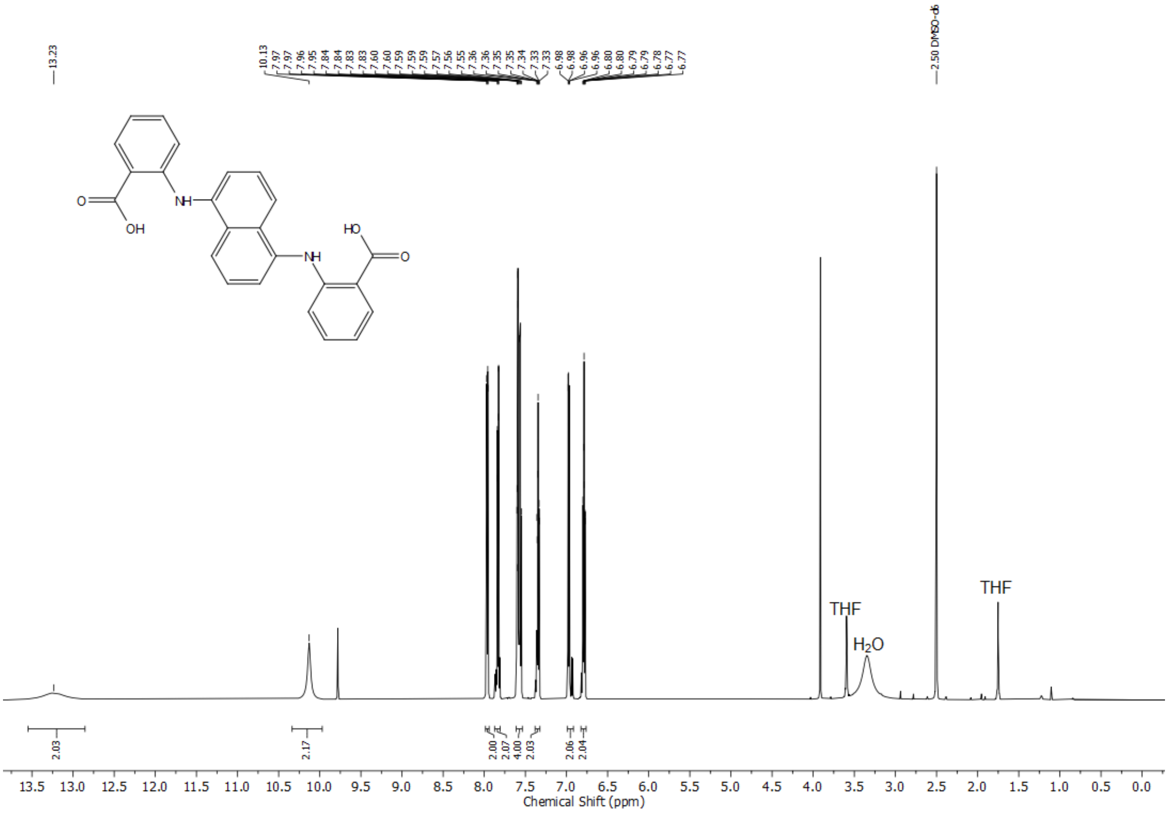


Figure S32. $^{1}$H NMR spectrum (600 MHz, DMSO) of **12**.

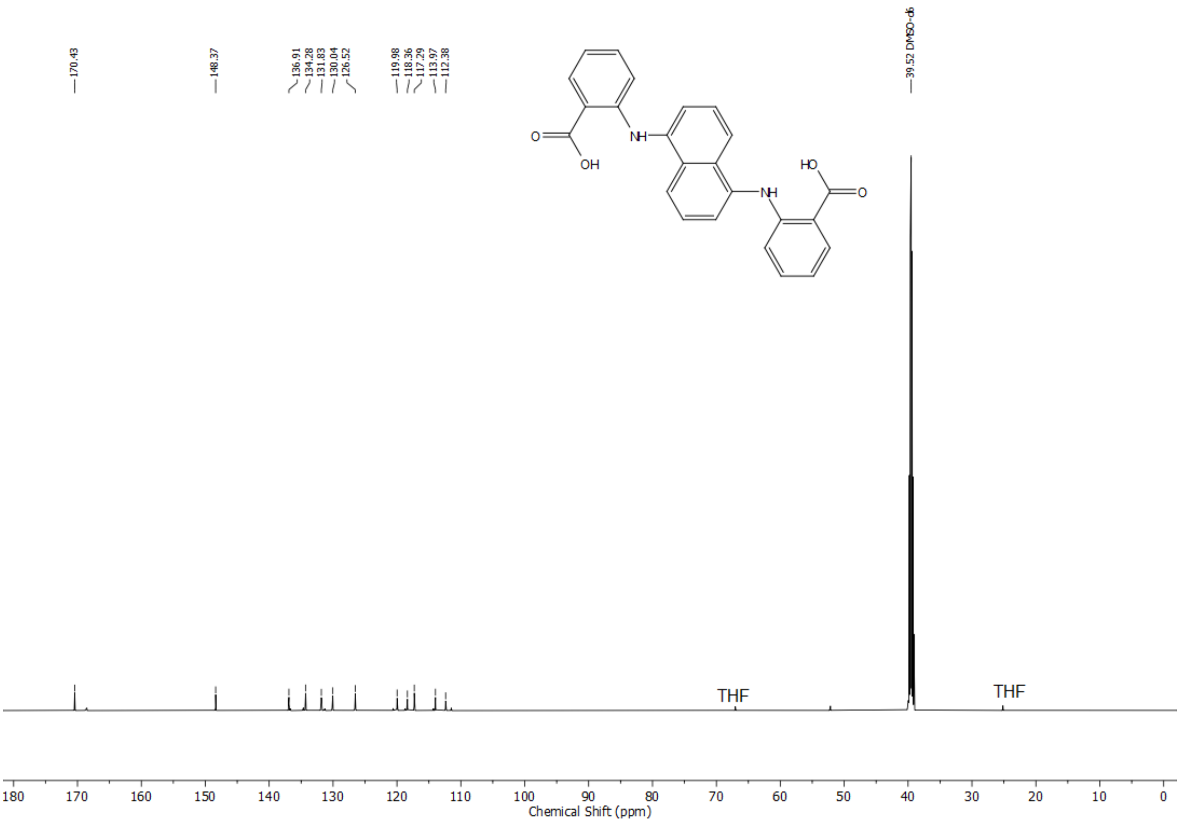


Figure S33. $^{13}C\{^1H\}$ NMR spectrum (151 MHz, DMSO) of **12**.

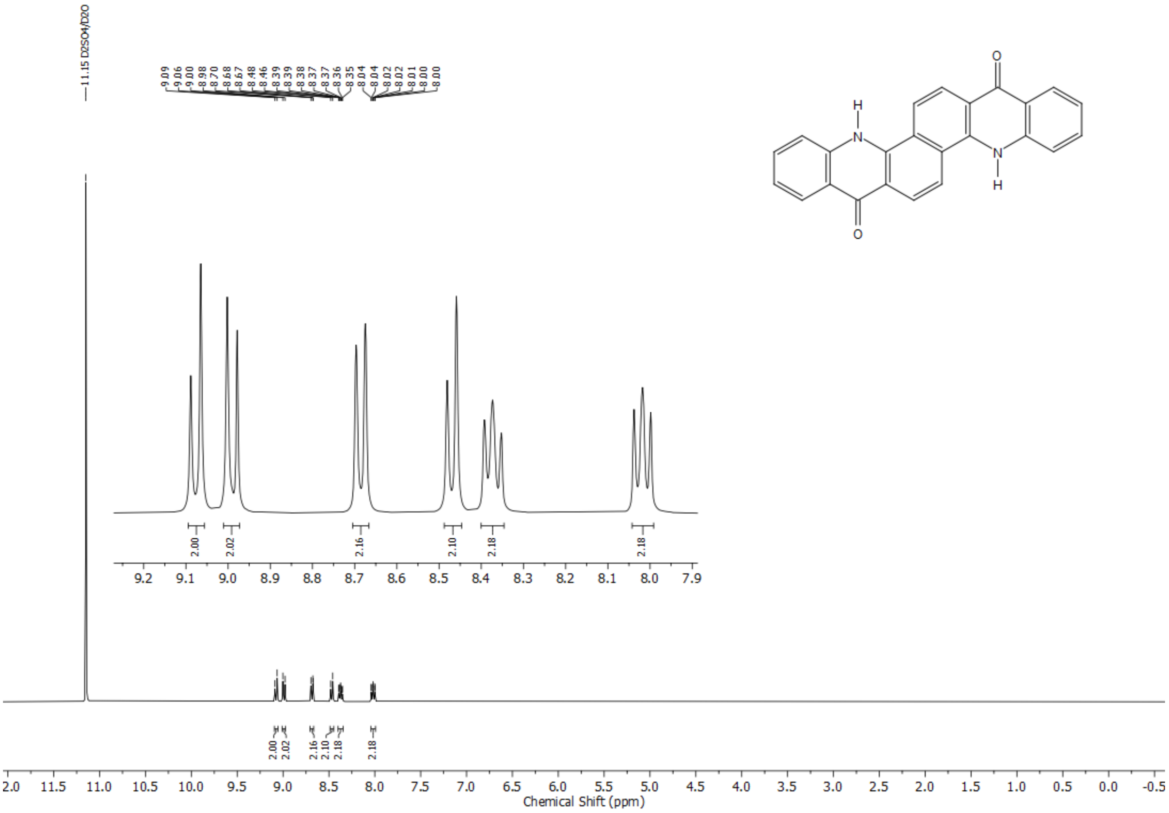


Figure S34. $^{1}H$ NMR spectrum (400 MHz, $D_2SO_4$) of **13**.

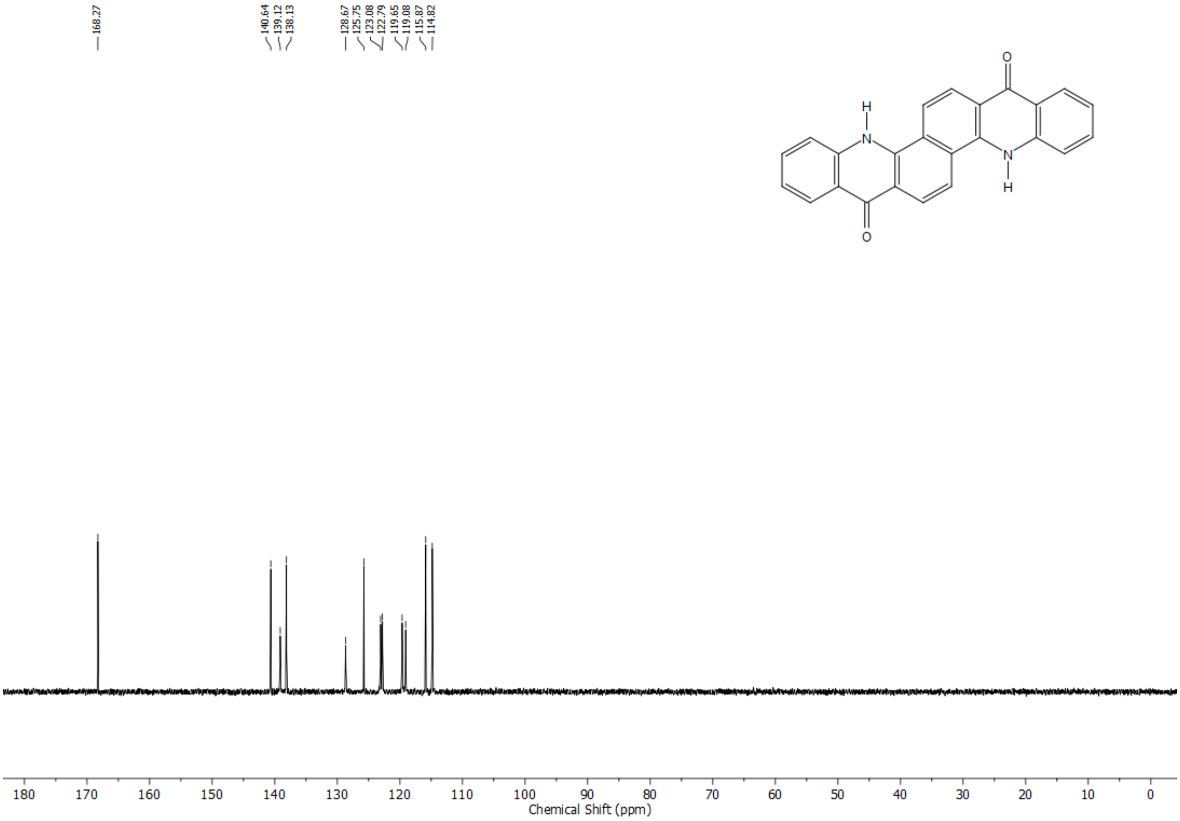


Figure S35. $^{13}C\{^{1}H\}$ NMR spectrum (101 MHz, $D_2SO_4$) of **13**.

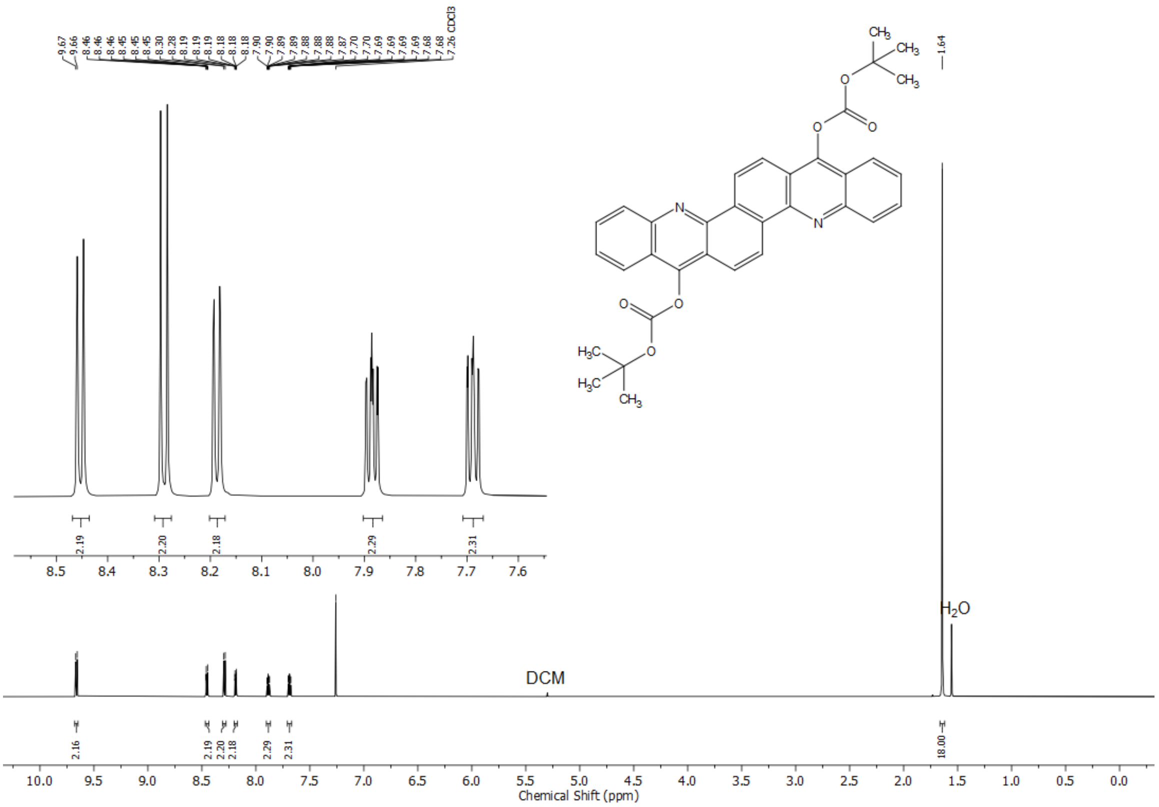

Figure S36. $^{1}H$ NMR spectrum (700 MHz, $CDCl_3$) of **14a**.

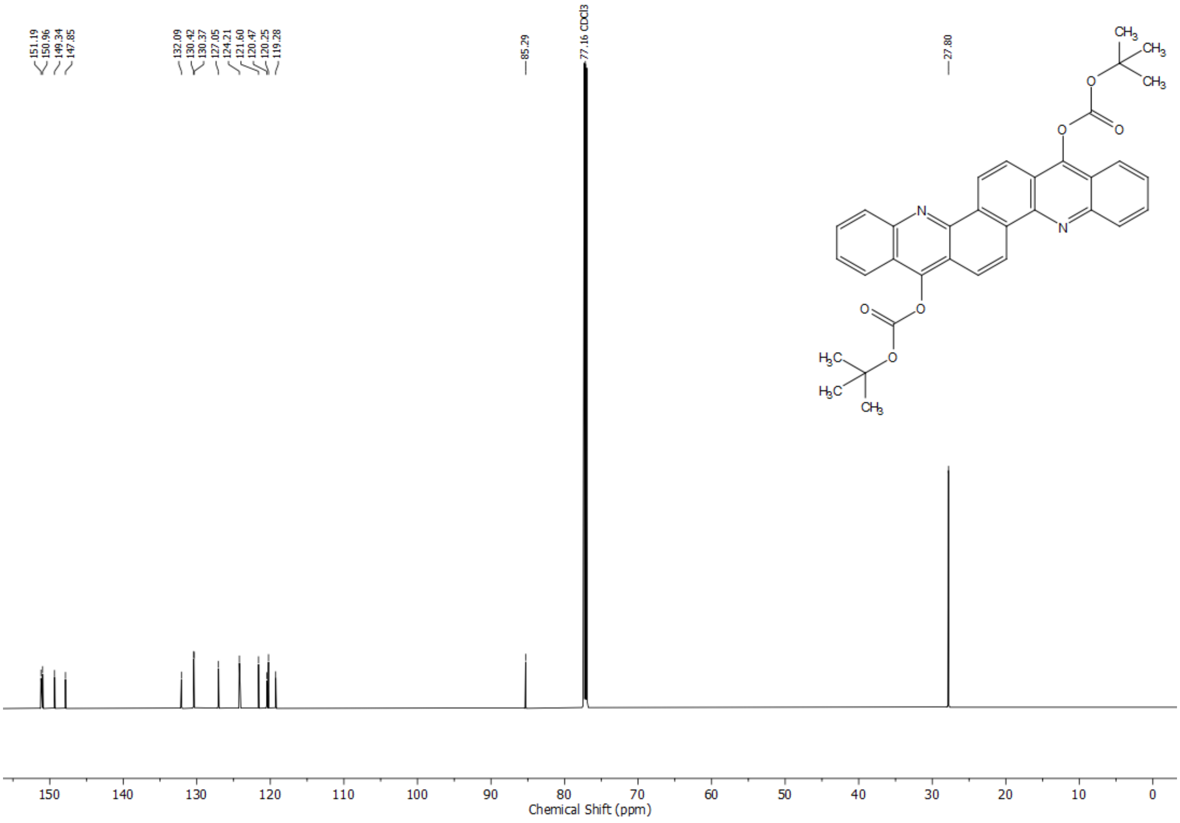

Figure S37. $^{13}C\{^{1}H\}$ NMR spectrum (176 MHz, $CDCl_3$) of **14a**.

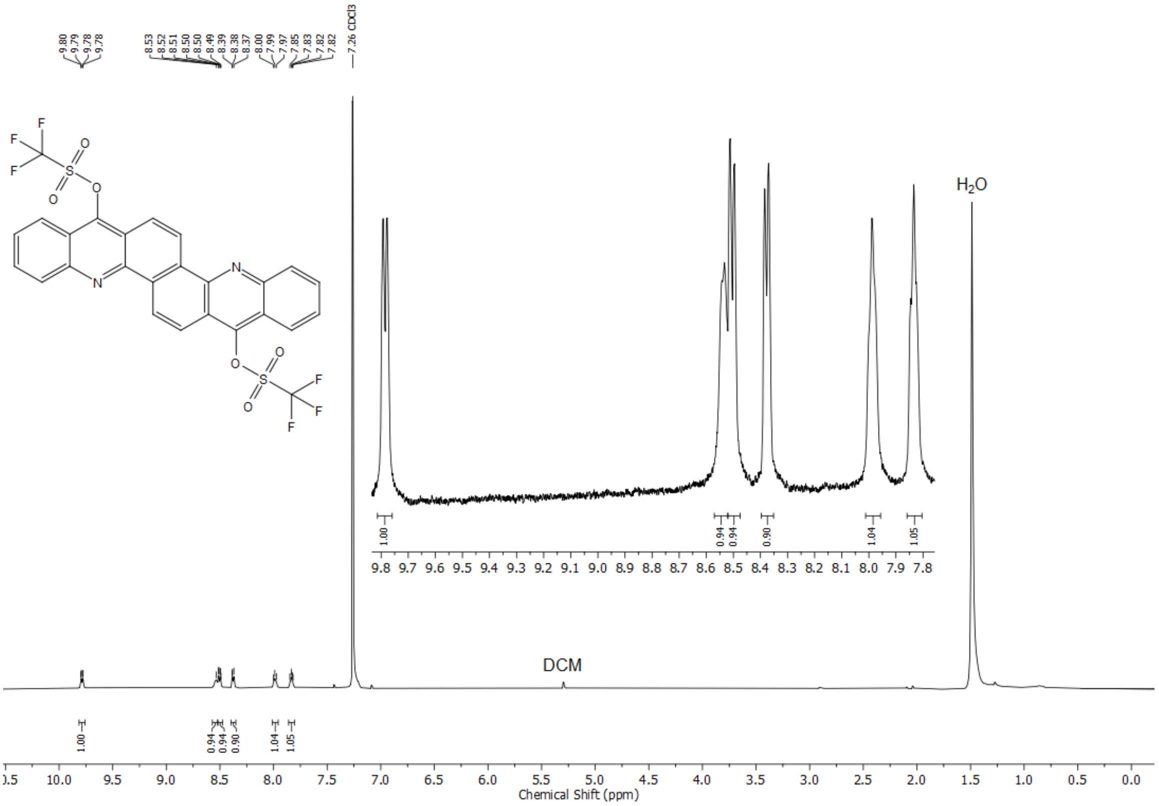


Figure S38. $^{1}$H NMR spectrum (600 MHz, CDCl$_3$) of **14b**.

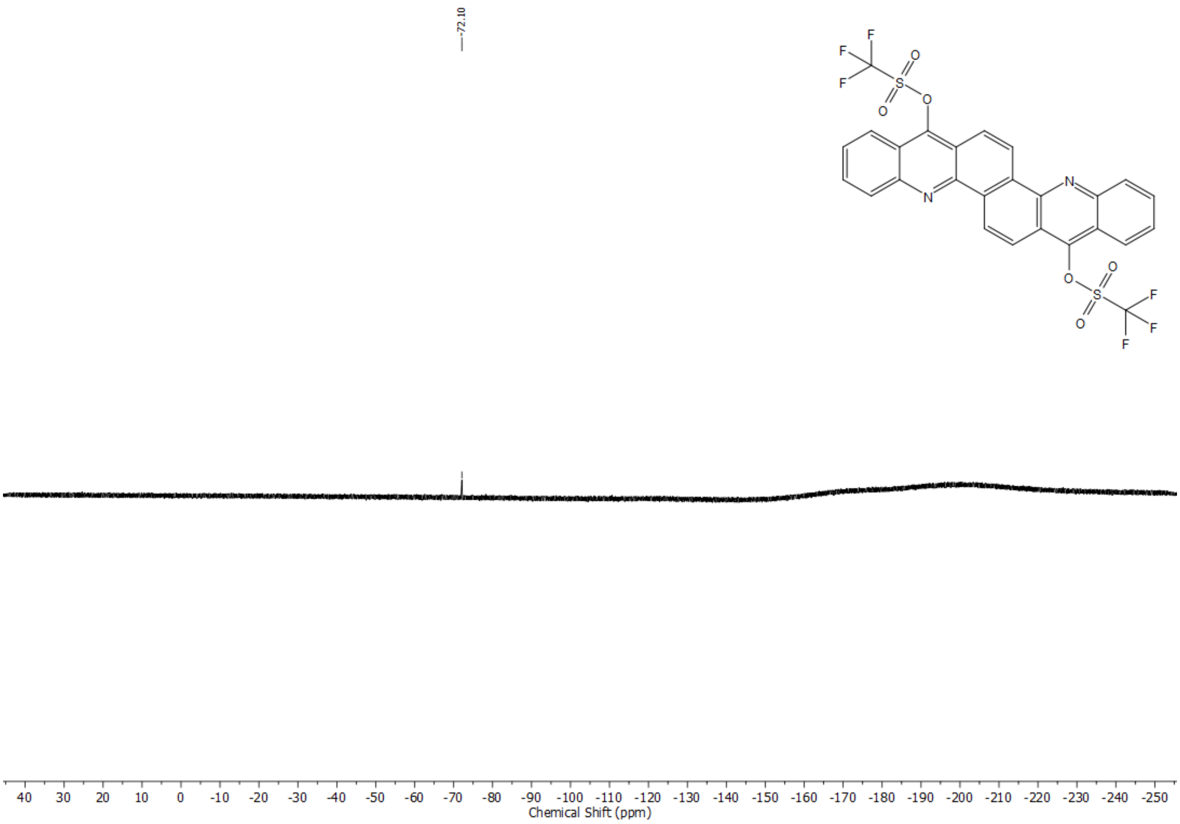


Figure S39. $^{19}$F{$^{13}$C} NMR spectrum (283 MHz, CDCl$_3$) of **14b**.

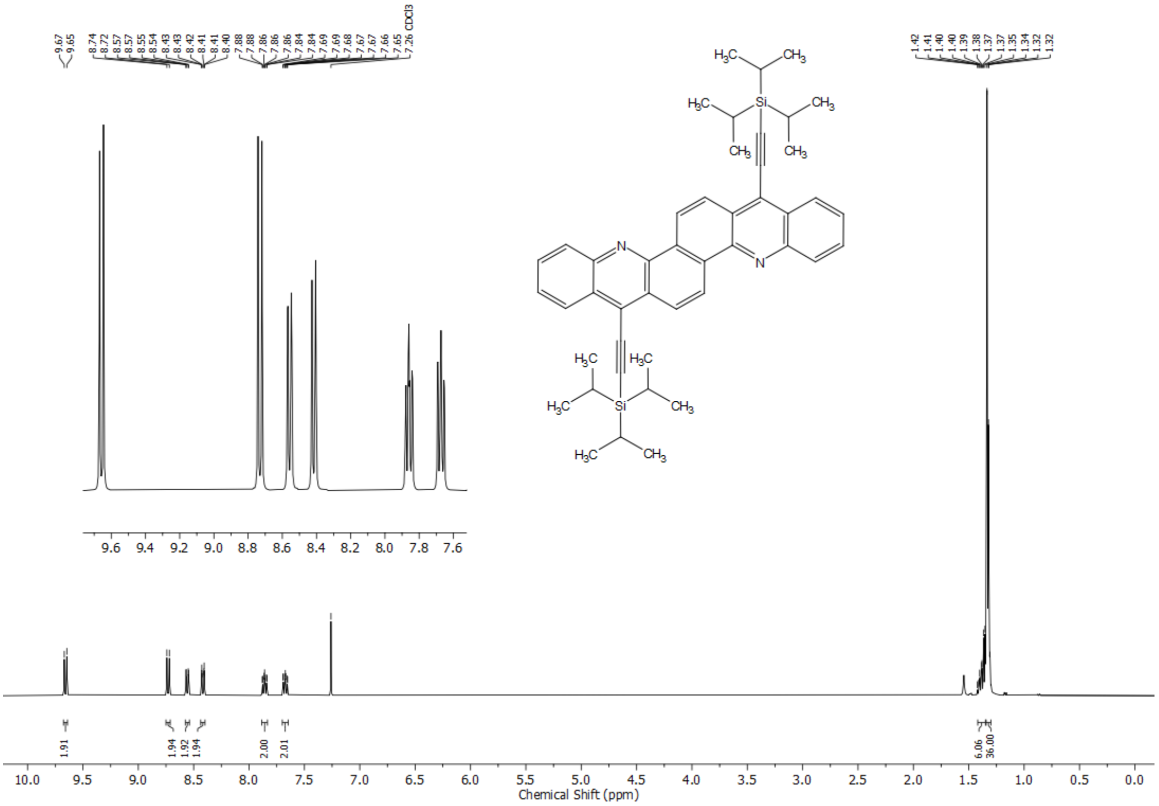

Figure S40. $^{1}H$ NMR spectrum (400 MHz, $CDCl_3$) of **4**.

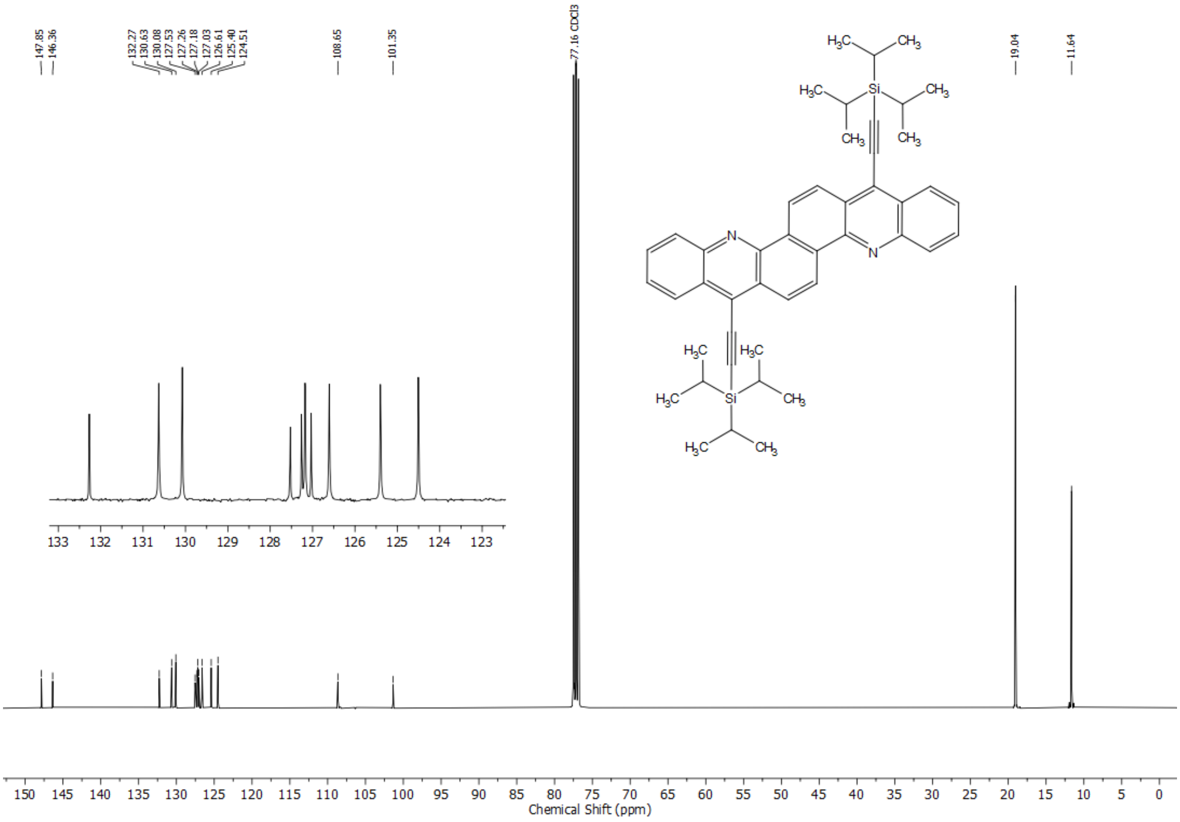

Figure S41. $^{13}C\{^{1}H\}$ NMR spectrum (101 MHz, $CDCl_3$) of **4**.

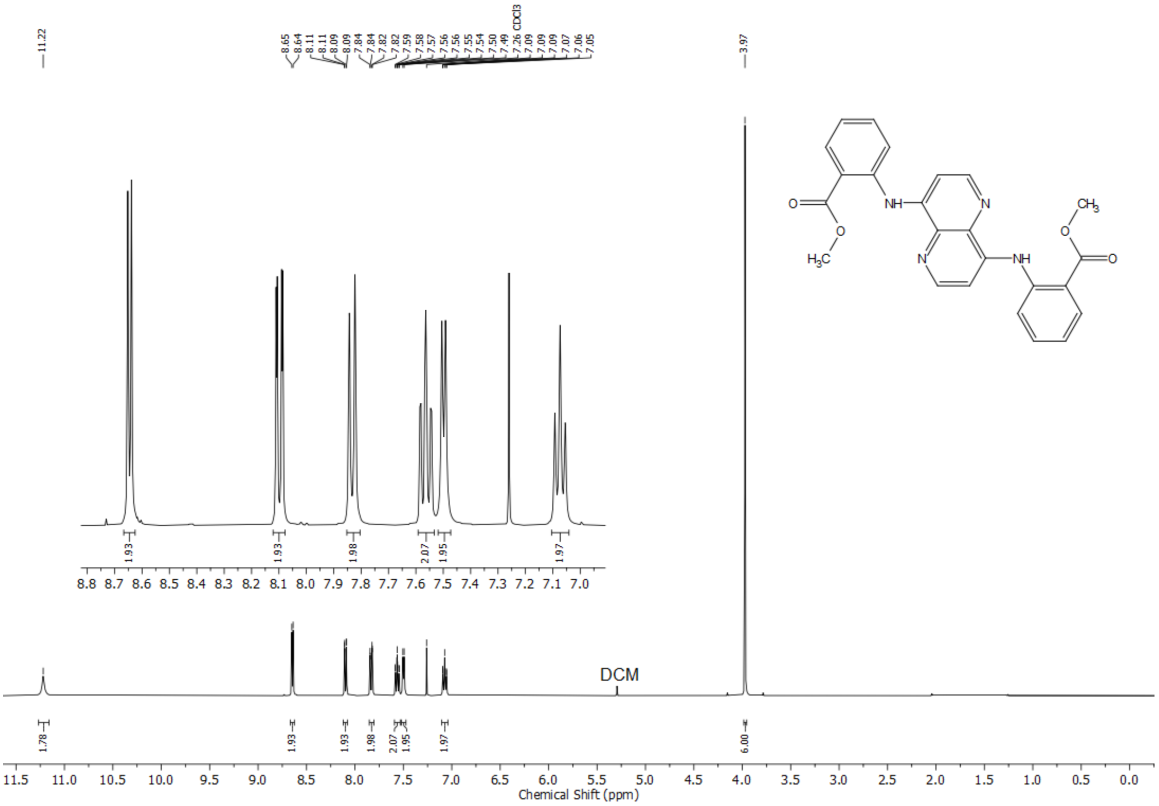


Figure S42. $^{1}H$ NMR spectrum (400 MHz, $CDCl_3$) of **S1**.

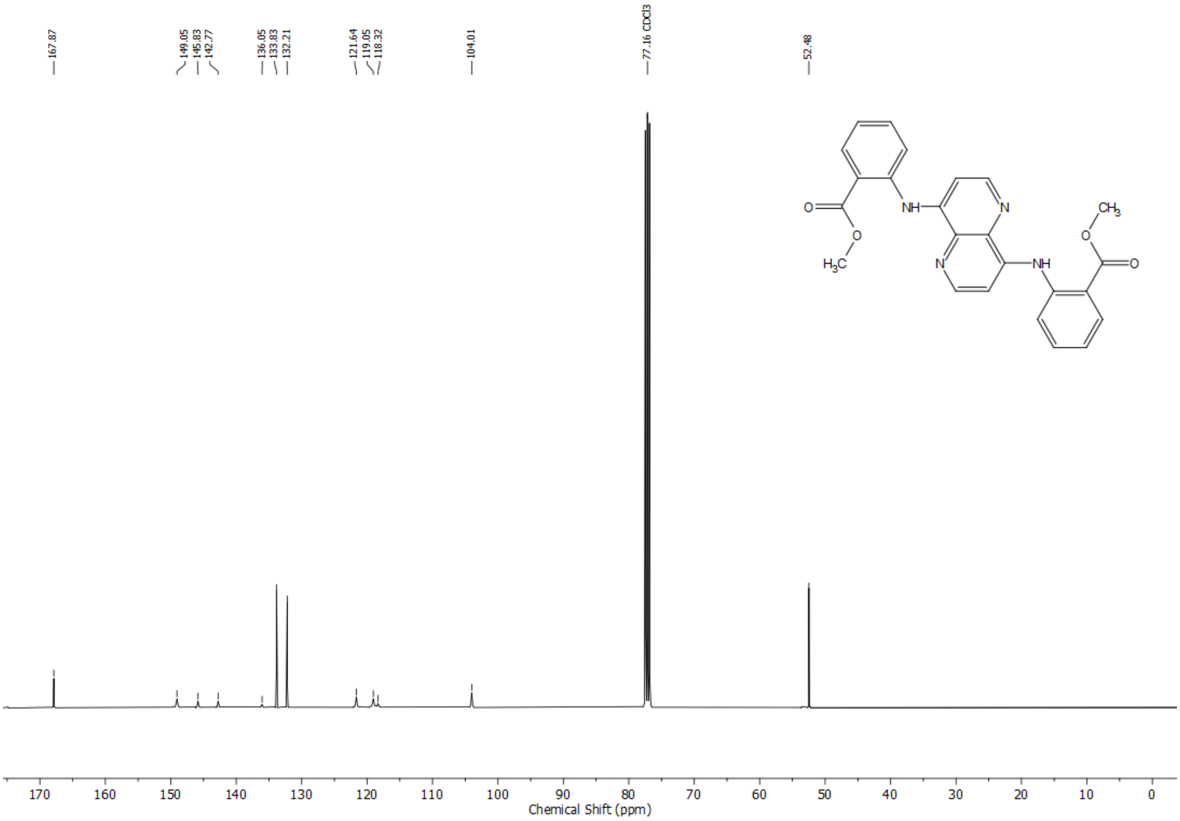


Figure S43. $^{13}C\{^{1}H\}$ NMR spectrum (101 MHz, $CDCl_3$) of **S1**.

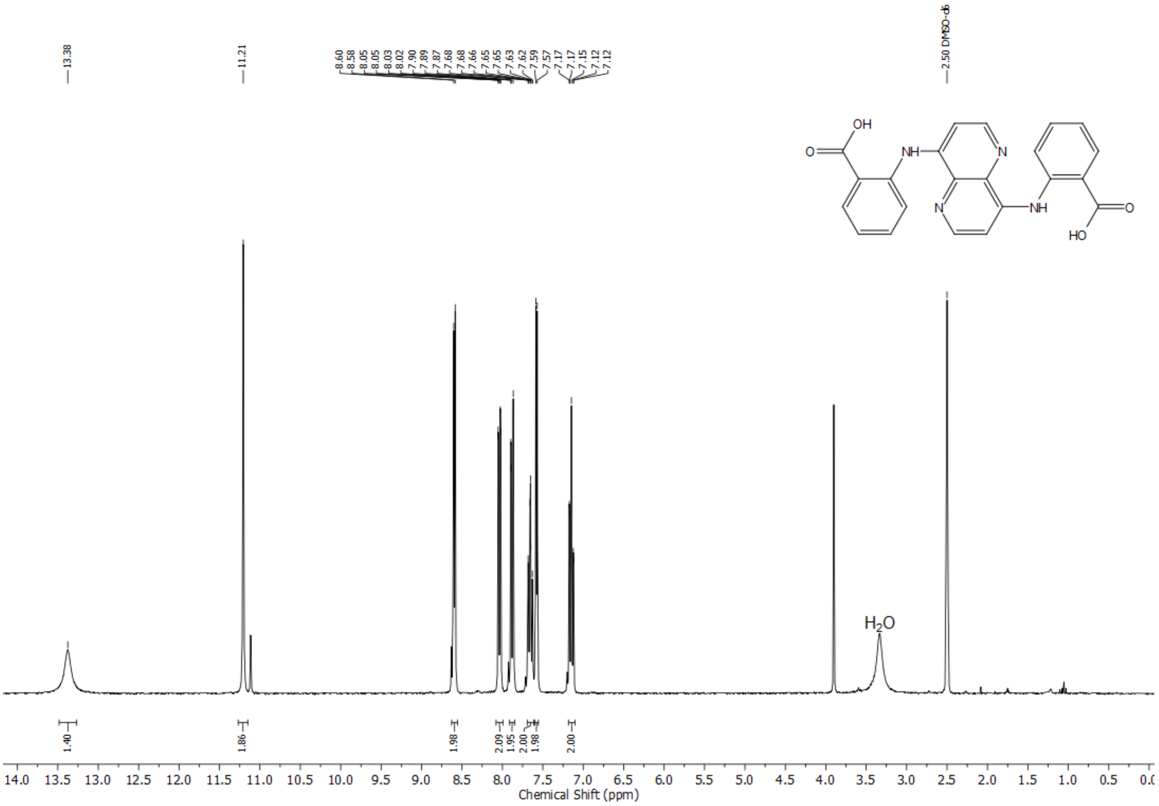

Figure S44. $^{1}H$ NMR spectrum (300 MHz, DMSO) of **S2**.

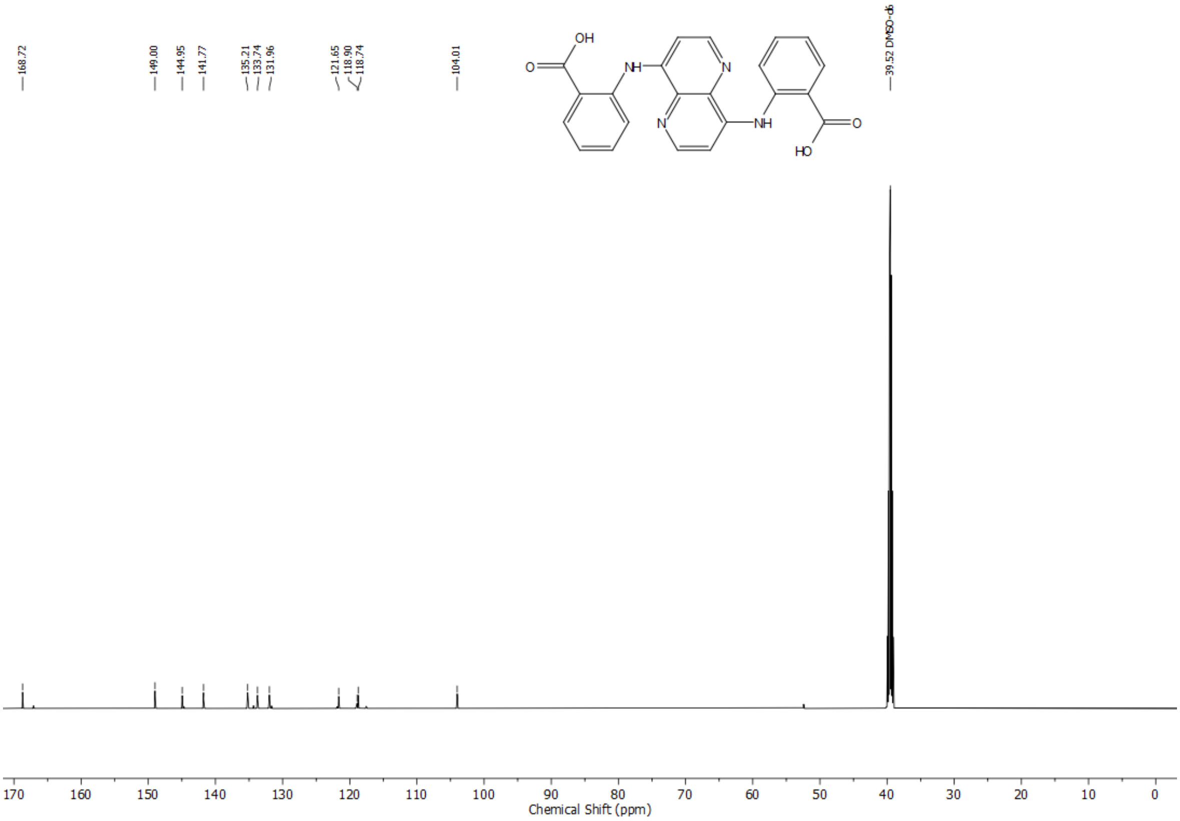

Figure S 45. $^{13}C\{^{1}H\}$ NMR spectrum (151 MHz, DMSO) of **S2**.

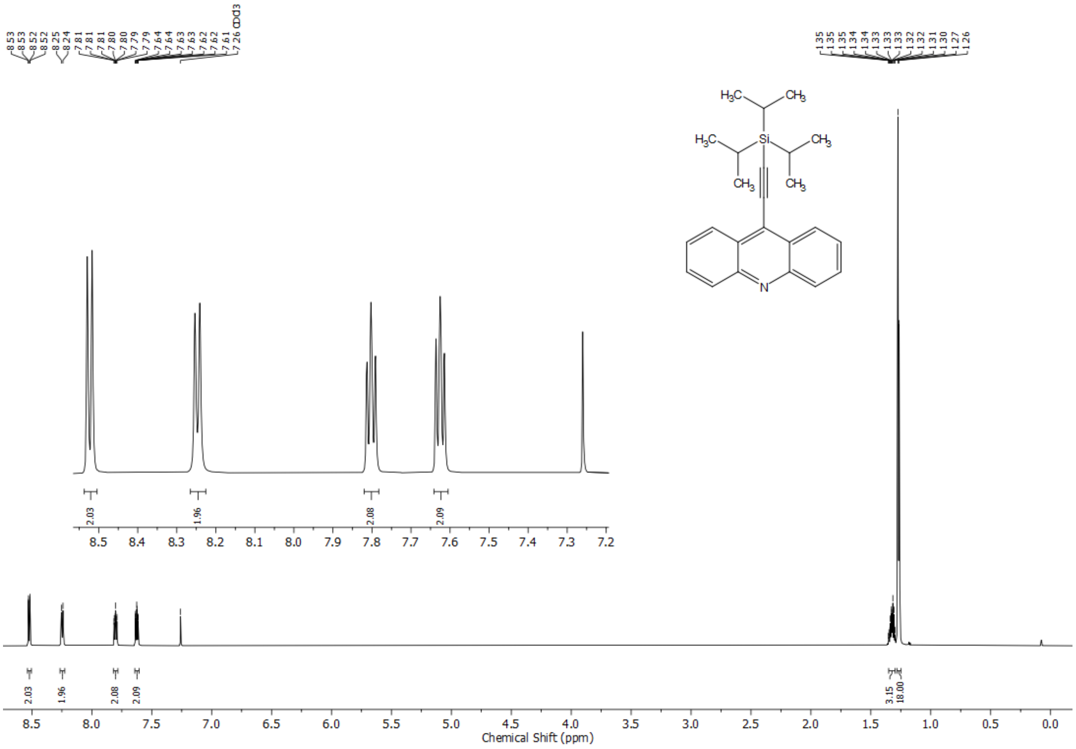

Figure S46. $^{1}H$ NMR spectrum (700 MHz, $CDCl_3$) of **15**.

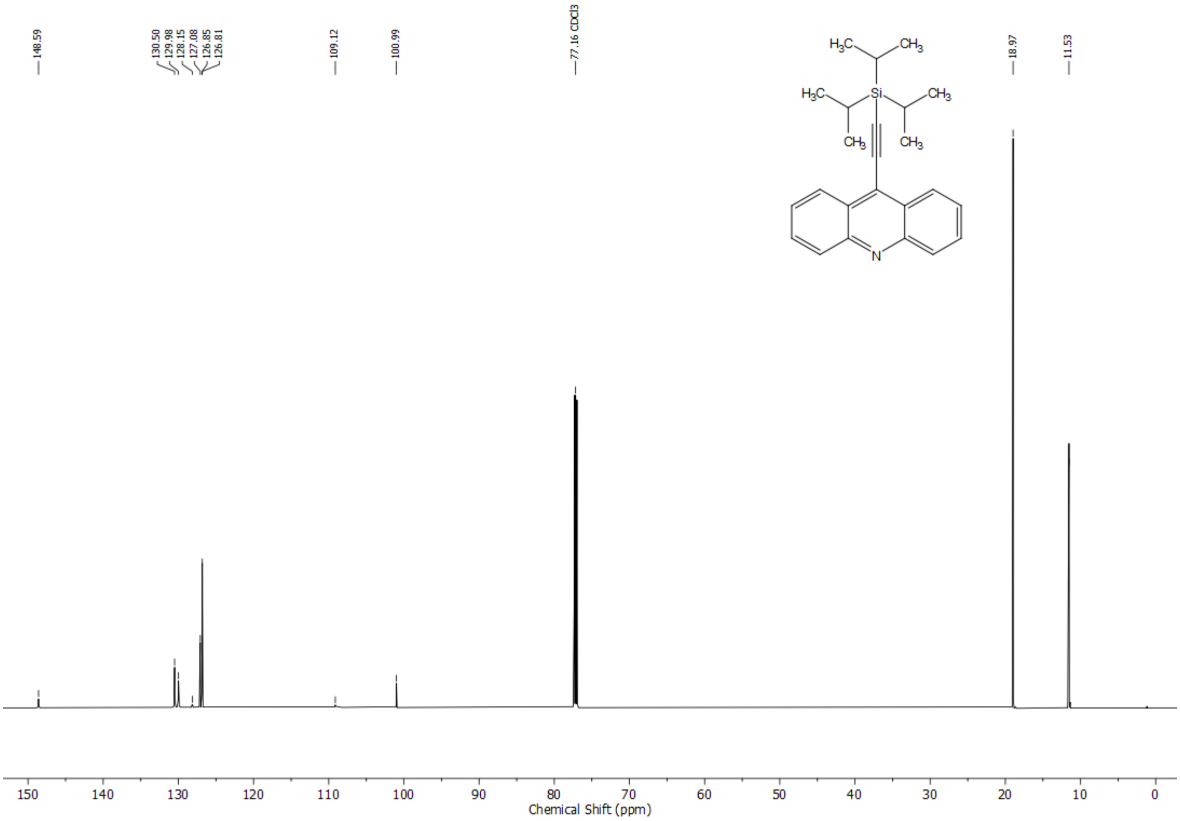

Figure S47. $^{13}C\{^{1}H\}$ NMR spectrum (176 MHz, $CDCl_3$) of **15**.

# 12. Crystallographic data

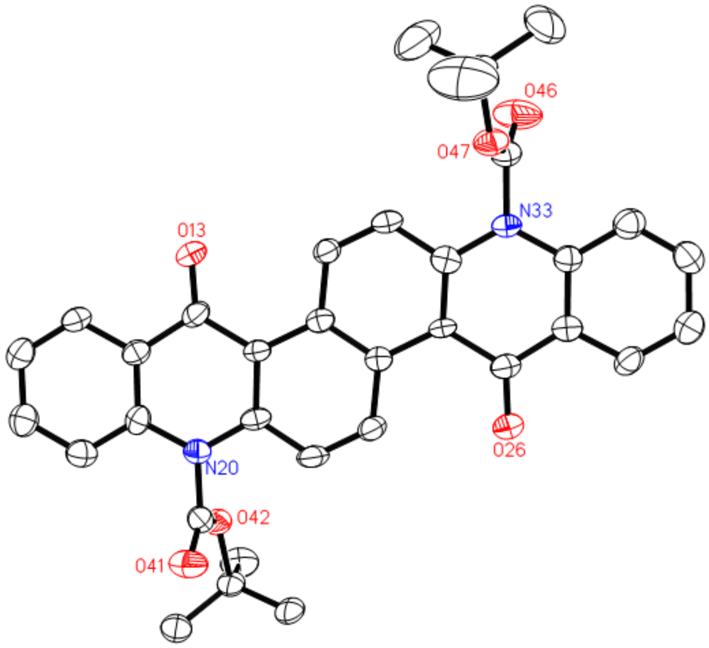


Figure S48. Crystal Structure of **9.**

Table S2. Crystal data and structure refinement for **9**.

| | | | |
|---|---|---|---|
| CCDC entry | 2155731 | | |
| Empirical formula | $C_{34}H_{30}N_2O_6$ | | |
| Formula weight | 562.60 | | |
| Temperature | 200(2) K | | |
| Wavelength | 1.54178 Å | | |
| Crystal system | orthorhombic | | |
| Space group | $Pca2_1$ | | |
| Z | 4 | | |
| Unit cell dimensions | a = 21.1570(7) Å | [illegible] = | 90 deg. |
| | b = 5.9033(2) Å | [illegible] = | 90 deg. |
| | c = 21.8796(10) Å | [illegible] = | 90 deg. |
| Volume | 2732.68(18) $Å^3$ | | |
| Density (calculated) | 1.37 $g/cm^3$ | | |
| Absorption coefficient | 0.77 $mm^{-1}$ | | |
| Crystal shape | needle | | |
| Crystal size | 0.343 x 0.031 x 0.020 $mm^3$ | | |
| Crystal colour | yellow | | |
| Theta range for data collection | 4.2 to 67.1 deg. | | |
| Index ranges | -24[illegible]h[illegible]24, -6[illegible]k[illegible]4, -25[illegible]l[illegible]25 | | |
| Reflections collected | 9772 | | |
| Independent reflections | 4002 (R(int) = 0.0470) | | |
| Observed reflections | 2861 (I > 2[illegible](I)) | | |
| Absorption correction | Semi-empirical from equivalents | | |
| Max. and min. transmission | 0.98 and 0.53 | | |
| Refinement method | Full-matrix least-squares on $F^2$ | | |
| Data/restraints/parameters | 4002 / 1 / 385 | | |
| Goodness-of-fit on $F^2$ | 0.96 | | |
| Final R indices (I>2sigma(I)) | R1 = 0.049, wR2 = 0.110 | | |
| Absolute structure parameter | 0.5(4) | | |
| Largest diff. peak and hole | 0.42 and -0.20 $eÅ^{-3}$ | | |

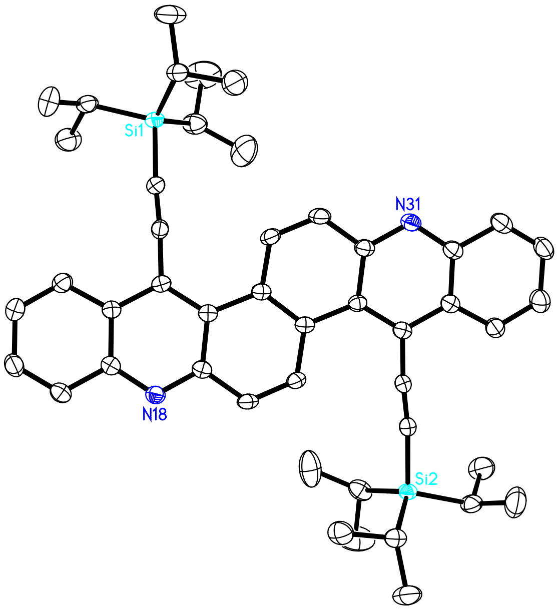


Figure S49. Crystal Structure of **3**.

Table S3. Crystal data and structure refinement for **3**.

| | | |
|---|---|---|
| CCDC entry | 2155728 | |
| Empirical formula | $C_{46}H_{54}N_2Si_2$ | |
| Formula weight | 691.09 | |
| Temperature | 200(2) K | |
| Wavelength | 0.71073 Å | |
| Crystal system | monoclinic | |
| Space group | $P2_1/c$ | |
| Z | 4 | |
| Unit cell dimensions | a = 16.5106(9) Å | □ = 90 deg. |
| | b = 13.5816(7) Å | □ = 90.7208(11) deg. |
| | c = 17.4908(9) Å | □ = 90 deg. |
| Volume | 3921.8(4) $Å^3$ | |
| Density (calculated) | 1.17 $g/cm^3$ | |
| Absorption coefficient | 0.12 $mm^{-1}$ | |
| Crystal shape | plate | |
| Crystal size | 0.183 x 0.166 x 0.015 $mm^3$ | |
| Crystal colour | yellow | |
| Theta range for data collection | 1.2 to 29.8 deg. | |
| Index ranges | -23□h□23, -18□k□18, -24□l□24 | |
| Reflections collected | 47243 | |
| Independent reflections | 10545 (R(int) = 0.0696) | |
| Observed reflections | 6283 (I > 2□(I)) | |
| Absorption correction | Semi-empirical from equivalents | |
| Max. and min. transmission | 0.96 and 0.91 | |
| Refinement method | Full-matrix least-squares on $F^2$ | |
| Data/restraints/parameters | 10545 / 0 / 463 | |
| Goodness-of-fit on $F^2$ | 1.01 | |
| Final R indices (I>2sigma(I)) | R1 = 0.054, wR2 = 0.122 | |
| Largest diff. peak and hole | 0.33 and -0.27 $eÅ^{-3}$ | |

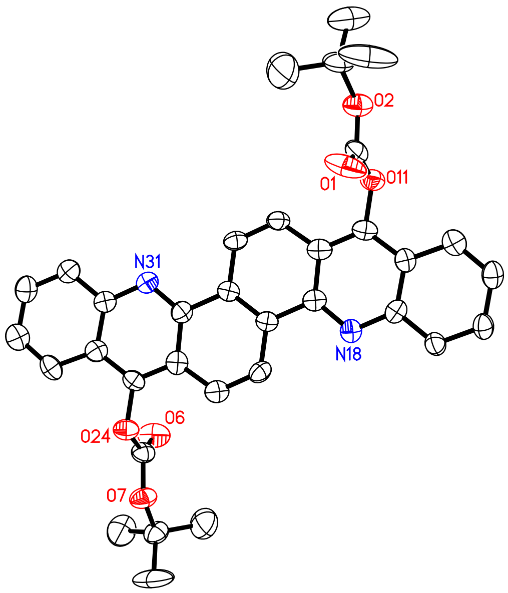


Figure S50. Crystal Structure of **14a**.

Table S4. Crystal data and structure refinement for **14a**.

| | | |
|---|---|---|
| CCDC entry | 2155733 | |
| Empirical formula | $C_{34}H_{30}N_2O_6$ | |
| Formula weight | 562.60 | |
| Temperature | 200(2) K | |
| Wavelength | 1.54178 Å | |
| Crystal system | orthorhombic | |
| Space group | $Pca2_1$ | |
| Z | 4 | |
| Unit cell dimensions | a = 20.3238(13) Å | □ = 90 deg. |
| | b = 5.8376(3) Å | □ = 90 deg. |
| | c = 24.1026(16) Å | □ = 90 deg. |
| Volume | 2859.6(3) $Å^3$ | |
| Density (calculated) | 1.31 $g/cm^3$ | |
| Absorption coefficient | 0.73 $mm^{-1}$ | |
| Crystal shape | needle | |
| Crystal size | 0.365 x 0.015 x 0.015 $mm^3$ | |
| Crystal colour | yellow | |
| Theta range for data collection | 7.6 to 61.5 deg. | |
| Index ranges | -22□h□21, -6□k□3, -22□l□26 | |
| Reflections collected | 8418 | |
| Independent reflections | 3282 (R(int) = 0.0621) | |
| Observed reflections | 2013 (I > 2□(I)) | |
| Absorption correction | Semi-empirical from equivalents | |
| Max. and min. transmission | 0.99 and 0.34 | |
| Refinement method | Full-matrix least-squares on $F^2$ | |
| Data/restraints/parameters | 3282 / 904 / 530 | |
| Goodness-of-fit on $F^2$ | 0.93 | |
| Final R indices (I>2sigma(I)) | R1 = 0.054, wR2 = 0.111 | |
| Absolute structure parameter | -0.1(4) | |
| Largest diff. peak and hole | 0.24 and -0.18 $eÅ^{-3}$ | |

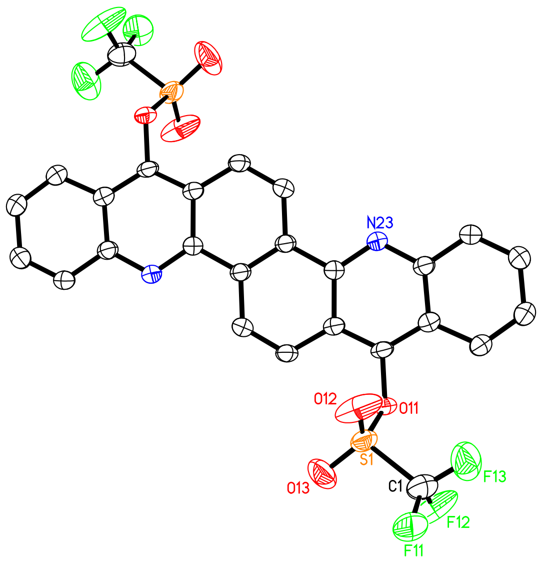


Figure S51. Crystal Structure of **14b**.

Table S5. Crystal data and structure refinement for **14b**.

| | | | |
|---|---|---|---|
| CCDC entry | 2155732 | | |
| Empirical formula | $C_{26}H_{12}F_6N_2O_6S_2$ | | |
| Formula weight | 626.50 | | |
| Temperature | 200(2) K | | |
| Wavelength | 1.54178 Å | | |
| Crystal system | triclinic | | |
| Space group | $P\bar{1}$ | | |
| Z | 1 | | |
| Unit cell dimensions | a = 5.9285(4) Å | α = | 87.570(6) deg. |
| | b = 7.4547(5) Å | β = | 82.992(6) deg. |
| | c = 13.8272(10) Å | γ = | 84.825(6) deg. |
| Volume | 603.75(7) $Å^3$ | | |
| Density (calculated) | 1.72 $g/cm^3$ | | |
| Absorption coefficient | 2.89 $mm^{-1}$ | | |
| Crystal shape | needle | | |
| Crystal size | 0.220 x 0.018 x 0.012 $mm^3$ | | |
| Crystal colour | yellow | | |
| Theta range for data collection | 6.0 to 66.8 deg. | | |
| Index ranges | $-6 \le h \le 4$, $-8 \le k \le 8$, $-16 \le l \le 15$ | | |
| Reflections collected | 5131 | | |
| Independent reflections | 2036 (R(int) = 0.0325) | | |
| Observed reflections | 1420 ($I > 2\sigma(I)$) | | |
| Absorption correction | Semi-empirical from equivalents | | |
| Max. and min. transmission | 0.96 and 0.21 | | |
| Refinement method | Full-matrix least-squares on $F^2$ | | |
| Data/restraints/parameters | 2036 / 12 / 190 | | |
| Goodness-of-fit on $F^2$ | 1.03 | | |
| Final R indices (I>2sigma(I)) | R1 = 0.052, wR2 = 0.132 | | |
| Largest diff. peak and hole | 0.43 and -0.39 $eÅ^{-3}$ | | |

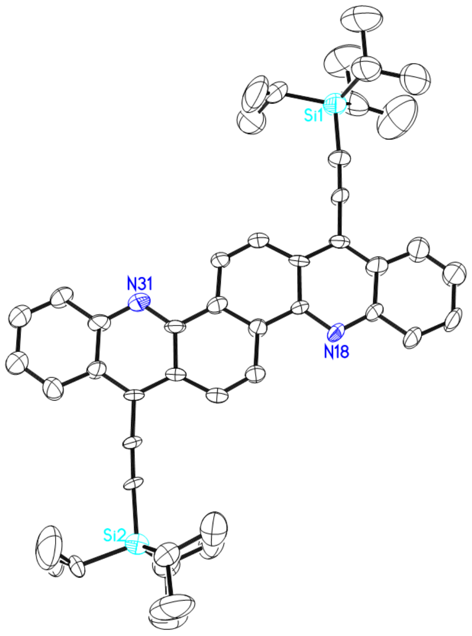


Figure S52. Crystal Structure of **4** (Polymorph A).

Table S6.  Crystal data and structure refinement for **4** (Polymorph A; measured at room temperature).

| | | |
|---|---|---|
| CCDC entry | 2155729 | |
| Empirical formula | $C_{46}H_{54}N_2Si_2$ | |
| Formula weight | 691.09 | |
| Temperature | 294(2) K | |
| Wavelength | 0.71073 Å | |
| Crystal system | monoclinic | |
| Space group | $P2_1/c$ | |
| Z | 4 | |
| Unit cell dimensions | a = 13.624(5) Å | α = 90 deg. |
| | b = 33.905(11) Å | β = 105.359(9) deg. |
| | c = 9.136(3) Å | γ = 90 deg. |
| Volume | 4069(2) $Å^3$ | |
| Density (calculated) | 1.13 $g/cm^3$ | |
| Absorption coefficient | 0.12 $mm^{-1}$ | |
| Crystal shape | plate | |
| Crystal size | 0.080 x 0.047 x 0.032 $mm^3$ | |
| Crystal colour | yellow | |
| Theta range for data collection | 1.5 to 19.8 deg. | |
| Reflections collected | 23495 | |
| Independent reflections | 3345 (R(int) = 0.3067) | |
| Observed reflections | 1506 (I > 2σ(I)) | |
| Absorption correction | Semi-empirical from equivalents | |
| Max. and min. transmission | 0.88 and 0.80 | |
| Refinement method | Full-matrix least-squares on $F^2$ | |
| Data/restraints/parameters | 3345 / 632 / 451 | |
| Goodness-of-fit on $F^2$ | 1.50 | |
| Final R indices (I>2sigma(I)) | R1 = 0.182, wR2 = 0.325 | |
| Largest diff. peak and hole | 0.56 and -0.39 $eÅ^{-3}$ | |

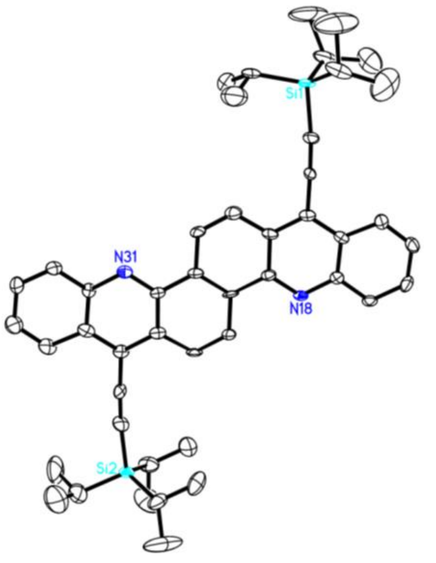


Figure S53. Crystal Structure of **4** (Polymorph B).

Table S7. Crystal data and structure refinement for **4** (Polymorph B, measured at 200 K).

| | | |
|---|---|---|
| CCDC entry | 2155730 | |
| Summenformel | $C_{46}H_{54}N_2Si_2$ | |
| Molmasse | 691.09 | |
| Temperatur | 200(2) K | |
| Wellenlänge | 0.71073 Å | |
| Kristallsystem | monoklin | |
| Raumgruppe | $P2_1/c$ | |
| Z | 8 | |
| Gitterkonstanten | a = 34.761(4) Å | □ = 90 ° |
| | b = 13.9877(15) Å | □ = 103.933(3) ° |
| | c = 16.7419(18) Å | □ = 90 ° |
| Zellvolumen | 7900.8(16) $Å^3$ | |
| Dichte (berechnet) | 1.162 $g/cm^3$ | |
| Absorptionskoeffizient µ | 0.124 $mm^{-1}$ | |
| Kristallform | plate | |
| Kristallgröße | 0.074 x 0.050 x 0.037 $mm^3$ | |
| Kristallfarbe | yellow | |
| Gemessener Theta-Bereich | 0.604 bis 24.407 ° | |
| Indexgrenzen | -40□h□40, -16□k□16, -19□l□19 | |
| Gemessene Reflexe | 72938 | |
| Unabhängige Reflexe | 12969 (R(int) = 0.1826) | |
| Beobachtete Reflexe | 5590 (I > 2□(I)) | |
| Absorptionskorrektur | Semi-empirical from equivalents | |
| Max/min Transmission | 0.96 and 0.84 | |
| Strukturverfeinerung | Full-matrix least-squares an $F^2$ | |
| Daten/Restraints/Parameter | 12969 / 2693 / 902 | |
| Goodness-of-fit an $F^2$ | 1.16 | |
| R-Werte (I>2sigma(I)) | R1 = 0.096, wR2 = 0.233 | |
| Extinktionskoeffizient | n/a | |
| Max/min Restelektronendichte | 0.75 und -0.50 $eÅ^{-3}$ | |

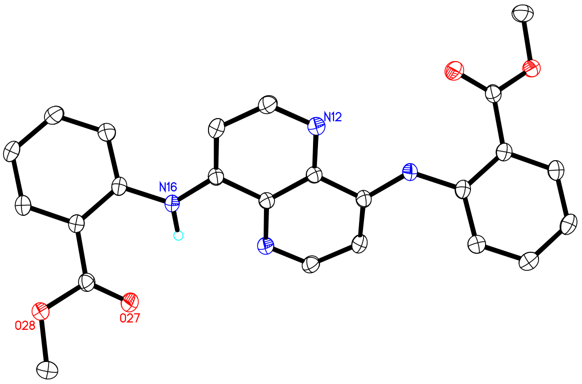


Figure S 54. Crystal Structure of **S1**.

Table S8. Crystal data and structure refinement for **S1**.

| | |
|---|---|
| CCDC entry | 2155734 |
| Empirical formula | $C_{24}H_{20}N_4O_4$ |
| Formula weight | 428.44 |
| Temperature | 200(2) K |
| Wavelength | 0.71073 Å |
| Crystal system | triclinic |
| Space group | $P\bar{1}$ |
| Z | 1 |
| Unit cell dimensions | a = 5.0053(5) Å [illegible] = 77.5630(14) deg. |
| | b = 8.4041(8) Å [illegible] = 80.9177(15) deg. |
| | c = 12.5761(12) Å [illegible] = 75.5361(15) deg. |
| Volume | 497.19(8) $Å^3$ |
| Density (calculated) | 1.43 $g/cm^3$ |
| Absorption coefficient | 0.10 $mm^{-1}$ |
| Crystal shape | rhombic |
| Crystal size | 0.180 x 0.117 x 0.046 $mm^3$ |
| Crystal colour | yellow |
| Theta range for data collection | 1.7 to 29.7 deg. |
| Index ranges | -6[illegible]h[illegible]6, -11[illegible]k[illegible]11, -17[illegible]l[illegible]16 |
| Reflections collected | 7970 |
| Independent reflections | 2605 (R(int) = 0.0210) |
| Observed reflections | 2281 (I > 2[illegible](I)) |
| Absorption correction | Semi-empirical from equivalents |
| Max. and min. transmission | 0.96 and 0.91 |
| Refinement method | Full-matrix least-squares on $F^2$ |
| Data/restraints/parameters | 2605 / 0 / 150 |
| Goodness-of-fit on $F^2$ | 1.06 |
| Final R indices (I>2sigma(I)) | R1 = 0.041, wR2 = 0.115 |
| Largest diff. peak and hole | 0.36 and -0.24 $eÅ^{-3}$ |

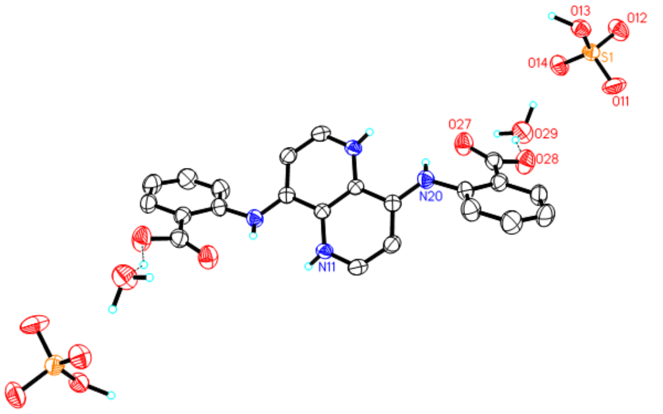


Figure S 55. Crystal structure of **S2.**

Table S9. Crystal data and structure refinement for **S2**.

| | | |
|---|---|---|
| Identification code | 2155735 | |
| Empirical formula | $C_{22}H_{24}N_4O_{14}S_2$ | |
| Formula weight | 632.57 | |
| Temperature | 200(2) K | |
| Wavelength | 1.54178 Å | |
| Crystal system | triclinic | |
| Space group | P$\bar{1}$ | |
| Z | 1 | |
| Unit cell dimensions | a = 7.6223(4) Å | □ = 78.248(4) deg. |
| | b = 8.1298(4) Å | □ = 88.591(4) deg. |
| | c = 10.8754(5) Å | □ = 89.850(4) deg. |
| Volume | 659.60(6) $Å^3$ | |
| Density (calculated) | 1.59 $g/cm^3$ | |
| Absorption coefficient | 2.56 $mm^{-1}$ | |
| Crystal shape | plate | |
| Crystal size | 0.080 x 0.042 x 0.015 $mm^3$ | |
| Crystal colour | yellow | |
| Theta range for data collection | 4.2 to 66.9 deg. | |
| Index ranges | -9□h□8, -6□k□9, -10□l□12 | |
| Reflections collected | 5674 | |
| Independent reflections | 2238 (R(int) = 0.0213) | |
| Observed reflections | 1809 (I > 2□(I)) | |
| Absorption correction | Semi-empirical from equivalents | |
| Max. and min. transmission | 0.99 and 0.56 | |
| Refinement method | Full-matrix least-squares on $F^2$ | |
| Data/restraints/parameters | 2238 / 0 / 207 | |
| Goodness-of-fit on $F^2$ | 1.10 | |
| Final R indices (I>2sigma(I)) | R1 = 0.054, wR2 = 0.134 | |
| Largest diff. peak and hole | 0.71 and -0.31 $eÅ^{-3}$ | |

[1] L. Ahrens, S. Hahn, F. Rominger, J. Freudenberg, U. H. F. Bunz, *Chem. Eur. J.* **2019**, *25*, 14522-14526.
[2] a) R. Herges, D. Geuenich, J. Phys. Chem. A 2001, 105, 3214-3220; b) D. Geuenich, K. Hess, F. Köhler, R. Herges, *Chem. Rev.* **2005**, *105*, 3758-3772.
[3] P. R. Spackman, M. J. Turner, J. J. McKinnon, S. K. Wolff, D. J. Grimwood, D. Jayatilaka, M. A. Spackman, *J. Appl. Crystallogr.* **2021**, *54*, 1006-1011.
[4] M. Chu, J. X. Fan, S. Yang, D. Liu, C. F. Ng, H. Dong, A. M. Ren, Q. Miao, *Adv. Mater.* **2018**, *30*, e1803467.
[5] V. Stehr, J. Pfister, R. F. Fink, B. Engels, C. Deibel, *Phys. Rev. B* **2011**, *83*, 155208.
[6] R. A. Marcus, *Rev. Mod. Phys.* **1993**, *65*, 599-610.
[7] a) S.-H. Wen, A. Li, J. Song, W.-Q. Deng, K.-L. Han, W. A. Goddard, *J. Phys. Chem. B* **2009**, *113*, 8813-8819; b) A. N. Sokolov, S. Atahan-Evrenk, R. Mondal, H. B. Akkerman, R. S. SánchezCarrera, S. Granados-Focil, J. Schrier, S. C. B. Mannsfeld, A. P. Zoombelt, Z. Bao, A. Aspuru-Guzik, *Nat: Commun.* **2011**, *2*, 437.
[8] a) S.-H. Wen, A. Li, J. Song, W.-Q. Deng, K.-L. Han, W. A. Goddard, *J. Phys. Chem. B* **2009**, *113*, 8813-8819; M. Chu, J.-X. Fan, S. Yang, D. Liu, C. F. Ng, H. Dong, A.-M. Ren, Q. Miao, *Adv. Mater.* **2018**, *30*, 1803467.
[9] P. E. Eaton, G. R. Carlson, J. T. Lee, *The Journal of Organic Chemistry* **2002**, *38*, 4071-4073.
[10] M. Balkenhohl, R. Greiner, I. S. Makarov, B. Heinz, K. Karaghiosoff, H. Zipse, P. Knochel, *Chem.* **2017**, *23*, 13046-13050.
